# Determinants of brain swelling in pediatric and adult cerebral malaria

**DOI:** 10.1172/jci.insight.145823

**Published:** 2021-09-22

**Authors:** Praveen K. Sahu, Fergal J. Duffy, Selasi Dankwa, Maria Vishnyakova, Megharay Majhi, Lukas Pirpamer, Vladimir Vigdorovich, Jabamani Bage, Sameer Maharana, Wilson Mandala, Stephen J. Rogerson, Karl B. Seydel, Terrie E. Taylor, Kami Kim, D. Noah Sather, Akshaya Mohanty, Rashmi R. Mohanty, Anita Mohanty, Rajyabardhan Pattnaik, John D. Aitchison, Angelika Hoffman, Sanjib Mohanty, Joseph D. Smith, Maria Bernabeu, Samuel C. Wassmer

**Affiliations:** 1 Center for the Study of Complex Malaria in India, Ispat General Hospital (IGH), Rourkela, Odisha, India.; 2 Seattle Children’s Research Institute, Seattle, Washington, USA.; 3 Department of Radiology, IGH, Rourkela, Odisha, India.; 4 Department of Infection Biology, London School of Hygiene & Tropical Medicine, London, United Kingdom.; 5 Malawi University of Science and Technology, Limbe, Malawi.; 6 Department of Medicine, The Doherty Institute, University of Melbourne, Melbourne, Australia.; 7 Department of Osteopathic Medical Specialties, College of Osteopathic Medicine, Michigan State University, East Lansing, Michigan, USA.; 8 Blantyre Malaria Project, Kamuzu University of Health Sciences, Blantyre, Malawi.; 9 Division of Infectious Diseases and International Medicine, Morsani College of Medicine, University of South Florida, Tampa, Florida, USA.; 10 Department of Pediatrics, University of Washington, Seattle, Washington, USA.; 11 Infectious Diseases Biology Unit, Institute of Life Sciences, Bhubaneswar, Odisha, India.; 12 Department of Ophthalmology and; 13 Department of Intensive Care, IGH, Rourkela, Odisha, India.; 14 Department of Neuroradiology, University Hospital Heidelberg, Heidelberg, Germany.; 15 University Institute of Diagnostic and Interventional Neuroradiology, University Hospital Bern, Inselspital, University of Bern, Switzerland.; 16 European Molecular Biology Laboratory (EMBL), Barcelona, Spain.

**Keywords:** Infectious disease, Microbiology, Malaria, Parasitology, Platelets

## Abstract

Cerebral malaria (CM) affects children and adults, but brain swelling is more severe in children. To investigate features associated with brain swelling in malaria, we performed blood profiling and brain MRI in a cohort of pediatric and adult patients with CM in Rourkela, India, and compared them with an African pediatric CM cohort in Malawi. We determined that higher plasma *Plasmodium falciparum* histidine rich protein 2 (PfHRP2) levels and elevated *var* transcripts that encode for binding to endothelial protein C receptor (EPCR) were linked to CM at both sites. Machine learning models trained on the African pediatric cohort could classify brain swelling in Indian children CM cases but had weaker performance for adult classification, due to overall lower parasite *var* transcript levels in this age group and more severe thrombocytopenia in Rourkela adults. Subgrouping of patients with CM revealed higher parasite biomass linked to severe thrombocytopenia and higher Group A–EPCR *var* transcripts in mild thrombocytopenia. Overall, these findings provide evidence that higher parasite biomass and a subset of Group A–EPCR binding variants are common features in children and adult CM cases, despite age differences in brain swelling.

## Graphical abstract

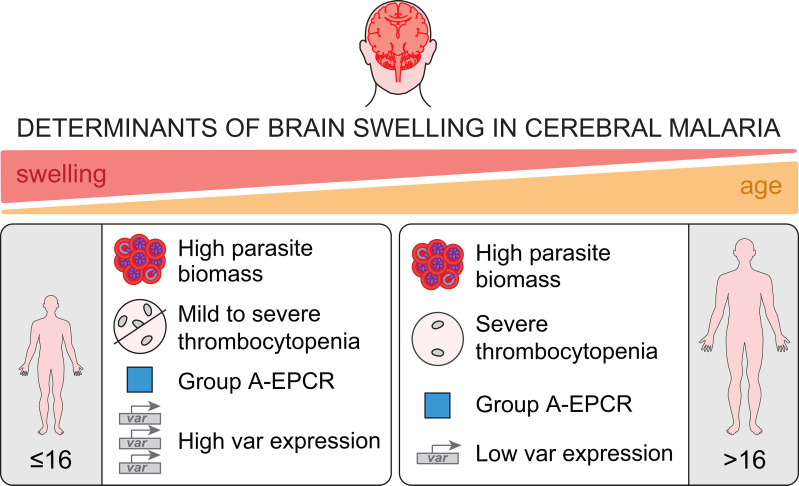

## Introduction

Cerebral malaria (CM) is a severe neurovascular complication of *Plasmodium falciparum* infection characterized by impaired consciousness and seizures, as well as a fatality rate up to 20% even when appropriate clinical management is provided (1). In African regions, where disease transmission is high and immunity develops after repeated infections, severe malaria primarily affects children under the age of 10. By comparison, in lower transmission settings, both children and adults are susceptible, but the clinical manifestations differ across ages (2). In particular, multiorgan complications occur more frequently in adult CM (2, 3), and brain swelling is more severe in children (4). The factors contributing to age differences in CM disease presentation and brain swelling remain unclear.

A hallmark of CM is the sequestration of mature parasitized RBCs (pRBCs) in the cerebral microvasculature (5). However, the extent and pattern of brain histological injury vary by age (3, 6). Brain autopsy studies in African children have revealed microvascular leak, fibrin deposits, and thrombosis associated with sequestered pRBCs (7–10). By comparison, thrombosis is more variable in adult CM, with levels ranging from negligible (6, 8) to high (11). Lumbar punctures and MRI studies indicate that raised intracranial pressure and severe brain swelling are associated with fatal pediatric CM (12, 13). By comparison, brain swelling is milder and not associated with death in adult patients (4, 14). Nevertheless, MRI studies in both children and adults have provided evidence for vasogenic edema resulting from a breakdown of the blood-brain barrier (14–17). Collectively, these autopsy and neuroimaging findings reveal heterogeneity in CM features between children and adults, despite having in common abundant sequestration of pRBCs within the brain microvasculature.

Multiple lines of evidence suggest that sequestered pRBCs might promote a procoagulant state and vascular leak through a variety of processes. For instance, pRBCs induce tissue factor expression on microvascular endothelial cells (18), and parasite products released during pRBC rupture interfere with antithrombin pathways (19, 20) and induce barrier disruption in endothelial cell monolayers (21–23). In addition, parasite binding variants associated with severe malaria may impair localized vascular homeostatic mechanisms (24). Sequestration is mediated by *P*. *falciparum* erythrocyte membrane protein 1 (PfEMP1), a clonally variant antigen encoded by the *var* family (25–27). PfEMP1 proteins contain multiple Duffy binding-like (DBLα/β/γ/δ/ε/ζ) and cysteine-rich interdomain region (CIDRα/β/γ/δ) adhesion domains that sometimes can be found in conserved arrays, known as domain cassettes (DC) (28, 29). A dichotomy exists in the PfEMP1 family between variants that encode binding activity for CD36 (CIDRα2–6 domains) (30, 31), a binding property that is associated with mild infections, or endothelial protein C receptor (EPCR) (CIDRα1 domains) (24, 32), a binding property associated with severe malaria. While EPCR binders are a minor subset in the PfEMP1 repertoire (~12.5% of *var* genes per parasite genotype) (28), this subset is transcriptionally elevated in severe malaria patients (24, 33, 34). Because EPCR plays a key role in vascular homeostasis through activation of protein C, parasite blockade of EPCR function may impair antiinflammatory, anticoagulant, and barrier-protective pathways (33, 35–37).

Given the age-dependent differences in CM presentation and brain swelling, in-depth studies are needed to better understand CM disease etiology. We previously studied a malaria cohort in Blantyre, Malawi, that had undergone MRI brain scans and found that comatose children presented with higher plasma levels of** PfHRP2, a biomarker of parasite biomass (38), and *var* transcripts encoding EPCR binders compared with children with UM (36). In addition, low circulating platelet levels were the best machine learning feature for discriminating comatose children with or without brain swelling (36). Here, to study whether similar factors are linked to CM and brain swelling in different human populations and age groups, we investigated a cohort of both pediatric and adult malaria patients from Rourkela, India, stringently characterized using MRI. By comparing cohorts from Rourkela and Blantyre (36), we provide evidence for similar parasite biomass and adhesion determinants linked to CM and brain swelling in children and adults from Africa and India.

## Results

### Characterization of the study population.

**Figure 1:**
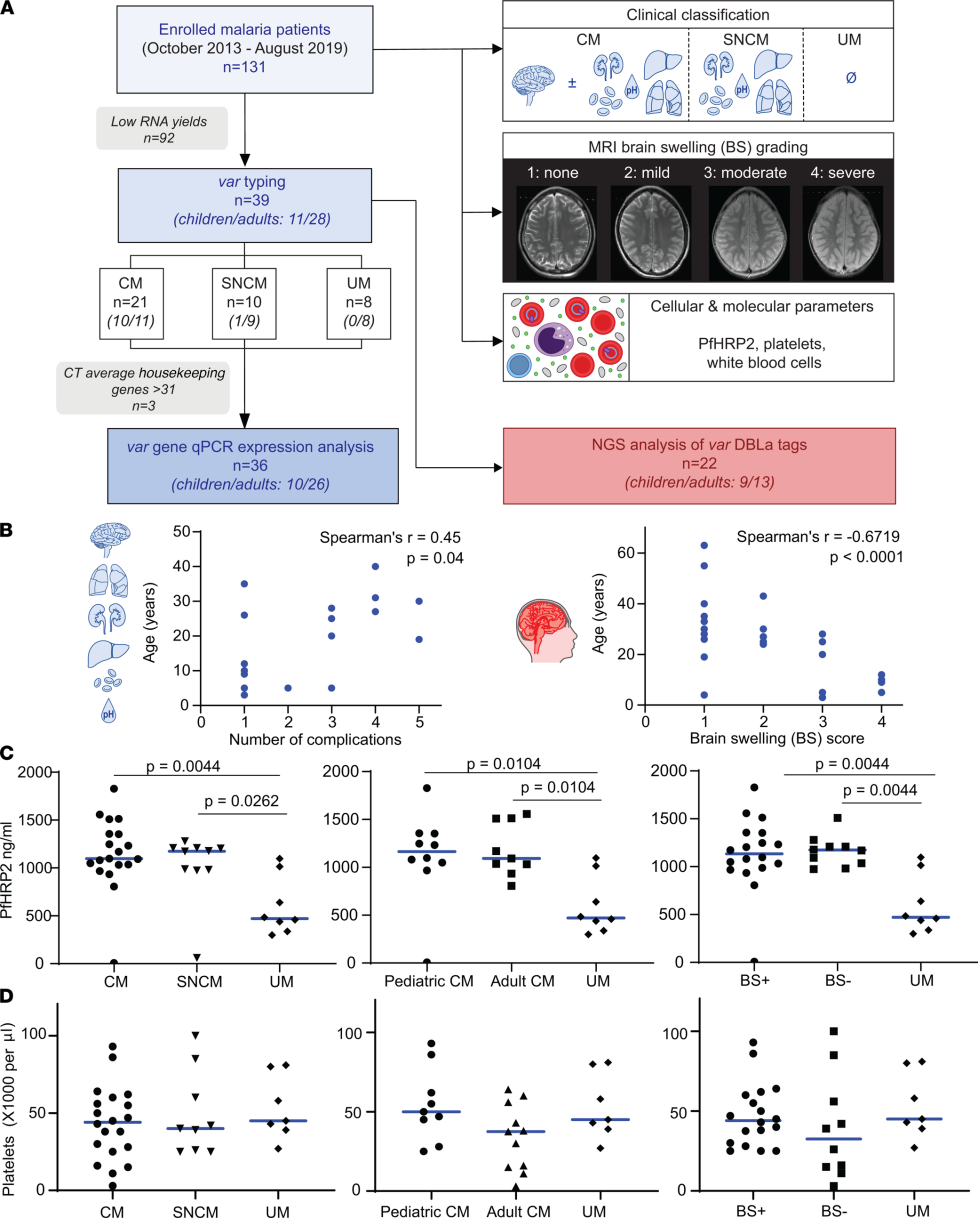
Study consort diagram and population characterization. (**A**) *P*. *falciparum*–positive patients underwent brain MRI scans and subjects that underwent parasite *var* typing were categorized into CM (coma with or without other WHO-severity criteria, *n* = 21, children/adults), SNCM (no coma but other severe complications, *n* = 10, children/adults), and UM (no severe complications, *n* = 8, adults only). MRI-brain scans were scored on a 4-point scale for brain swelling. Patient blood samples were collected to measure plasma PfHRP2 levels (parasite biomass biomarker) and for parasite *var* transcriptional profiling. (**B**) Number of WHO severe malaria complications in patients with CM (left panel). Extent of brain swelling by patient age in patients with CM or SNCM (right panel). Missing dots correspond to overlapping patients. (**C**) Plasma PfHRP2 levels in different patient subsets. Horizontal line represents median. (**D**) Circulating platelet counts in different patient subsets. Horizontal line represents median. Statistical significance in **C** and **D** (*P* < 0.05) was determined by a Kruskal-Wallis test corrected for multiple comparisons by the Benjamini, Krieger, and Yekutieli method. Missing dots in **C** and **D** correspond to data not available. BS+, brain swelling positive; BS–, brain swelling negative.****

A total of 951 patients with *P*. *falciparum*–positive blood smears were enrolled during October 2013 to August 2019 at IGH in Rourkela, India. To evaluate the occurrence of brain swelling in malaria, 131 *P*. *falciparum*–infected patients underwent neuroimaging. Brain-scanned patients ranged in age from 3 to 70 years and had CM with or without additional severity symptoms (*n* = 69), severe noncerebral malaria (SNCM, *n* = 31), or uncomplicated malaria (UM, *n* = 31). Patients with UM were ill enough to be admitted at IGH but did not fulfil the World Health Organization (WHO) criteria for severe malaria. Of these 131 brain-scanned patients, 92 were excluded from the present study due to prior administration of antimalarials and resultant insufficient RNA levels necessary to examine the parasite *var* transcripts. The remaining 39 patients were selected for our analyses (Figure 1A and Supplemental Table 1; supplemental material available online with this article; https://doi.org/10.1172/jci.insight.145823DS1). There were no differences in the clinical and laboratory profiles between the *var*-typed and excluded patients (summarized in Supplemental Table 2), except that excluded patients had lower parasitemia (median, *var*-typed versus excluded patients: 66,880 versus 2,730/μL, *P* = 0.0028) and plasma PfHRP2 levels (median, *var*-typed versus excluded patients: 1080 versus 133.61 ng/mL, *P* < 0.0001). Of the selected patients, 21 had CM (7 with exclusively coma, 14 with coma plus other organ complications), 10 had SNCM, and 8 had UM. The final study population was composed of 11 children (10 CM, 1 SNCM) and 28 adults (11 CM, 9 SNCM, 8 UM). Transcription of *var* genes was analyzed by quantitative PCR (qPCR) in 36 patients and by next-generation sequencing (NGS) in 22 patients (Figure 1A).

Consistent with previous observations (2), the presence of multiorgan complications was positively correlated with age in the Rourkela cohort (*r* = 0.45, *P* = 0.04, Figure 1B). Severe complications included jaundice (48% adults, 18% children), acute kidney injury (29.6% adults, 9.1% children), severe anemia (33% adults, 27% children), and hyperlactatemia (29.6% of adults, 18% of children). Brain swelling on admission was assessed by MRI, using a 1–4 scale (1, no swelling; 2, mild swelling; 3, moderate swelling; and 4, severe brain swelling; Figure 1A). Increased brain volume was negatively associated with patient age, and severe brain swelling was only present in pediatric patients (*r* = –0.67, *P* = 0.0001, Figure 1B). A subset of adult CM cases had mild (27%) or moderate (18%) brain swelling. In addition, 33% of adult patients with SNCM had mild brain swelling.

Plasma levels of PfHRP2, a biomarker of parasite biomass, were elevated in patients with CM and SNCM compared with UM (Figure 1C, left panel), which is in line with previous reports (38, 39). There was no statistically significant difference in PfHRP2 levels between pediatric and adult CM (Figure 1C, middle panel) or patients with brain swelling (BS+) and without brain swelling (BS–) (Figure 1C, right panel). Thrombocytopenia is common in *P*. *falciparum* infection (40). Many patients with CM and SNCM had severe thrombocytopenia (<50,000 platelets/μL), although platelet levels were not significantly different from hospitalized UM cases (Figure 1D, left panel). Platelet counts tended to be lower in adult than pediatric CM cases (Figure 1D, middle panel), but there was no difference in platelet levels in cases with and without brain swelling (Figure 1D, right panel).

### Characterization of var sequence diversity in severe isolates by NGS.

**Figure 2:**
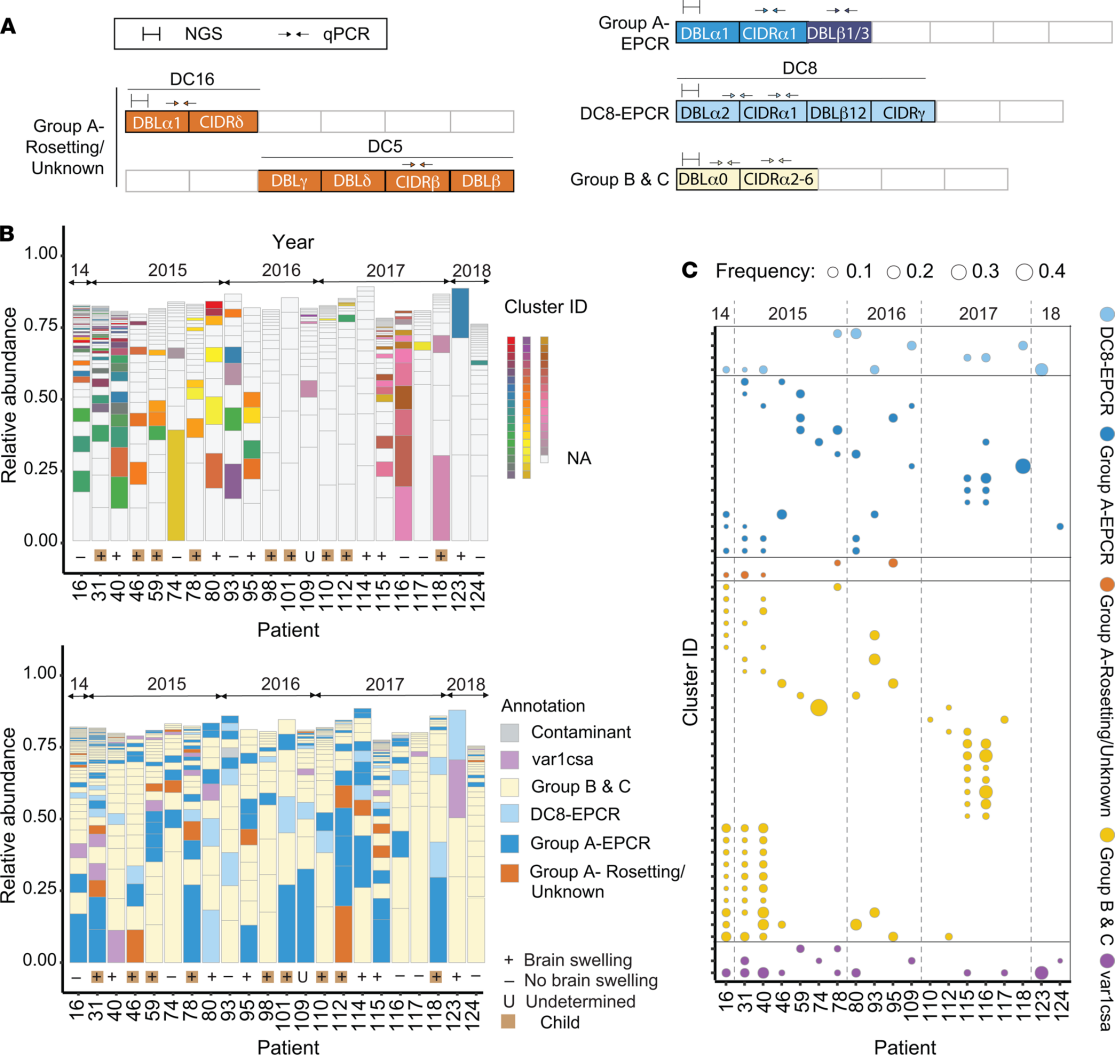
NGS analysis of the var repertoire. (**A**) Schematic representation of PfEMP1/*var* types with color coding of domain and domain cassettes. The location of *var* primers used to amplify the DBLα amplicon and illustrative *var* domain primers are shown. (**B**) Diversity of *var* transcripts in each patient. Top: Proportion of each unique DBLα tag in a patient is indicated by bar size. Identical DBLα tags (≥96% nucleotide identity) shared between patients are indicated by cluster ID color code. Identical var1csa DBLα tags are not colored. Bottom: Functional annotation of DBLα tags in patients. Annotation according to *var* type was done based on BLAST searches of each DBLα tag against a database of 513 annotated *var* culled from refs. 28 and 44. The year of patient enrolment (2014–2018) is shown along the top. Adult and pediatric cases and brain swelling status are indicated below the bar graph. Contaminant: no *var* hits returned. (**C**) Bubble graph showing shared DBLα tags (≥96% nucleotide identity) grouped by *var* type across the study period (2014–2018). The weighted proportion of the tag in a patient is indicated by circle size. Identical var1csa types are shown here. The year of patient enrolment is indicated at the top, with dotted vertical lines demarcating different years.

To date, there has been minimal in-depth characterizations of *var* transcription in adults or low-transmission malaria settings. To investigate the diversity of *var* transcripts associated with severe isolates in the Rourkela cohort, we analyzed *var* transcript profiles in 22 patients by NGS of DBLα amplicons. This short-sequence tag can be amplified from most *var* transcripts by targeting the N-terminal DBLα domain (Figure 2A). This analysis included 19 patients with CM (9 children, 10 adults), 2 with SNCM (2 adults), and 1 with UM (1 adult). Each parasite genotype encodes approximately 60 *var* genes, which are expressed in a mutually exclusive fashion and encode distinct binding properties (41). The NGS analysis showed a complex population of circulating parasites at the time of hospitalization, ranging from 4 to 48 unique DBLα tags per patient (Supplemental Tables 3 and 4), comparable with NGS analysis of the Malawian pediatric CM cohort (36). Overall, there was minimal overlap of parasite variants between Indian patients (Figure 2B). Whereas the majority of patients had no DBLα sequences in common (cut-off ≥ 96% nucleotide identity), a few individuals shared between 1 and 4 DBLα tags (Figure 2B and Supplemental Figures 1 and 2). However, 3 patients from transmission seasons 2014–2015 had a partial overlap of expressed DBLα tags (14–16 tags), and 2 patients from transmission season 2017 had 11 expressed DBLα tags in common (Figure 2B and Supplemental Figure 1). Larger numbers of shared DBLα tags likely represent patients being infected with the same or a closely related circulating parasite genotype. While shared DBLα tags were sometimes present over more than 1 transmission season, none recurred over the full 5-year study period (Figure 2C).

The *var* gene family is classified into Group A, Group B, and Group C by 5′ upstream sequence and chromosomal location (42) and has diverged in PfEMP1 binding properties (43). Of these, a subset of Group A variants and an unusual chimeric B/A variant called DC8 encode EPCR-binding CIDRα1 domains. Here, we will refer to these as Group A–EPCR and DC8-EPCR (Figure 2A). A distinct subset of Group A *var* genes has been associated with rosetting with uninfected RBCs or unknown binding properties and is termed Group A–rosetting/unknown. Group B and Group C are associated with CD36 binding. To classify DBLα tags, we performed a BLAST search against a library of 513 *var* genes (Supplemental Table 4) (28, 44). In phylogenetic analysis, DBLα tag sequences clustered according to *var* type — and not according to age group or swelling status (Supplemental Figure 2). Notably, despite being a rarer PfEMP1 subset, the most abundant DBLα tags belong to Group A–EPCR or DC8-EPCR in 9 of 22 patients (41%) (Figure 2B). However, the dominant tags were usually not shared between different patients (compare upper and lower panels, Figure 2B). While the top transcript from patient 118 was a Group A–EPCR and shared with 1 other CM patient, the majority of shared DBLα tags belonged to Group B and Group C and were not the most abundant transcript per patient (Figure 2, B and C). Altogether, the NGS findings indicate that a broad diversity of *var* variants is linked to CM in the Rourkela cohort.

### Analysis of var typing across ages in CM and brain swelling patients.

**Figure 3:**
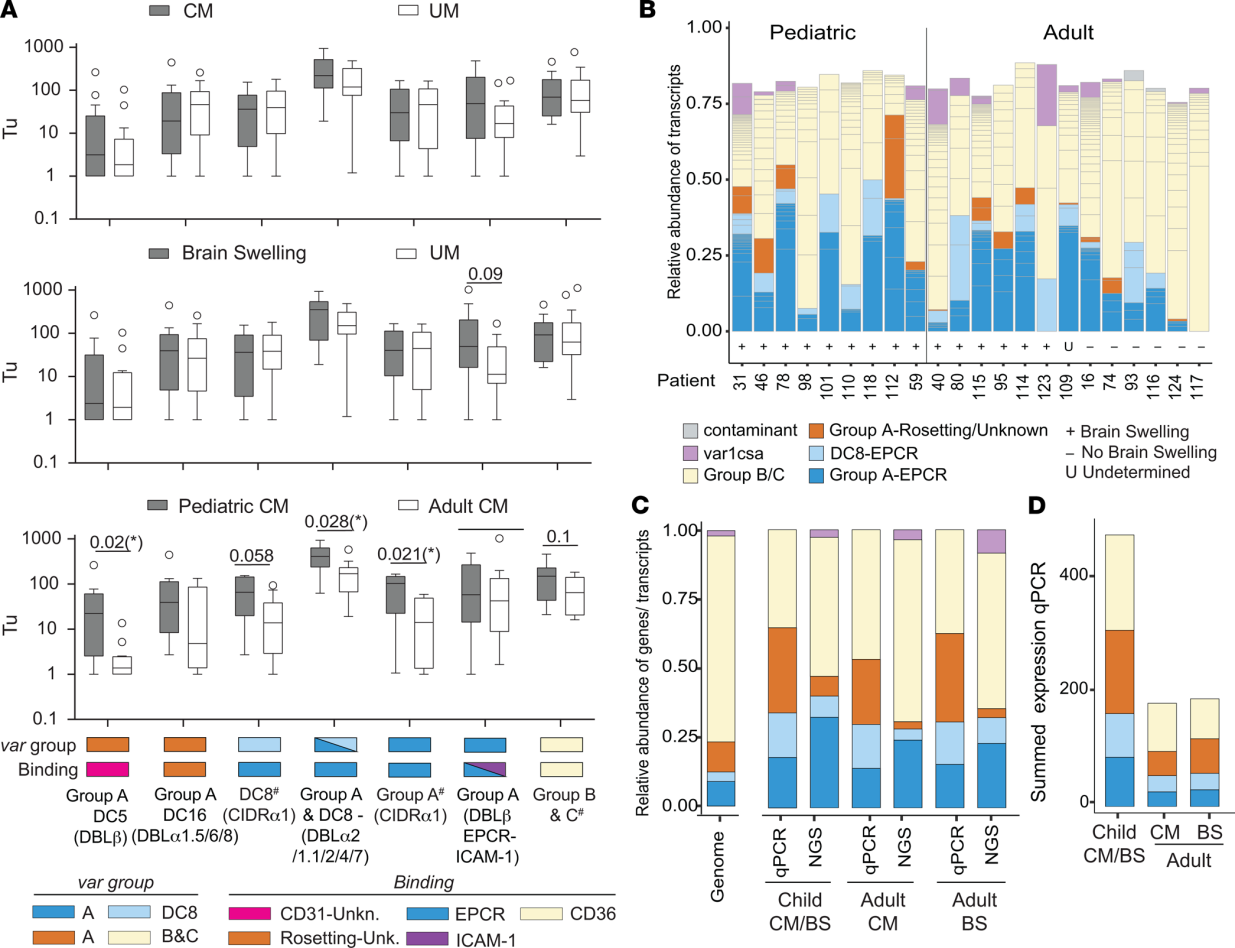
Characterization of parasite var transcript profiles by qPCR analysis. (**A**) Tukey box and whisker plots showing *var* domain transcript expression in CM (*n* = 19) and UM (*n* = 8) patients (top), brain swelling patients (BS^+^, score > 1, *n* = 16) (middle), as well as pediatric (*n* = 9) and adult (*n* = 12) CM and/or BS cases. Individual or summed primer groups (#) are shown: DC8^#^ (CIDRα1) = CIDRα1.1, CIDRα1.8a, and CIDRα1.8b (*n* = 3); Group A^#^ (CIDRα1) = CIDRα1.4/6, CIDRα1.5a, CIDRα1.5b, CIDRα1.6, CIDRα1.7 (*n* = 5); Group B/C^#^ = DBLα0.1, DBLα0.6/9, CIDRα2/3/5/6/7/9/10, CIDRα2.2, and DBLα0.16 (*n* = 5). The horizontal line is the median, and boxes indicate quartiles. Outliers are indicated as circles. Significance was determined by Mann-Whitney *U* test. *P < 0.05. (**B**) Stacked bar graph showing functional annotation of DBLα tags in pediatric and adult patients measured by NGS. See also Figure 2. (**C**) Left: Proportion of *var* subgroup abundance in 7 annotated reference genomes (28). Right: Proportional expression of *var* subgroups measured by qPCR and NGS across patient subgroups in CM^BS+^ (*n* = 8), adult CM (*n* = 8), adult BS^+^ (*n* = 5). (**D**) Average of the summed *var* transcript levels measured by qPCR (*var* Tu) for each *var* subtype across patients in (**C**).

To complement the NGS analysis of *var* transcription, we performed qPCR analysis on 36 patients with 24 domain-specific primers targeting transcripts encoding Group B and Group C (CD36 binders) (34), DC8-EPCR, Group A–EPCR, or Group A–rosetting/unknown (34, 45) (Supplemental Table 5). Compared with patients with UM, patients with CM and brain swelling cases had higher transcript levels of DC5 (Group A, linked to CD31/PECAM-1 binding) (46), a primer targeting both DC8-EPCR and Group A–EPCR (DBLα2/α1.1/2/4/7), and a Group A subset with dual binding to EPCR–ICAM-1 (47) (Figure 3A). However, none of these comparisons reached statistical significance compared with the hospitalized UM group (Figure 3A, top and middle panels).

Overall, there was high concordance between the qPCR and the NGS analysis (Figure 3, A–C). Whereas individual *var* transcript levels did not differ between UM and CM cases by *var* domain typing (Figure 3A), both methodologies reported approximately 3 times higher proportions of Group A–EPCR and DC8-EPCR transcripts in patients with CM compared with the genome representation (~30% of *var* transcripts in patients with CM versus ~12.5% of PfEMP1 repertoire) (Figure 3, B and C) (28). Notably, the summed transcript levels of all *var* subsets were 3-fold higher in children compared with adults, after correction for parasitemia using transcript levels of a housekeeping gene (Figure 3D). All categories of detected *var* transcripts were elevated in pediatric cases (groups A, B, and C), with differences reaching significance in Group A–EPCR transcripts, Group A and DC8-EPCR transcripts (DBLα2/α1.1/2/4/7),and Group A–DC5 (Figure 3A, bottom). Taken together, our analysis shows an elevated proportion of EPCR-binding PfEMP1 transcripts in both children and adults with CM, and the total transcript levels of all *var* subsets were higher in children.

### Machine learning models of pediatric CM, adult CM, and brain swelling.

**Figure 4:**
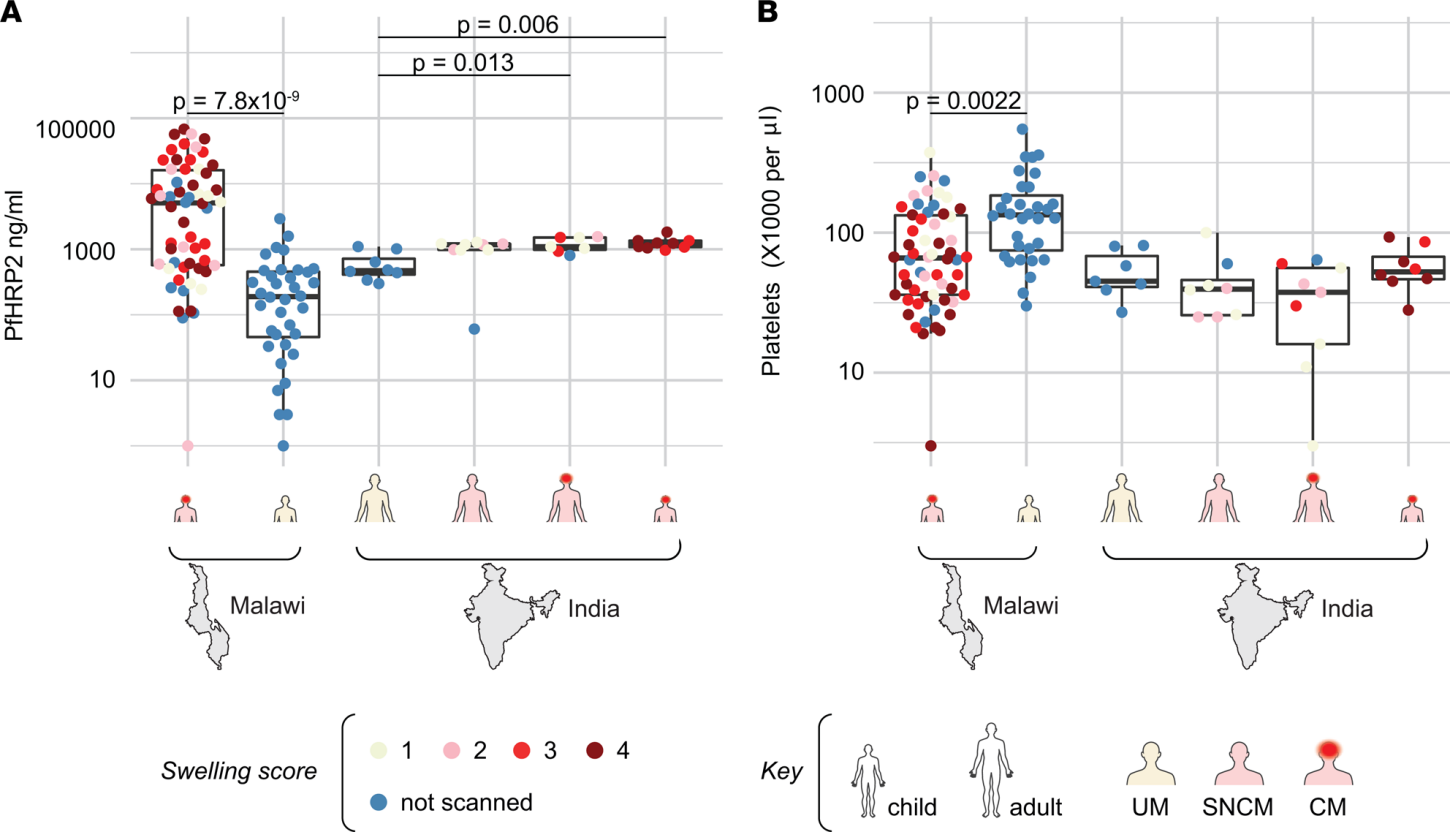
Measurement of plasma PfHRP2 levels and circulating platelet counts in the Malawi and India cohorts. (**A**) Plasma PfHRP2 concentration across patient groups in Malawi (pediatric CM [*n* = 58], pediatric UM [*n* = 36]) and India (adult UM [*n* = 8], adult SNCM [*n* = 9], adult CM [*n* = 7], pediatric CM [*n* = 9]). Horizontal line represents median, and boxes represent interquartile range. Significance was determined by FDR-adjusted Wilcoxon *P* values. (**B**) Circulating platelet counts across patient groups in Malawi (pediatric CM [*n* = 58], pediatric UM [*n* = 32]) and India (adult UM [*n* = 7], adult SNCM [*n* = 8], adult CM [*n* = 9], pediatric CM [*n* = 8]). Horizontal line represents median, and boxes represent interquartile range. Significance was determined by FDR-adjusted Wilcoxon *P* values. Swelling score is indicated with a color scale, and age group and severity are indicated with schematic diagrams.

To investigate determinants associated with CM and brain swelling in different human populations and transmission settings, we combined data sets from the Rourkela cohort (21 CM, 8 UM) and our previously published Blantyre pediatric CM cohort from Malawi (57 CM, 38 UM) (36). Patients in both locations underwent brain swelling assessment through MRI and were quantified for plasma PfHRP2 levels using the same kit, and their PfEMP1 transcripts were analyzed using a common subset of 19 *var* domain primers (36). This comparison showed that the adult UM control group in Rourkela had higher PfHRP2 levels and lower platelet counts (both indicators of more severe illness) than the pediatric UM control group in Blantyre, who did not necessitate hospitalization (Figure 4). The finding likely explains why the Rourkela UM cases had higher EPCR binding *var* transcripts and were less distinguishable from CM cases (Figure 3B), similar to our previous findings in a different adult Indian cohort in Goa (33).

To study the combined cohorts, we generated random forest (RF) models using PfHRP2 levels, platelet counts, and the 19 *var* domain primers as features. As shown by unsupervised hierarchical clustering, the 19 *var* domains targeted by qPCR show multiple correlations with each other, because some of the primer sets target different domains within the same protein, or specific PfEMP1 variants tend to be expressed together (Supplemental Figure 3) (48). An advantage of RF modeling is that this approach mitigates the impact of multicollinearity associated with *var* transcription. RF models were trained on different patient subsets. In some RF models, patients with UM were used as the control outgroup to compare between patients who differed the most in disease spectrum (CM versus UM). Other RF models focused on the subpopulation of comatose patients to study whether specific features were more closely associated with brain swelling (CM^BS4–2^ [brain swelling score = 2–4] versus CM^BS1^). RF models trained with either PfHRP2 or *var* transcripts alone performed well on unbiased out-of-bag predictions measured by receiver operating characteristic (ROC) curve and 95% CIs (Supplemental Figure 4). On their own, PfHRP2 levels and *var* transcripts (19 *var* domain features) discriminated patients with CM from patients with UM in the combined (Supplemental Figure 4) or individual cohorts (Supplemental Figure 5). By comparison, neither was able to classify the presence of brain swelling within patients with CM (CM^BS+^) (Supplemental Figure 4), indicating that high parasite biomass and specific parasite adhesion types were common to patients with CM at both sites, but these features did not discriminate the presence of brain swelling. Whereas platelet count was an important feature for predicting the occurrence of severe brain swelling in children with CM from Blantyre, it performed poorly in the Rourkela cohort or the combined data set (Supplemental Figure 5). This is likely due to the high prevalence of severe thrombocytopenia in adult cases from Rourkela, irrespective of CM status and brain swelling (Figure 1D and Figure 4B).

**Figure 5:**
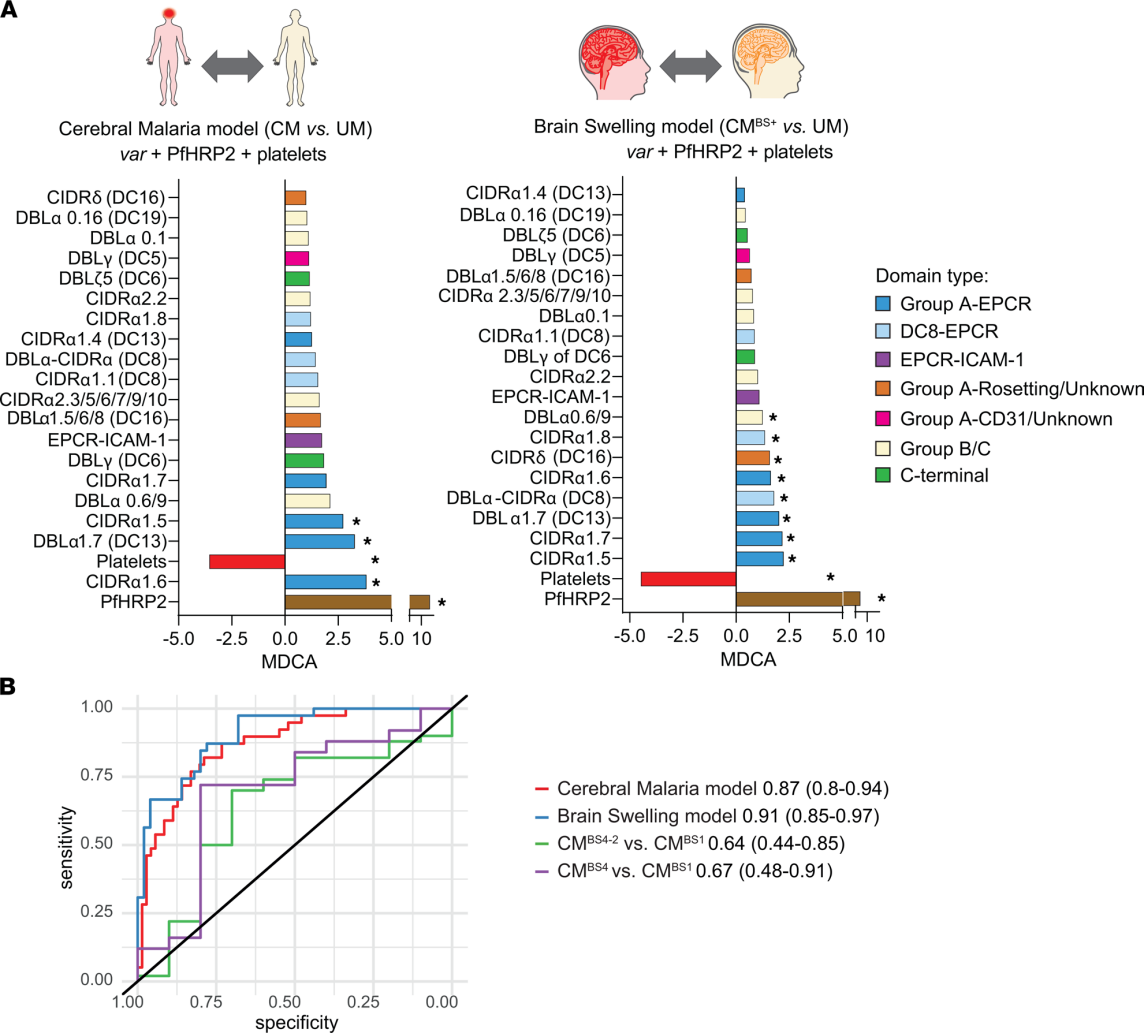
Machine learning analysis of CM and brain swelling in the combined India and Malawi cohorts. (**A**) Random forest models were trained on different patient subsets: CM versus UM (left) and brain swelling versus UM (right) that combine patients from Rourkela and Blantyre. Each individual bar represents the predictive performance of the feature in the model ordered by predictive importance (measured by MDCA). Features include: circulating platelet levels (platelets), plasma PfHRP2 concentration (PfHRP2), and *var* domain transcriptional units. Positive MDCA scores indicate higher presence of a feature in CM or patients with brain swelling, and vice versa. Asterisks indicate that these features showed significant difference between groups measured by mProbes algorithm (family-wise error rate [FWER] ≤ 0.2). (**B**) Receiver operating characteristic (ROC) curves of CM model (CM versus UM), CM with brain swelling model (CM^BS+^ versus UM), brain swelling within patients with CM (CM^BS2–4^ versus CM^BS1^), and severe brain swelling within patients with CM (CM^BS4^ versus CM^BS1^). Area under the ROC curve (ROC AUC) and accompanying 95% CI in parentheses are indicated in the ROC plot legend for each model.

A combination of parasite biomass, *var* expression profiles (19 *var* domain features), and platelet levels had the strongest predictive performance in CM versus UM models (Figure 5 and Supplemental Figure 4). The top-ranking features in the CM model (CM versus UM: AUC, 0.87 [95% CI, 0.80–0.94]) were high PfHRP2 levels and high expression of CIDRα1.6 Group A–EPCR *var* transcript, as determined by the mean decrease in classifier accuracy (MDCA) when the feature in question was removed from the model (Figure 5A, left panel). The third ranked feature was platelet counts, followed by Group A–EPCR *var* transcripts (DBLα1.7 of DC13). The significance of each feature and multiple-comparison correction was determined by the mProbes algorithm (49). Likewise, in an RF model that focused on the subset of comatose patients with brain swelling (CM^BS+^ versus UM: AUC, 0.91 [95% CI, 0.85–0.97]), both high PfHRP2 and low peripheral platelet counts were the top predictive features (Figure 5, A and B). In addition, transcripts encoding Group A–EPCR variants (CIDRα1.5, CIDRα1.6, CIDRα1.7, and DBLα1.7 of DC13) and DC8-EPCR *var* transcripts (DBLα-CIDRα of DC8 and CIDRα1.8) were statistically significantly increased in the model comparing CM^BS+^ with UM. Overall, high parasite biomass, multiple subsets of Group A–EPCR, and low platelet counts were key determinants of CM and brain swelling in the RF model comparisons to UM cases.

By comparison, the same 3 features were unable to distinguish the presence or absence of brain swelling in patients with CM (CM^BS4–2^ versus CM^BS1^: AUC, 0.64 [95% CI, 0.44–0.85]; CM^BS4^ versus CM^BS1^: AUC, 0.67 [95% CI, 0.48–0.91]) (Figure 5B and Supplemental Figure 4). The addition of WBC count and lactate improved the predictive performance of an RF model comparing the 2 extremes of brain swelling in patients with CM (CM^BS4^ versus CM^BS1^: AUC, 0.73 [95% CI, 0.54–0.92]). In this RF model, patients with CM with severe brain swelling were distinguished from patients with CM with no brain swelling by higher transcription levels of a Group A–rosetting/unknown variant (CIDRδ of DC16) and a Group A–EPCR binding variant (CIDRα1.7), as well as lower platelet counts, lower lactate concentrations, and lower transcription levels of a Group B/C *var* gene (DC19) (Supplemental Figure 4B). However, none of these top-ranked features had statistical significance on their own in the RF model after correction for multiple comparisons. In general, the RF models performed similarly in the combined cohorts and within the Malawi cohort (Supplemental Figure 5), reinforcing the importance of high PfHRP2 levels and Group A–EPCR *var* transcripts in all patients with CM.

**Figure 6:**
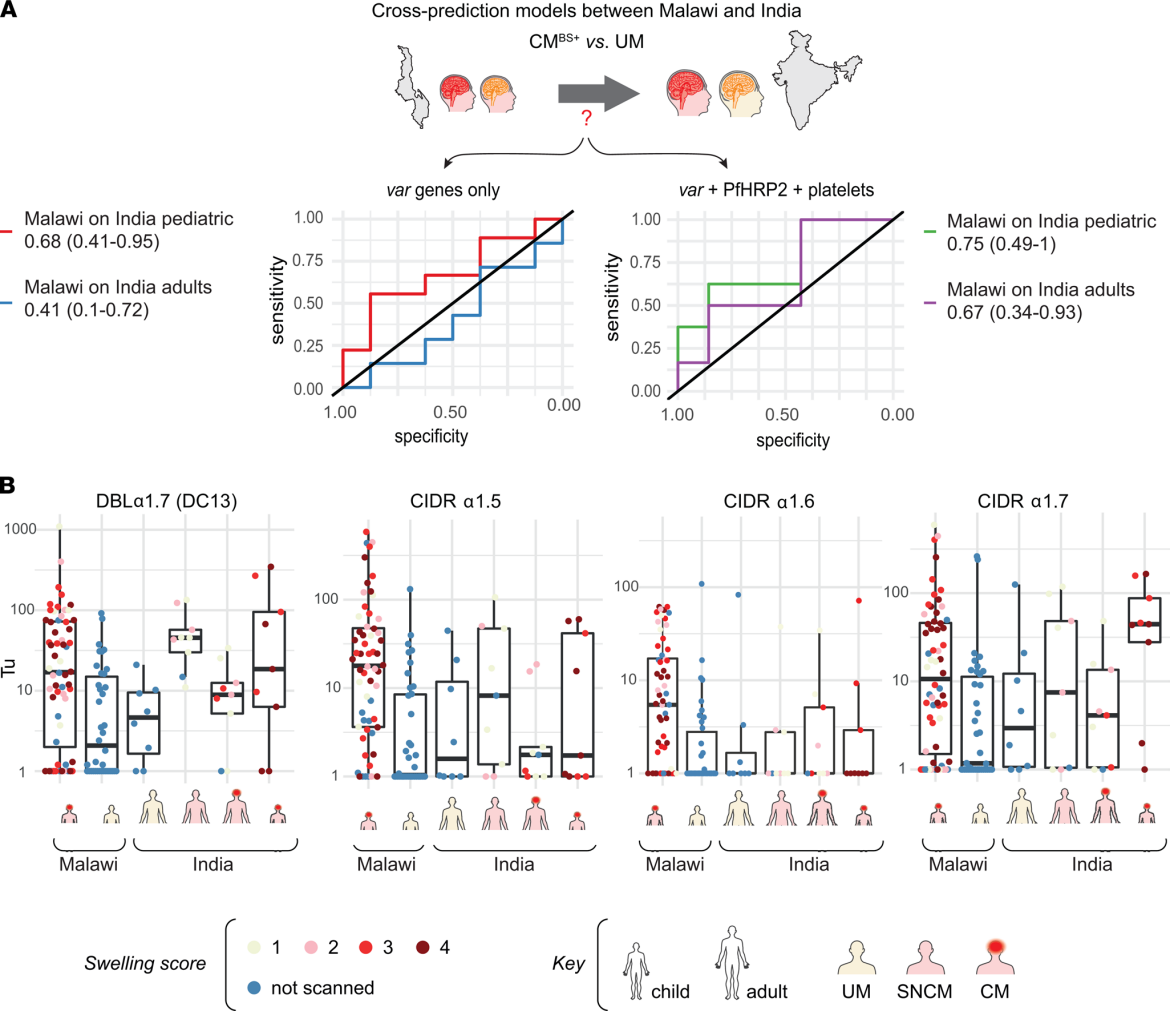
Cross-prediction of patients with CM in India from RF models trained on Malawi patients. (**A**) RF models were trained and parameterized using Malawi data sets only with *var* expression data alone or *var* in combination with PfHRP2 and platelet levels prior to predicting on Rourkela patients. ROC curves illustrating blind predictive performance of random forest models that classify brain swelling patients (CM^BS+^ versus UM) stratified by pediatric and adult patients in Rourkela. ROC AUC and accompanying 95% CI in parentheses are indicated in the ROC plot legend for each model. (**B**) Transcripts levels of Group A–EPCR *var* transcripts (DBLα1.7 of DC13, CIDRα1.5, CIDRα1.6, CIDRα1.7) in UM, SNCM, and patients with CM from Malawi (pediatric CM [*n* = 60], pediatric UM [*n* = 38]) and India (adult UM [*n* = 8], adult SNCM [*n* = 9], adult CM [*n* = 9], pediatric CM [*n* = 9]). Horizontal lines represent median, and boxes represent interquartile range.

To investigate age differences in *var* gene expression in brain swelling, RF models were trained on the pediatric cohort from Blantyre (CM versus UM) and then used to make blind predictions of adult and pediatric patients with CM in Rourkela (Figure 6A). An RF *var-*only model trained in Blantyre failed to discriminate children with brain swelling from UM cases (AUC, 0.68 [95% CI, 0.41–0.95) and performed poorly on adult patients with CM in India (AUC, 0.41 [95% CI, 0.1–0.72]). However, the addition of PfHRP2 levels and platelet counts improved the predictive performance of the models that predicted adult (AUC, 0.67 [95% CI, 0.34–0.93]) and pediatric (AUC, 0.75 [95% CI, 0.49–1]) CM with brain swelling in India (Figure 6A). The poorer performance in adults is likely due to decreased *var* transcript levels and lower platelet counts in adult CM cases (Figure 3, A and D). To further clarify the relationship of Group A–EPCR *var* transcripts in children and adult patients with CM, *var* transcript levels were plotted in the 2 cohorts. There are 4 types of Group A–EPCR subsets (DBLα1.7-CIDRα1.4 of DC13, CIDRα1.5, CIDRα1.6, and CIDRα1.7) assessed by our primer sets. Whereas all 4 Group A–EPCR binding *var* transcripts were elevated in Malawi CM cases, the DBLα1.7 (DC13) and CIDRα1.7 were more commonly elevated in the India cohort (Figure 6B). Thus, a subset of Group A–EPCR *var* transcripts was elevated in both children and adult CM cases, especially compared with children UM cases in Malawi. Overall, these findings indicate that some Group A–EPCR *var* transcripts and parasite biomass are elevated in CM in both children and adults.

### Machine learning models of patients with CM with mild or severe thrombocytopenia.

**Figure 7:**
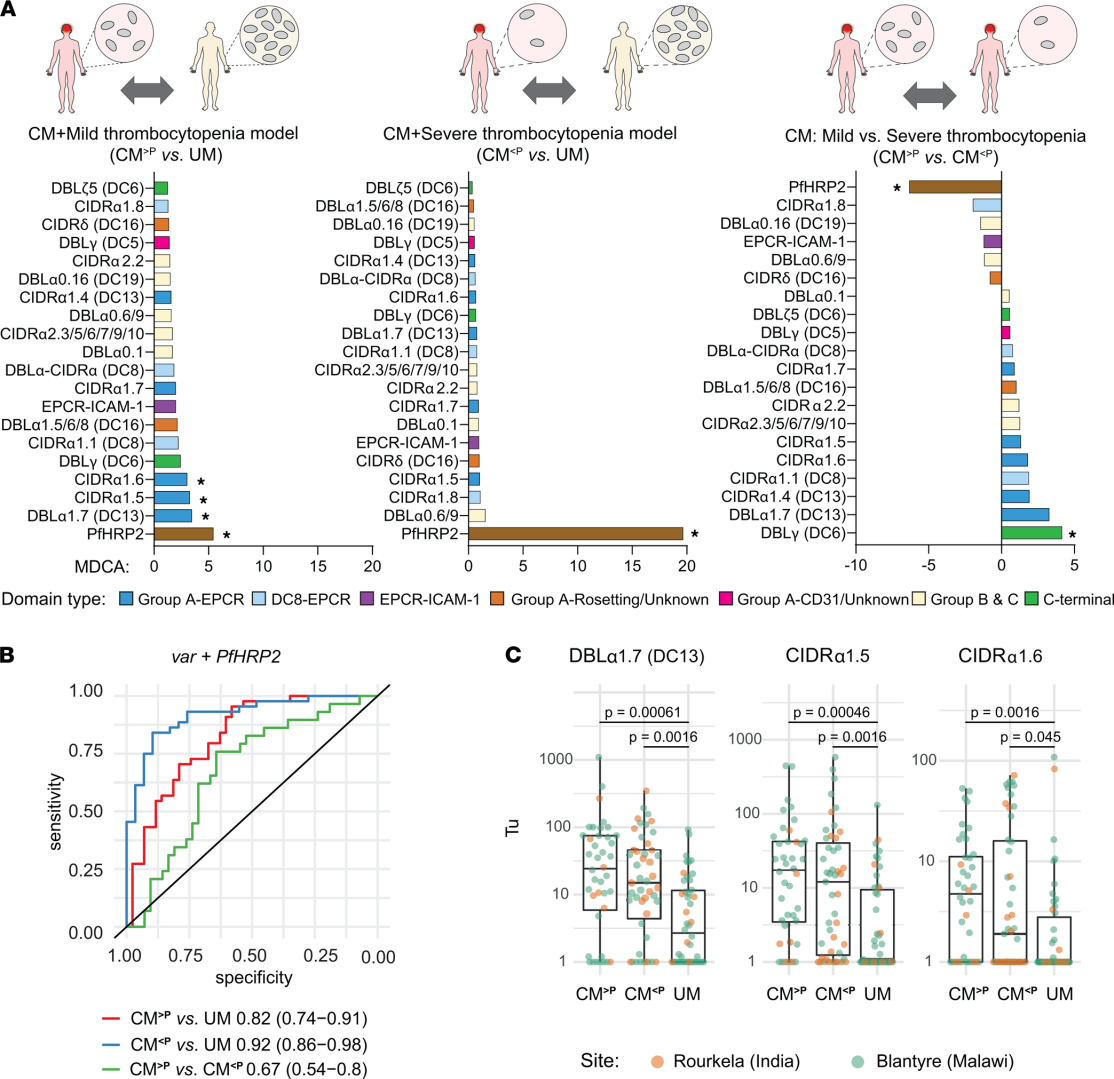
Machine learning models and individual contribution of parasite biomass and Group A–EPCR variants in patients with CM with mild or severe thrombocytopenia in Malawi and India. (**A**) Bar graphs representing the importance of parasite factors in random forest models that classify patients with CM with different degrees of thrombocytopenia (mild > 50,000 platelets/μL; severe < 50,000 platelets/μL). Left: Patients with CM with mild thrombocytopenia versus UM. Middle: CM with severe thrombocytopenia versus UM. Positive MDCA (left and middle graphs) indicate greater presence in CM than in patients with UM. Asterisks show significant difference between groups measured by mProbes algorithm ([FWER] ≤ 0.2). Right: Patients with CM with mild thrombocytopenia versus CM with severe thrombocytopenia. Positive MDCA indicates higher association in CM with mild thrombocytopenia, and negative MDCA indicates higher association in CM with severe thrombocytopenia. Features include: plasma PfHRP2 concentration (PfHRP2), and *var* domain transcriptional units. (**B**) ROC curves showing the predictive performance of each model. Performance is measured by AUC, indicated in the legend with 95% CI in parentheses. (**C**) Transcripts levels of Group A–EPCR (DBLα1.7 [DC13], CIDRα1.5 and CIDRα1.6) in patients with CM with mild thrombocytopenia (CM^>P^; peripheral platelets > 50,000/μL; Malawi *n* = 33, India *n* = 7), CM with severe thrombocytopenia (CM^<P^; peripheral platelets < 50,000/μL; Malawi *n* = 27, India *n* = 20), and combined UM from India and Malawi (Malawi *n* = 38, India *n* = 8). Horizontal lines represent median, and boxes represent interquartile range. Significance was determined by FDR-adjusted Wilcoxon *P* values.

Although platelet counts ranked as an important feature for classifying CM and brain swelling in all models (Supplemental Figure 5, right), the extent of platelet loss varied between CM cases. Because nearly all CM cases from both cohorts had higher parasite biomass (PfHRP2 levels > 1000 ng/mL (Supplemental Figure 6, A and B), this suggests that brain swelling can occur in the presence of mild or severe thrombocytopenia. To further investigate this finding, patients with CM from the combined data set were classified into mild thrombocytopenia (CM^>P^ [peripheral platelets > 50,000/μL]) and severe thrombocytopenia (CM^<P^ [peripheral platelets < 50,000/μL]), and RF models were trained to discriminate these groups from patients with UM. Whereas patients with CM with mild thrombocytopenia were characterized by moderately high PfHRP2 levels and elevated levels of multiple Group A–EPCR *var* transcripts (CIDRα1.5, CIDRα1.6, and DBLα1.7-DC13) (AUC, 0.82 [95% CI, 0.74–0.91]) (Figure 7A, left panel; Figure 7B), only extremely high PfHRP2 levels discriminated patients with CM with severe thrombocytopenia from patients with UM (AUC, 0.92 [95% CI, 0.86–0.98]) (Figure 7A, middle panel; Figure 7B). The same differences in parasite biomass and adhesion types were observed when the mild and severe thrombocytopenia CM groups were compared in an RF model (AUC, 0.67 [95% CI, 0.54–0.80]) (Figure 7A, right panel; Figure 7B). Consistent with RF modeling, Group A–EPCR binding *var* transcripts were elevated in both the mild and severe thrombocytopenia groups compared with UM cases, although they tended to be expressed at higher levels in the mild thrombocytopenia group, although there is no statistically significant difference (Figure 7C). In addition, platelet counts were inversely correlated to plasma PfHRP2 levels at both sites (children: ρ = –0.66, *P* = 3.1 × 10^–13^; adults: ρ = –0.53, *P* = 0.013)(Supplemental Figure 6, A and B), accounting for the stronger linkage between parasite biomass and severe thrombocytopenia.

Likewise, in an RF model that specifically focused on the subset of patients with CM with brain swelling, all 4 Group A–EPCR *var* transcripts (DBLα1.7-DC13, CIDRα1.5, CIDRα1.6, and CIDRα1.7) and a primer recognizing Group A dual EPCR and ICAM-1–binding parasites strongly discriminated brain swelling patients with mild thrombocytopenia from patients with UM (AUC, 0.91 [95% CI, 0.88–0.98]). However, PfHRP2 was the most discriminating feature between severe thrombocytopenic and patients with UM (AUC, 0.91 [95% CI, 0.83–0.98]) (Supplemental Figure 6, B–D). Taken together, our findings indicate that patients with CM with mild or severe thrombocytopenia were distinguished by parasite biomass and Group A–EPCR binding *var* transcripts from patients with UM, although both features were elevated in all patients with CM compared with UM cases.

## Discussion

Studies to understand the molecular determinants of CM have mostly focused on African pediatric populations (50). However, disease presentation differs by age, so it is important to study both children and adults. In this work, we investigated molecular mechanisms linked to brain swelling in CM across age groups and transmission settings by comparing patients from India, where CM occurs in all age groups, and Malawi, where CM is predominantly a pediatric disease. These 2 cohorts are among the few in the world to undergo MRI scanning for rigorous brain swelling evaluation in CM, allowing the unique comparative analysis presented here.

Previous findings have established that parasite biomass (51, 52), and EPCR-binding *var* transcripts (24, 34, 36, 45, 53, 54) play an important role in pediatric CM. However, much less is known about how parasite adhesion traits influence disease presentation in adults and non-African populations. To reduce confounding variables when comparing between cohorts, similar approaches were used to assess parasite biomass (plasma PfHRP2 levels) and adhesion types at both sites. Regarding the control groups in the 2 cohorts, adult patients with UM in India had higher PfHRP2 levels and lower platelet counts than children UM cases in Malawi, indicating they were on the sicker end of the malaria disease spectrum than the Blantyre cohort. The difference is likely because all of the adult UM cases in Rourkela required hospitalization, even though they were classified as UM based on the absence of WHO severe malaria criteria. By comparison, the control group in Malawi was recruited from the outpatient ward. The observation that the Rourkela control group was sicker may account for some differences when comparing within and between the 2 cohorts. While plasma PfHRP2 levels distinguished Indian patients with WHO severity criteria, consistent with the finding in other work that parasite biomass is linked to disease severity (38), this plasma biomarker did not differ between comatose children and adults in India, suggesting that age-specific differences in brain swelling are not merely a consequence of different parasite burdens. To study the *var* adhesion types linked to CM and brain swelling, we performed NGS of DBLα amplicons and transcriptional profiling with *var* domain–specific primers. Both methodologies detected an increase in the proportion of EPCR-binding *var* transcripts compared with their genome abundance. Deep sequencing of parasite *var* tags showed a broad diversity of EPCR-binding *var* sequences in Indian patients with CM, even though it is a low malaria transmission setting. By *var* domain typing, all 4 types of Group A–EPCR subsets (CIDRα1.4/1.5/1.6/1.7) were top *var* features in CM models from the combined cohorts. Whereas all 4 Group A–EPCR subsets were transcriptionally elevated in Malawi children CM cases, some were less common in the Rourkela cohort. The DBLα1.7-DC13 subset and the CIDRα1.7 subset, which was previously linked to severe brain swelling and fatality in Malawi (36), were frequent in Rourkela CM cases. Overall, our analysis implicates multiple subsets of Group A–EPCR binding transcripts in CM, although there may be age and geographical variation that remain to be investigated. Additionally, DC8-EPCR binding *var* transcripts were top features in some RF models that focused on subpopulations of CM patients that differed in the extent of brain swelling or thrombocytopenia. Previous findings suggest that both Group A–EPCR and DC8-EPCR variants can inhibit the homeostatic and probarrier EPCR-APC pathway in the cerebral vasculature (33, 35, 55, 56). Taken together, our results strongly reinforce the importance of parasite biomass and elevated EPCR-binding *var* transcripts in CM pathogenesis.

**Figure 8:**
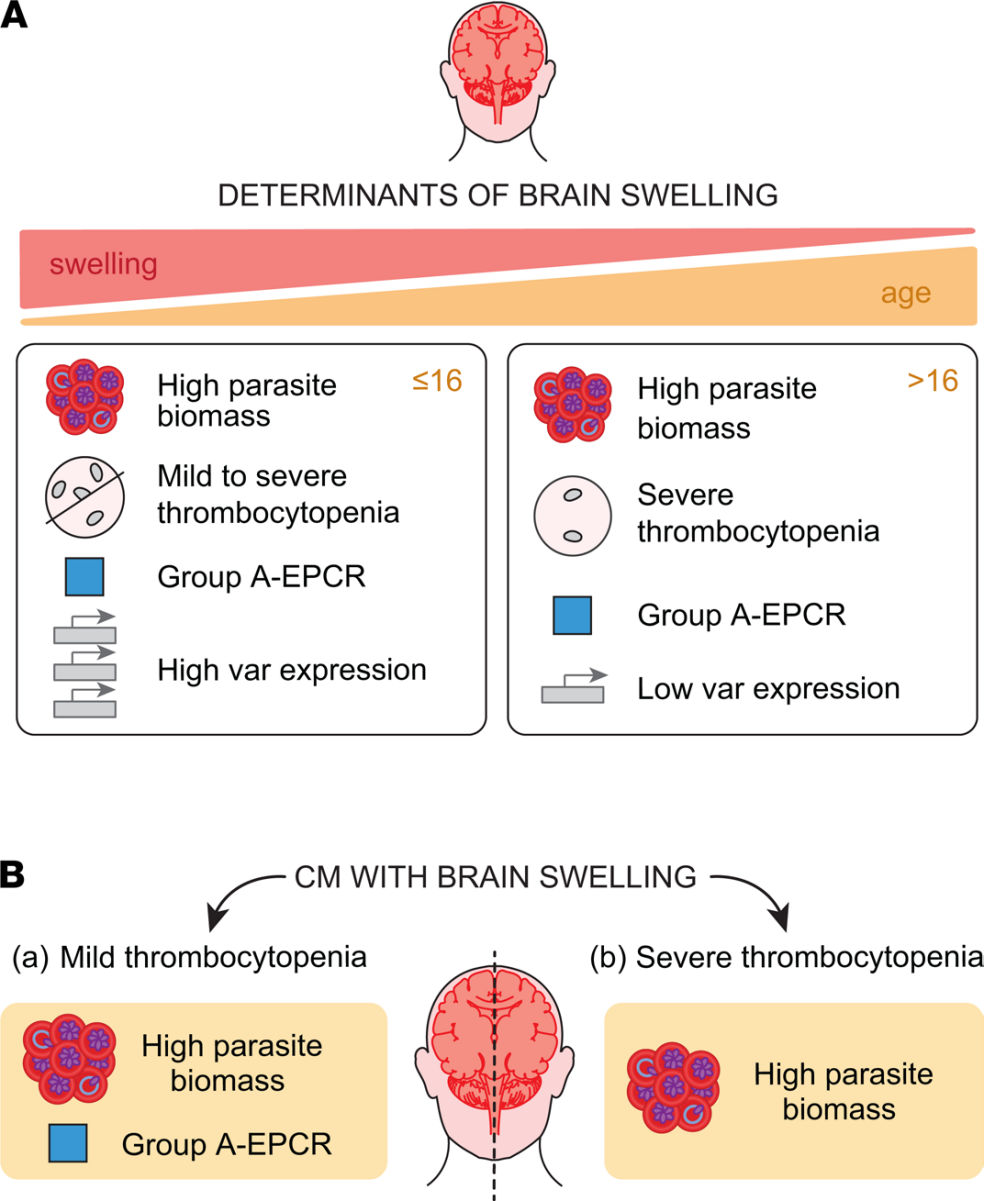
Illustrated summary of the main study findings. (**A**) Features associated with brain swelling in children and adults. (**B**) Features associated with brain swelling in patients with CM with mild or severe thrombocytopenia.

The major age-specific differences seen in this study were substantially lower parasite *var* transcript levels and more severe thrombocytopenia in adult CM cases (summarized in the Figure 8 model). It remains to be established whether lower *var* transcription translates into reduced PfEMP1 surface expression. However, it has been hypothesized that parasites can switch between low or high PfEMP1 expression states to avoid immune detection (57). Recent evidence suggests that factors such as febrile temperature and lactate levels may modify PfEMP1 surface display or transcripts involved in forming the cytoadhesion complex (58–62). Therefore, it is interesting to speculate that differences in *var* transcript levels could relate to age-specific differences in the host response, but this remains to be investigated (59, 60, 62).

Our study suggests there may be interactions between platelets, parasite biomass, and specific parasite variants in CM disease etiology (modelled in Figure 8B). Whereas Group A–EPCR-binding *var* transcripts were more prominent in the mild thrombocytopenia patient subset, patients with severe thrombocytopenia had higher parasite biomass. Thus, parasite biomass and parasite adhesion types may make different contributions to disease progression in patients with CM with mild versus severe thrombocytopenia. Along with brain histological findings (6–11, 63), our findings add to accumulating evidence for different causal pathways of endothelial dysfunction and brain swelling in CM. Activated platelets have long been suspected to play an important role in the pathogenesis of CM: they accumulate in the cerebral microvasculature in fatal pediatric CM cases (64), can bind to endothelial cells and provide receptors for PfEMP1 initially absent from microvascular beds (65), form clumps with pRBCs (66, 67), and — through these 3 mechanisms — may aggravate microvascular obstruction (68). Conversely, platelets can also directly inhibit parasite growth (69, 70), and malaria-associated thrombocytopenia is partly a consequence of the protective mechanism of platelet adhesion to pRBC (71, 72). Given the difference in brain-sequestered platelets in children and adult CM cases (6), platelets could be a causative agent of CM pathology or a biomarker for other inflammatory processes. There are numerous interactions between platelets, neutrophils, and monocytes in coagulation and vascular dysfunction. For instance, platelet counts are inversely correlated with extracellular histone levels in the Malawi cohort, and extracellular histones promote both procoagulant and barrier-disruptive pathways (22). Additionally, activated neutrophils, a source of extracellular histones, are linked to CM severity in the Malawi cohort (73, 74). The phenotypes and effector functions of platelets are modified by microvesicles and extracellular histones released by injured cells (75, 76) and neutrophils (77). Consequently, interactions between platelets and extracellular histones in cerebral microvessels may amplify localized coagulation and inflammatory processes (7, 63). These findings raise the possibility for divergent contributions of parasite biomass and Group A–EPCR binding variants in disease etiology of patients with CM with mild or severe thrombocytopenia.

A limitation of the present study is that, despite using the WHO criteria to diagnose patients with UM in Rourkela, subjects were enrolled after admission to the hospital. It is therefore plausible that patients with UM in our Indian setting were sicker than those with milder infections in Malawi, who did not require hospitalization. Additionally, we did not include a pediatric UM group in Rourkela because of the challenges associated with performing a 35-minute MRI scan on noncomatose children. Lastly, we applied RF models due to their ability to assess variable importance even with multicollinearity of *var* primers. However, logistic or linear regression modeling could have the additional useful property of fitting coefficients, reflecting how a change in *var* gene expression or other variables affects the likelihood of CM. Despite these limitations, this is the first study, to our knowledge, to analyze determinants of CM in a cohort of Indian patients that underwent MRI for brain swelling assessment.

Overall, our findings provide evidence that high parasite biomass and elevated EPCR-binding *var* transcripts are common parasite determinants of CM pathogenesis in both children and adults. Taken together, they further strengthen the role of parasite biomass as a pivotal pathogenic mechanism in CM and support the hypothesis that restoring normal function of the cytoprotective APC-EPCR signaling pathway may represent a promising therapeutic avenue for patients with CM.

## Methods

### Rourkela patients and samples.

Individuals were enrolled between October 2013 and August 2019 at IGH, a tertiary-level healthcare establishment owned by the Steel Authority of India Limited in Rourkela, India. Informed consent or assent were obtained from all study patients or their legal guardians prior to inclusion in the study. All malaria patients were treated according to the national drug policy of the Government of India (78), as detailed elsewhere (15). A physical examination was conducted upon admission at IGH, and clinical data were recorded and monitored on standardized case report forms. An age cut-off of 16 was used to separate children from adults in our cohort, based on previously published results demonstrating that the peak of total brain volume is reached at 15.2 years (79). Whole blood was collected in BD Vacutainer CTAD tubes (Becton Dickinson) containing a mixture of sodium citrate, theophylline, adenosine, and dipyridamole. Samples were immediately centrifuged at 1500*g* for 15 minutes before pelleted RBC were collected, mixed with TRIzol (Invitrogen, ratio of 1:9) and stored at –80°C until batch processing for RNA extraction.

### Laboratory and clinical diagnosis for malaria.

Falciparum malaria infection was confirmed by *P*. *falciparum* histidine-rich protein II antigen detection–based rapid diagnostic tests (SD Bioline, Standard Diagnostics), and standard microscopic examinations of Giemsa-stained thick and thin smears were carried out to corroborate species identification and assess parasitemia. All enrolled individuals were blood smear positive for *P*. *falciparum*. Results from routine hematological and biochemical analyses were also taken into consideration for the diagnosis of severe malaria. Patients were classified as CM, SNCM, or UM based on WHO severe malaria criteria: (a) coma (Glasgow coma score < 11 for adults and a Blantyre coma score ≤ 2 for children), (b) severe malarial anemia (Hb < 7 g/dL for adults and < 5 g/dL for children), (c) jaundice (bilirubin > 3 mg/dL), (d) acute kidney injury (serum creatinine > 3 mg/dL), and (e) hyperlactatemia (lactate > 5 mmol/L).

### Assessment of brain swelling and brain volume quantification.

MRI examinations were performed using a 1.5T Siemens Symphony MRI scanner (Siemens) as described elsewhere (15). The degree of brain swelling was assessed on T2w images and graded by a neuroradiologist blinded to the clinical classification of patients, using a 4-point score according to the degree of cortical swelling and sulcal effacement: 1, no brain swelling; 2, mild brain swelling characterized by cortical swelling and preserved sulci; 3, moderate brain swelling with more pronounced cortical swelling and narrowing of adjacent cerebrospinal fluid-filled sulci; and 4, severe brain swelling defined by complete sulcal effacement (Figure 1A). Similar definitions of brain swelling were done in the Malawi cohort (36) with the 8-point scale converted to the 4-point swelling scale as follows: no brain swelling (swelling scores 1–3 = 1); mild (swelling score 4 = 2); moderate (swelling scores 5–6 = 3); severe (swelling scores 7–8 = 4).

### Malawi cohort.

The Malawi cohort was previously described (36). Both patients with CM or UM were recruited from Queen Elizabeth Central Hospital (QECH) in Blantyre, Malawi. Patients with CM were admitted to the Research Ward within QECH. The CM group included children between the ages of 6 months and 12 years and met the WHO classification of CM for *P*. *falciparum* parasitemia, a Blantyre coma score ≤ 2, and exclusion of other identifiable causes of coma. UM children presented to the Accident and Emergency Pediatric Department at QECH. Inclusion criteria for UM cases were children 1–12 years old, history of fever, normal mental health status as measured by Blantyre coma score, peripheral *P*. *falciparum* parasitemia, and no overt signs of compromised health, malnutrition, or progression to severe malaria.

### Parasite biomass quantification.

Plasma levels of PfHRP2 were measured to quantify total parasite biomass (38), using a commercially available ELISA kit (Malaria Ag CELISA kit, catalog KM2, Cellabs). Analyses were performed according to the manufacturer’s instructions, in duplicate for each patient, and absorbance was read at 450 nm using a Multiskan Spectrum ELISA plate reader (Thermo Fisher Scientific).

### RNA extraction and cDNA synthesis.

The pRBC-TRIzol cryopreserved tubes were thawed on ice, and total RNA was extracted using the RNeasy micro kit (Qiagen), following the manufacturer’s instructions. cDNA was then generated using the MultiScribe reverse transcriptase (Thermo Fisher Scientific). A total of 300 μL cDNA was synthesized per patient sample.

### Transcriptional profiling of var by qPCR.

We selected a combination of degenerate primers that specifically cover Group A and EPCR binding domains (45), CD36 binding domains (34), and dual EPCR–ICAM-1 PfEMP1 binders (47). qPCR was carried out using 5 μL of cDNA template per reaction well, along with Power SYBR Green PCR Master Mix (Thermo Fisher Scientific) and the specific primers in an ABI 7500 Fast Real Time PCR system (Applied Biosystems). PCR cycle amplification parameters included an initial denaturation step at 95°C for 15 minutes, followed by 40 cycles of denaturation for 30 seconds at 95°C, annealing for 40 seconds at 50°C, and extension for 50 seconds at 65°C. Data acquisition was enabled after the extension step of each amplification cycle followed by a final extension step of 40 seconds at 68°C and a melt-curve analysis. Levels of *var* transcription were determined by calculating the relative quantification of the average expression of adenylosuccinate lyase and seryl-tRNA synthetase housekeeping genes (ΔCt var_primer = Ct var_specific primer – Ct average_housekeeping primers). Transcript levels of *var* domains were represented as transcript units (Tu), which were calculated as: Tu = 2^(5−ΔCt)^ (34). The cut-off Ct (average of the housekeeping genes) for inclusion in analysis was < 31.

### NGS library preparation and sequencing.

DBLα tag libraries from 22 patients (19 CM, 2 SNCM, 1 UM) were prepared as described previously (36) with some modifications. DBLα tags were PCR amplified from cDNA in 26–41 cycles using the previously published varF_dg2 and brlong2 primers (34) with the addition of 5′ and 3′ MiSeq adaptor sequences (5′-TCGTCGGCAGCGTCAGATGTGTATAAGAGACAGGCAMGMAGTTTYGCNGATATWGG-3′ and 5′-GTCTCGTGGGCTCGGAGATGTGTATAAGAGACAGTCTTCDSYCCATTCVTCRAACCA-3′) at a final concentration of 0.6 μM. The following PCR conditions were used: 98°C for 45 seconds, 98°C for 10 seconds, 50°C for 20 seconds, 68°C for 40 seconds, and 72°C for 7 minutes. Amplicons were purified using SPRIselect magnetic beads (Beckman-Coulter) and were used as a template for the next round of PCR amplification, achieved in 10 cycles using Nextera P7 and P5 index primers (Illumina) and the PCR conditions described above. Following amplicon purification, libraries were quantified by qPCR (KAPA library quantification kit; Kapa Biosystems) on the StepOnePlus Real-Time PCR System (Applied Biosystems) and pooled in equimolar amounts to a concentration of 4 nM. The pooled library was then denatured according to Illumina MiSeq instructions. A similarly denatured PhiX control library was added at a final concentration of 40% to compensate for the AT richness of *P*. *falciparum*. The final sample was sequenced using a 600-cycle v3 kit on the MiSeq Sequencer (Illumina).

### Processing of DBLα tag sequences.

Sequences were processed largely as described previously (36, 80), with some modifications. Briefly, amplicon sequences were reconstructed by assembly of forward and reverse reads using FLASH (81), with removal of nonoverlapping reads. Primer and adaptor sequences were trimmed from assembled reads, and any reads with missing primer sequences were discarded using Cutadapt (82). Sequences were deduplicated (while keeping track of duplicate counts), and sequences with low-confidence base calls (*N*) were discarded using FASTX-toolkit (http://hannonlab.cshl.edu/fastx_toolkit) (83). Reads from each patient were clustered using VSEARCH version v2.9.1 (84) at a 96% nucleotide identity cut-off, producing the final processed data set consisting of centroid sequences with their associated abundance (number of times observed in the raw data set). To identify the DBLα tags shared among patients, centroids from every pair of individual patient data sets were further clustered (using VSEARCH version v2.9.1) at a 96% nucleotide identity cut-off. Centroid sequences representing clusters with < 50 source sequences were excluded from further analyses (83).

### Functional annotation of DBLα tags.

DBLα tags were assigned a *var* type based on sequence similarity determined by BLAST searches (85) against a custom library of 513 annotated *var* genes (28, 36, 44). BLAST results for the top 5 hits in each search were then used to generate a cumulative score derived from E-values (sum of –log_10_[E value]) for each *var* type (e.g., groups B and C, DC8-EPCR). The best-scoring *var* type was assigned to each DBLα tag.

### Database accessibility of sequences.

All DBLα tag sequences have been submitted to GenBank, and accession numbers (MW014366 – MW014807) are in Supplemental Table 4.

### Statistics.

Univariate analyses were performed using GraphPad Prism v8 for Windows and R. Differences between groups were calculated using a 2-tailed *t* test or a 2-tailed Mann-Whitney *U* test. Correlations between variables were evaluated using Spearman’s rank correlation coefficient. Differences in CM/swelling scores between samples grouped by binary thrombocytopenia and parasitemia status were assessed by the χ^2^ test. Machine learning analyses were performed using R version 3.6.1. All Rourkela samples and Malawi samples with < 25% of *var* gene primers missing/below the limit of detection were included, and *var* gene Tu associated with missing primers were set to Tu = 1. RF binary classification models were fit using the R caret (86) and randomForest (87) packages to discriminate UM versus CM; UM versus CM^BS+^ (brain swelling score > 1, Supplemental Table 1); and CM^BS+^ versus CM^BS–^. For each comparison, the Tu values of *var* transcripts and optionally PfHRP2 and platelet levels were used as continuous predictors. During RF training, data are randomly divided into random splits, with replacement, of both samples and predictors (e.g., *var* transcripts, platelet levels) and decision trees fit on each subset, with the final result being a RF of decision tree aggregate predictions. Initial model performance was assessed using out-of-bag predictions (i.e., predictions on the data held out during each random split by the decision tree trained on the split). Model performance was scored as the area under the receiver-operator curve (ROC AUC), with associated 95% CIs calculated with the pROC package in R package. Models were considered significant when the lower 95% CI of AUC was greater than 0.5, where 0.5 corresponds to the expected performance of a random predictor. The contribution of individual *var* genes or other features to model performance was assessed as MDCA. The MDCA value is estimated during training as the difference in classifier accuracy for random splits that contain the predictor in question versus those that do not. For visualization and plotting purposes, where a decrease in a certain predictor was associated with a more severe outcome, a negatively signed MDCA score was shown. The mProbes algorithm (88) was used to identify predictors significantly associated with sample classes. Model cross-prediction performance was assessed by using models trained and parameterized on Malawi samples to make unbiased predictions on Rourkela samples, with ROC AUC and MDCA calculated as above.

### Study approval.

The study at IGH was approved by the Health Ministry Screening Committee, Government of India (TDR589/2010/ECDII), as well as the IRBs of IGH (IGH/DNB/2814), New York University School of Medicine (S12-03016), Western IRB (USA), and the London School of Hygiene & Tropical Medicine. The study in Malawi was approved by the IRBs at the University of Malawi College of Medicine, Michigan State University, and the Albert Einstein College of Medicine.

## Author contributions

PKS, MB, SCW, and JDS designed and conceptualized the study; RP, Anita Mohanty, RRM, MM, and S Sanjib Mohanty managed patients in Rourkela during their hospitalization and collected the clinical data. AH designed the MRI sequence. LP generated the brain volume data in Rourkela. Akshaya Mohanty, JB, and S Maharana performed clinical laboratory work in Rourkela. PKS oversaw the recruitment of study patients and the collection, processing, and storage of samples. PKS and MB carried out the qPCR and statistical analyses. SD, MV, and VV performed the NGS and bioinformatic analysis. FJD performed the machine learning models. TET, WM, SJR, KBS, and KK provided data from the Malawi cohort. TET, WM, SJR, KBS, KK, DNS, and JDA contributed to the results interpretation. PKS, SD, JDS, MB, and SCW wrote the manuscript, and all authors critically reviewed and approved the final manuscript.

## Supplementary Material

Supplemental data

## Version 1 09/22/2021

Electronic publication
