# Supplementary material for: Determinants of brain swelling in pediatric and adult cerebral malaria
Source: JCI Insight. 2021 Sep 22;6(18):e145823. doi: 10.1172/jci.insight.145823 (PMC8492338; doi:10.1172/jci.insight.145823)
Supplement: Supplemental data [file jciinsight-6-145823-s073.pdf]

SUPPLEMENTAL DATA

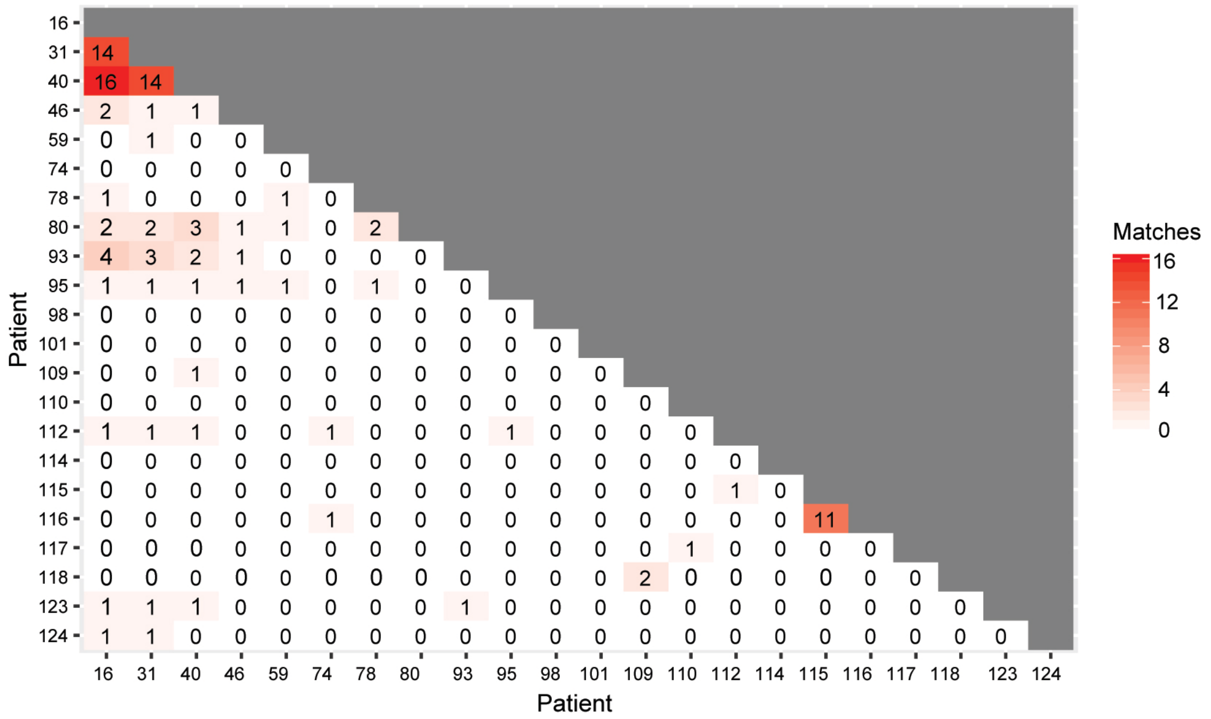

Supplemental Figure 1: Shared DBLα tags determined by NGS analysis.

Summary of the number of shared DBLα tags (≥96% nucleotide identity) between patients.

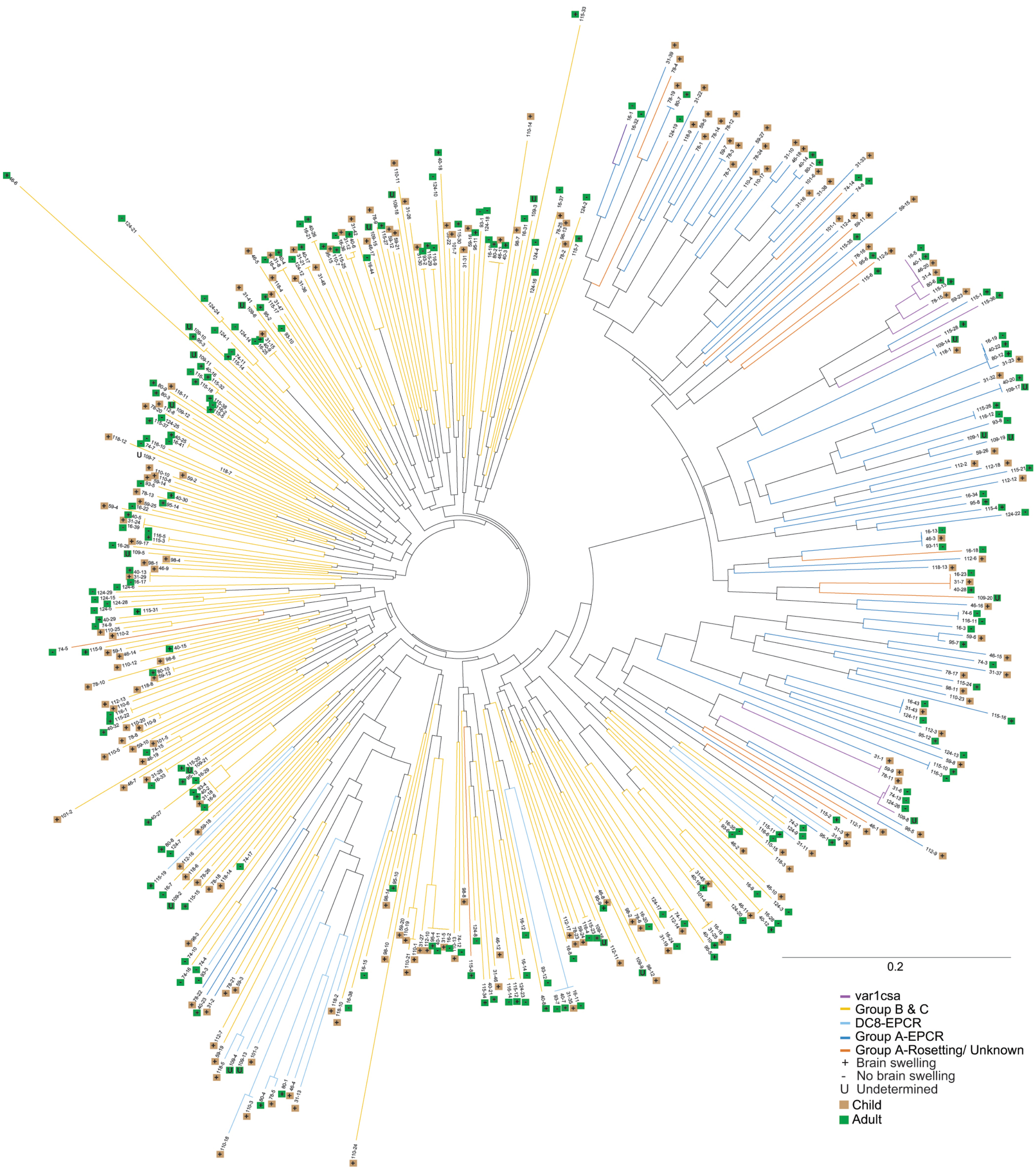

Supplemental Figure 2: Phylogenetic tree of DBLα tags from the Rourkela cohort labeled by brain swelling category, age and patient number. Neighbor-joining tree of translated DBLα sequences amplified from CM patients.

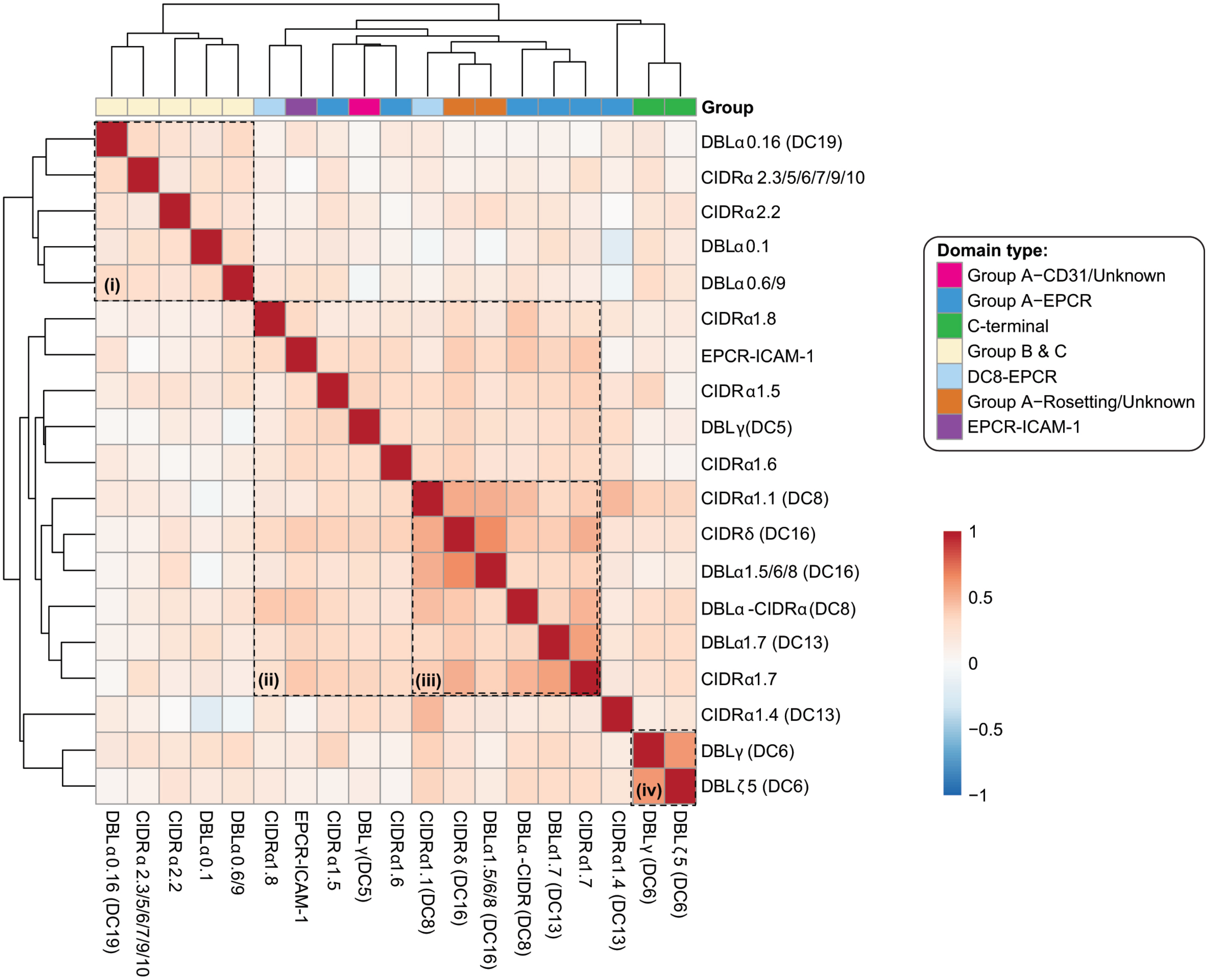

Supplemental Figure 3: Unsupervised hierarchical clustering of the var primers in the combined cohorts. Cluster (i) includes amplified transcripts from Group B and C, cluster (ii) from Group A and DC8-EPCR, cluster (iii) includes Group A-Rosetting/Unknown, Group A-EPCR and DC8-EPCR, cluster (iv) includes C-terminal domains from DC6.

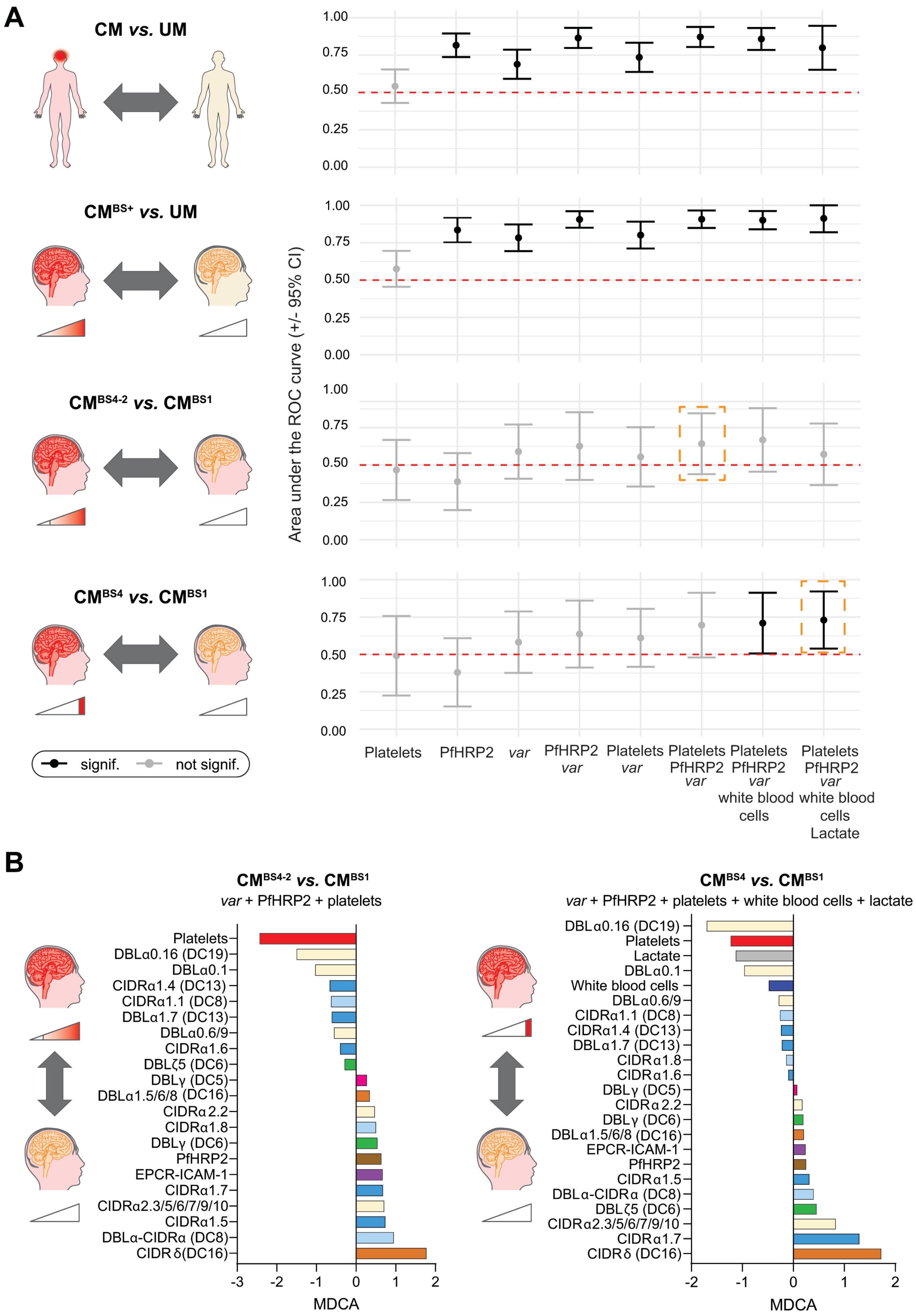
A
CM vs. UM
CM^BS+ vs. UM
CM^BS4-2 vs. CM^BS1
CM^BS4 vs. CM^BS1
signif.
not signif.
Area under the ROC curve (+/- 95% CI)
1.00
0.75
0.50
0.25
0.00
Platelets
PfHRP2
var
PfHRP2 var
Platelets var
Platelets PfHRP2 var
Platelets PfHRP2 var white blood cells
Platelets PfHRP2 var white blood cells Lactate
B
CM^BS4-2 vs. CM^BS1
var + PfHRP2 + platelets
Platelets
DBLα0.16 (DC19)
DBLα0.1
CIDRα1.4 (DC13)
CIDRα1.1 (DC8)
DBLα1.7 (DC13)
DBLα0.6/9
CIDRα1.6
DBLζ5 (DC6)
DBLγ (DC5)
DBLα1.5/6/8 (DC16)
CIDRα2.2
CIDRα1.8
DBLγ (DC6)
PfHRP2
EPCR-ICAM-1
CIDRα1.7
CIDRα2.3/5/6/7/9/10
CIDRα1.5
DBLα-CIDRα (DC8)
CIDRδ(DC16)
-3
-2
-1
0
1
2
MDCA
CM^BS4 vs. CM^BS1
var + PfHRP2 + platelets + white blood cells + lactate
DBLα0.16 (DC19)
Platelets
Lactate
DBLα0.1
White blood cells
DBLα0.6/9
CIDRα1.1 (DC8)
CIDRα1.4 (DC13)
DBLα1.7 (DC13)
CIDRα1.8
CIDRα1.6
DBLγ (DC5)
CIDRα2.2
DBLγ (DC6)
DBLα1.5/6/8 (DC16)
EPCR-ICAM-1
PfHRP2
CIDRα1.5
DBLα-CIDRα (DC8)
DBLζ5 (DC6)
CIDRα2.3/5/6/7/9/10
CIDRα1.7
CIDRδ (DC16)
-2
-1
0
1
2
MDCA

Supplemental Figure 4: Performance of predictors in machine learning models

(A). Performance of random forest models trained on different combinations of host and parasite factors in discriminating CM *vs.* UM, CM with brain swelling (CM^{BS+}) *vs*. UM, CM with brain swelling (CM^{BS2-4}) *vs*. CM without brain swelling (CMBS1), and CM with severe brain swelling (CM^{BS4}) *vs*. CM without brain swelling (CM^{BS1}) in Malawi and India cohorts. Points indicate model performance in terms of ROC AUC with error bars showing confidence intervals (CI). The orange dashed boxes indicates models shown in the bar graphs in B. (B) Bar graphs representing the importance of parasite and host features in random forest models that classify CM with brain swelling (CM^{BS2-4}) *vs*. CM without brain swelling (CM^{BS1}) (left panel), and CM with severe brain swelling (CM^{BS4}) *vs*. CM without brain swelling (CM^{BS1}) (right panel).

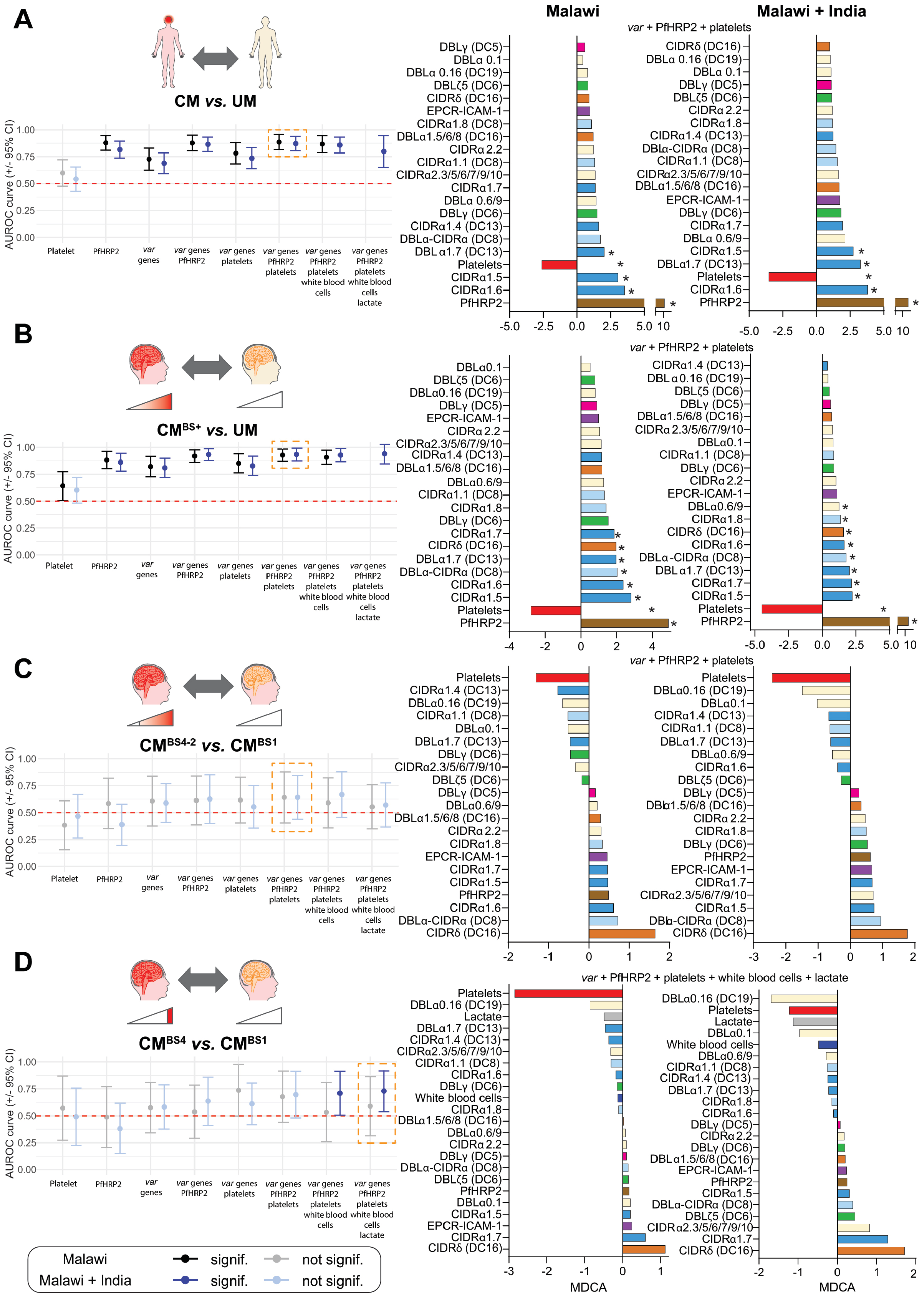

A
CM vs. UM
Malawi
Malawi + India
var + PfHRP2 + platelets
DBLγ (DC5)
DBLα 0.1
DBLα 0.16 (DC19)
DBLζ5 (DC6)
CIDRδ (DC16)
EPCR-ICAM-1
CIDRα1.8 (DC8)
DBLα1.5/6/8 (DC16)
CIDRα2.2
CIDRα1.1 (DC8)
CIDRα2.3/5/6/7/9/10
CIDRα1.7
DBLα 0.6/9
DBLγ (DC6)
CIDRα1.4 (DC13)
DBLα-CIDRα (DC8)
DBL α1.7 (DC13)
Platelets
CIDRα1.5
CIDRα1.6
PfHRP2
B
CM^BS+ vs. UM
C
CM^BS4-2 vs. CM^BS1
D
CM^BS4 vs. CM^BS1
var + PfHRP2 + platelets + white blood cells + lactate
Lactate
White blood cells
AUROC curve (+/- 95% CI)
Platelet
PfHRP2
var genes
var genes PfHRP2
var genes platelets
var genes PfHRP2 platelets
var genes PfHRP2 platelets white blood cells
var genes PfHRP2 platelets white blood cells lactate
MDCA
Malawi
Malawi + India
signif.
not signif.

Supplemental Figure 5: Comparison of random forest model performance between the Malawi cohort alone and the combined Malawi and India cohort.

Models were trained on different combinations of host and parasite factors in discriminating (A) CM *vs.* UM, (B) CM with brain swelling (CM^{BS+}) *vs*. UM, (C) CM with brain swelling ($CM^{BS2\text{-}4}$) *vs*. CM without brain swelling (CM^{BS1}), and (D) CM with severe brain swelling (CM^{BS4}) *vs*. CM without brain swelling (CM^{BS1}). Left: Points indicate model performance in terms of ROC AUC with error bars showing confidence intervals (CI). The orange dashed boxes indicate which models are represented in the right. Right: Bar graphs represent the importance of factors in random forest models. Positive MDCA indicates greater presence in CM patients (A), CM with brain swelling (CM^{BS+} or $CM^{BS2\text{-}4}$) (B and C), and CM with severe brain swelling (CM^{BS4}) (D). Asterisks: significant difference between groups measured by mProbes algorithm ([FWER] of ≤ 0.2).

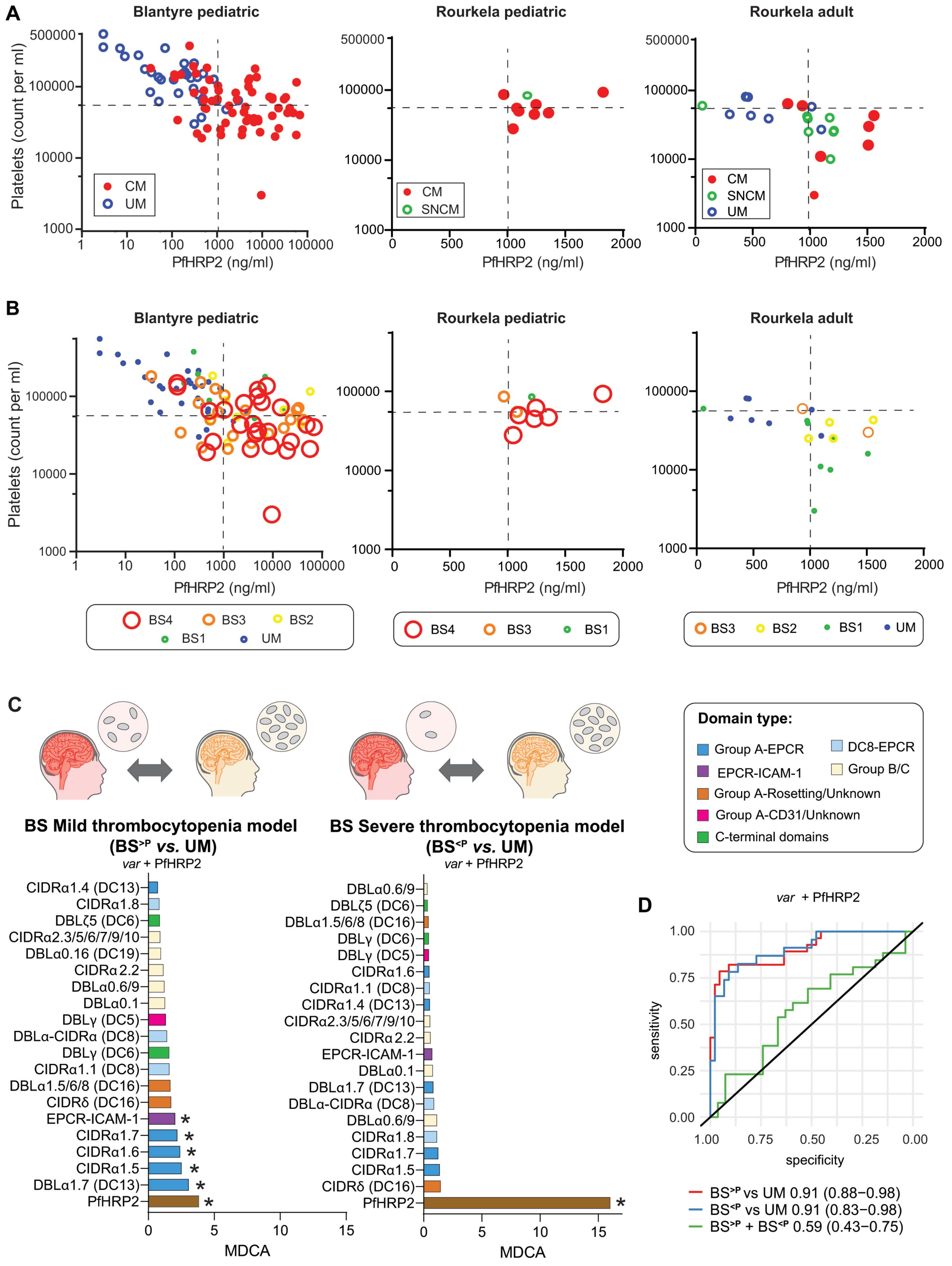
A
Blantyre pediatric
Rourkela pediatric
Rourkela adult
Platelets (count per ml)
PfHRP2 (ng/ml)
CM
UM
SNCM
B
BS4
BS3
BS2
BS1
UM
C
BS Mild thrombocytopenia model
(BS>P vs. UM)
var + PfHRP2
BS Severe thrombocytopenia model
(BS<P vs. UM)
var + PfHRP2
Domain type:
Group A-EPCR
DC8-EPCR
EPCR-ICAM-1
Group B/C
Group A-Rosetting/Unknown
Group A-CD31/Unknown
C-terminal domains
CIDRα1.4 (DC13)
CIDRα1.8
DBLζ5 (DC6)
CIDRα2.3/5/6/7/9/10
DBLα0.16 (DC19)
CIDRα2.2
DBLα0.6/9
DBLα0.1
DBLγ (DC5)
DBLα-CIDRα (DC8)
DBLγ (DC6)
CIDRα1.1 (DC8)
DBLα1.5/6/8 (DC16)
CIDRδ (DC16)
EPCR-ICAM-1 *
CIDRα1.7 *
CIDRα1.6 *
CIDRα1.5 *
DBLα1.7 (DC13) *
PfHRP2 *
DBLα0.6/9
DBLζ5 (DC6)
DBLα1.5/6/8 (DC16)
DBLγ (DC6)
DBLγ (DC5)
CIDRα1.6
CIDRα1.1 (DC8)
CIDRα1.4 (DC13)
CIDRα2.3/5/6/7/9/10
CIDRα2.2
EPCR-ICAM-1
DBLα0.1
DBLα1.7 (DC13)
DBLα-CIDRα (DC8)
DBLα0.6/9
CIDRα1.8
CIDRα1.7
CIDRα1.5
CIDRδ (DC16)
PfHRP2 *
MDCA
D
var + PfHRP2
sensitivity
specificity
BS>P vs UM 0.91 (0.88−0.98)
BS<P vs UM 0.91 (0.83−0.98)
BS>P + BS<P 0.59 (0.43−0.75)

Supplemental Figure 6: Machine learning models of brain swelling in CM patients with different platelet levels. (A) Scatter plot showing the relationship between platelet levels, PfHRP2 levels and disease category in Blantyre and Rourkela. Dashed lines indicate divisions into high/low parasite biomass and thrombocytopenia groups for each cohort. (B) Scatter plot showing the relationship between platelet levels, PfHRP2 levels and brain swelling score in Blantyre and Rourkela. Dashed lines indicate divisions into high/low parasite biomass and thrombocytopenia groups for each cohort. (C) Bar graphs representing the importance of parasite factors in random forest models that classify brain swelling *vs*. UM patients with different degrees of thrombocytopenia. Left: Brain swelling patients with mild thrombocytopenia *vs.* UM. Right: Brain swelling with severe thrombocytopenia *vs*. UM. Positive MDCA indicates greater presence in brain swelling than in UM patients. Asterisks: significant difference between groups measured by mProbes algorithm ([FWER] of ≤ 0.2). (D) ROC curves showing the predictive performance of the models. Performance is measured by area under the curve, indicated in the legend with 95% confidence intervals in parentheses.

Supplemental Table 1: Cohort characteristics. Clinical, laboratory and radiological features of the 39 patients included in the study.

	ALL PATIENTS (n=38)*			*ADULTS (n=27)*			*CHILDREN (n=11)*		
**Patient Characteristics**	**Swelling**	**No swelling**	***p-value***	**Swelling**	**No swelling**	***p-value***	**Swelling**	**No swelling**	***p-value***
Sample size (n)	18	20		8	19		10	1	
Age (years), Median [IQR]	11 [5- 25.5]	31.5 [22.25-45]	***0.0004***	26 [24.2, 29.5]	33 [26, 45]	*0.1*	5 [5, 9.25]	4 [4, 4]	*0.4*
Male, n (%)	12 (66.6)	12 (60)		4 (50)	12 (63.1)		08 (80)	0 (0)	
Parasitemia (/µL), Median [IQR]	10780 [397, 73910]	236060 [26875, 608438]	***0.003***	4974 [313, 14832]	299970 [17500, 66000]	***0.002***	20135 [1160, 148524]	126500 [126500, 126500]	*0.8*
PfHRP2 (ng/ml), Median [IQR]	1172 [1010, 1354]	1014 [458.9, 1172]	***0.04***	1172 [987.1, 1513]	997.3 [453.9, 1172]	***0.09***	1165 [1028, 1353]	1172 [1172, 1172]	*0.8*
Platelet (x10^3/µL), Median [IQR]	43 [29, 57.5]	42.5 [23.5, 65.0]	*0.8*	38 [26.25, 42.25]	42 [21, 59]	*0.5*	50 [36.5, 74]	85 [85, 85]	*0.2*
Hemoglobin (g/dL), Median [IQR]	7 [6.05, 8.37]	7.95 [5.85, 11.3]	*0.2*	8.1 [6.60, 8.52]	8.3 [6.3, 11.4]	*0.6*	6.45 [5.55, 8.1]	4.9 [4.9, 4.9]	*0.2*
Lactate (mmol/L), Median [IQR]	3.60 [2.55, 6.87]	7.0 [2.45, 16.5]	*0.3*	3.8 [3.0, 8.6]	7 [2.45, 16.5]	*0.7*	3.4 [2.4, 6.3]	-	-
Bilirubin (mg/dL), Mean ± SEM	5.53 ± 1.61	3.77 ± 1.34	*0.4*	8.8 ± 2.82	3.77 ± 1.34	*0.07*	2.25 ± 0.32	-	-
Creatinine (mg/dL), Mean ± SEM	2.39± 0.7	2.17 ± 0.45	*0.7*	3.85 ± 1.32	2.17 ± 0.45	*0.1*	1.2 ± 0.5	-	-
Coma score, Median [IQR]	8 [6, 11]	15 [10, 15]	***0.0003***	8 [7, 15]	15 [10, 15]	***0.002***	7.5 [5.5, 8.5]	15 [15, 15]	*0.06*
Severe malarial anemia, n (%)	4 (22.2)	8 (40)		2 (25)	7 (36.9)		2 (20)	1 (100)	
Jaundice, n (%)	9 (50)	6 (30)		7 (87.5)	6 (31.6)		2 (20)	0 (0)	
Acute kidney injury, n (%)	5 (27.7)	4 (20)		4 (50)	4 (21)		1 (10)	0 (0)	
Respiratory distress n (%)	0 (0)	0 (0)		0 (0)	0 (0)		0 (0)	0 (0)	
Mortality, n (%)	1 (5.5)	0 (0)		0 (0)	0 (0)		1 (10)	0 (0)	
Brain swelling, n (%)	18 (100)	0 (0)		8 (100)	0 (0)		10 (100)	0 (0)	
**Brain swelling score (scale 1-4)**									
1 - no swelling, n (%)	0 (0)	20 (100)		0 (0)	19 (100)		0 (0)	1 (100)	
2 - mild swelling, n (%)	5 (27.8)	0 (0)		5 (62.5)	0 (0)		0 (0)	0 (0)	
3 - moderate swelling, n (%)	6 (33.3)	0 (0)		3 (37.5)	0 (0)		3 (30)	0 (0)	
4 - severe swelling, n (%)	7 (38.9)	0 (0)		0 (0)	0 (0)		7 (70)	0 (0)	

*Brain swelling scores were undetermined due to motion artefacts during MRI, for one patient (ID#109). IQR, interquartile range, SEM: Standard error of mean. p-values estimated from Mann Whitney test or unpaired t-test.

Supplemental Table 2: Characteristics of malaria patients excluded from *var* analyses (related to Table 1).

**Patient Characteristic**	**var Typed cases (n = 39)**	**Excluded cases (n = 92)**	***p-value***
**Age (years), median [IQR]**	26 [10, 35]	29.5 [20.25, 47.0]	***0.0481***
**Female: Male, n (%)**	14 (35.9): 25 (64.9)	24 (26.1): 68 (73.9)	
**Smear score, median [IQR]**	NA	NA	
**Parasitemia (/µl), median [IQR]**	66880 [4080, 338250]	2730 [409, 60000]	***0.0028***
**PfHRP2 (ng/ml), median [IQR]**	1080 [869.8, 1221]	133.6 [13.06, 878.9]	***<0.0001***
**Hemoglobin (g/dl), median [IQR]**	7.6 [5.7, 10.1]	8.5 [6.67, 10.5]	*0.17*
**Platelets (10^3/µl), median [IQR]**	43 [27.25, 60.0]	50 [29.35, 93.0]	*0.06*
**Total WBC (10^3/µl), median [IQR]**	6.8 [4.8, 11.7]	7.8 [5.4, 14.0]	*0.31*
**Lactate (mmol/L), median [IQR]**	3.8 [2.6, 12.5]	4.2 [2.5, 6.7]	*0.13*
**Glasgow coma score, median [IQR]**	15 [8,15]	11 [9,15]	*0.8*
**Severe malarial anemia, adults n (%)**	9 (32.1)	19 (26.4)	
**Severe malarial anemia, children n (%)**	3 (27.2)	2 (11.1)	
**Respiratory distress, n (%)**	1(2.6)	10 (10.9)	
**Brain swelling, n (%)**	18 (47.4) [§]	36 (41.9) [§]	*0.16*
**Brain Swelling score (scale 1-4)**			
1- No swelling, n (%)	20 (52.6)	50 (58.1)	
2-Mild swelling, n (%)	5 (13.2)	21 (24.4)	
3-Moderate swelling, n (%)	6 (15.8)	5 (5.8)	
4-Severe swelling, n (%)	7 (18.4)	10 (11.6)	
**Mortality**	2 (5.1)	7 (7.6)	

[§] Brain swelling scores were unavailable due to motion artefacts during MRI, for one patient in included group, hence (n=38) and 6 patients in excluded group, hence (n=86). IQR, interquartile range; p-values estimated as per Wilcoxon rank-sum or unpaired t-test.

Supplemental Table 3. Table showing number of DBLα tags per patient at the 90% cut-off.

**Patient #**	**Patient ID**	**0.9 cut-off**
1	16	44
2	31	48
3	40	32
4	46	20
5	59	27
6	74	17
7	78	26
8	80	12
9	93	13
10	95	15
11	98	17
12	101	7
13	109	21
14	110	25
15	112	18
16	114	14
17	115	38
18	116	14
19	117	14
20	118	14
21	123	4
22	124	29

Supplemental Table 4. DBLα tags amplified from patients. Related to Figure 2.

Patient ID	Abun-dance ranking	GenBank Accession #	Cluster size[a]	Total count[b]	Dominant count[c]	Dominant score[d]	Dominant type[e]	Top hit	Top hit E-val	Top type	2nd hit	2nd hit E-val	2nd hit type	3rd hit	3rd hit E-val	3rd hit type	4th hit	4th hit E-val	4th hit type	5th hit	5th hit E-val	5th hit type
16	1	MW014366	24832	5	5	435.0	EPCR	IGHvar39	2.14E-95	EPCR	KX154909.1	4.67E-87	EPCR	KX154890.1	2.17E-85	EPCR	KX154923.1	2.17E-85	EPCR	KX154937.1	2.17E-85	EPCR
	2	MW014367	10799	5	5	327.9	Group B/C	IT4var33	9.31E-85	Group B/C	PFL1955w	2.06E-66	Group B/C	KX154819.1	5.77E-62	Group B/C	IGHvar31	9.65E-60	Group B/C	KX154910.1	1.25E-58	Group B/C
	3	MW014368	9584	5	5	521.7	EPCR	KX154875.1	9.76E-109	EPCR	KX154879.1	9.76E-109	EPCR	KX154949.1	9.76E-109	EPCR	AAQ73928	2.11E-105	EPCR	KX154818.1	9.97E-94	EPCR
	4	MW014369	8098	5	5	164.1	Group B/C	IT4var63	4.07E-43	Group B/C	HB3var47	1.48E-32	Group B/C	HB3var09	1.48E-32	Group B/C	DD2var44	6.91E-31	Group B/C	DD2var35	1.16E-28	Group B/C
	5	MW014370	7316	5	5	869.1	var1csa	DD2var25	2.50E-179	var1csa	AJ420411	2.50E-179	var1csa	KX154917.1	4.19E-177	var1csa	IGHvar11	2.54E-169	var1csa	AAM55194	1.18E-167	var1csa
	6	MW014371	6881	5	5	274.5	Group B/C	IGHvar08	9.49E-60	Group B/C	PFL1960w	2.06E-56	Group B/C	DD2var34	2.66E-55	Group B/C	PFCLINvar54	3.44E-54	Group B/C	DD2var18	1.60E-52	Group B/C
	7	MW014372	5679	5	4	159.6	Group B/C	PFCLINvar65	1.81E-41	Group B/C	IT4var59	1.81E-41	Group B/C	HB3var27	1.81E-41	Group B/C	MAL8P1.220	3.91E-38	Group B/C	IT4var06	1.82E-36	DC8
	8	MW014373	3791	5	5	135.8	Group B/C	IT4var41	6.69E-41	Group B/C	DD2var28	6.83E-26	Group B/C	KX154822.1	3.18E-24	Group B/C	KX154883.1	3.18E-24	Group B/C	KX154955.1	3.18E-24	Group B/C
	9	MW014374	3511	5	5	237.0	Group B/C	PFL0005w	2.85E-94	Group B/C	IGHvar15	1.11E-38	Group B/C	IGHvar21	3.99E-38	Group B/C	HB3var16	6.67E-36	Group B/C	KX154815.1	1.12E-33	Group B/C
	10	MW014375	3226	5	3	105.5	Group B/C	IT4var32b	1.91E-46	DC8	IT4var19	1.91E-46	DC8	RAJ116var10	6.98E-36	Group B/C	IT4var41	6.98E-36	Group B/C	DD2var39	6.98E-36	Group B/C
	11	MW014376	2830	5	3	158.2	DC8	KX154825.1	6.88E-71	DC8	KX154946.1	1.95E-56	DC8	IT4var39	1.55E-37	Group B/C	DD2var49	2.60E-35	EPCR	KX154870.1	4.35E-33	DC8
	12	MW014377	2753	5	4	201.6	Group B/C	KX154900.1	2.66E-65	Group B/C	PFCLINvar30	1.63E-52	DC8	IT4var30	1.63E-52	Group B/C	IT4var33	2.74E-45	Group B/C	PFB0010w	2.13E-41	Group B/C
	13	MW014378	2498	5	5	553.4	EPCR	KX154890.1	4.44E-122	EPCR	KX154923.1	9.62E-119	EPCR	KX154937.1	9.62E-119	EPCR	KX154857.1	4.54E-107	EPCR	KX154909.1	2.16E-90	EPCR
	14	MW014379	2309	5	4	106.8	Group B/C	IGHvar20	3.88E-38	Group B/C	PFCLINvar30	8.39E-35	DC8	KX154864.1	2.37E-25	Group B/C	IT4var17	8.51E-25	Group B/C	KX154832.1	1.84E-21	Group B/C
	15	MW014380	1861	5	4	171.4	Group B/C	KX154933.1	2.21E-60	DC8	PFCLINvar09	4.85E-47	Group B/C	IT4var13	1.36E-42	Group B/C	DD2var21	1.36E-42	Group B/C	PFCLINvar11	4.88E-42	Group B/C
	16	MW014381	1852	5	5	185.7	Group B/C	KX154848.1	9.33E-55	Group B/C	HB3var21	2.06E-36	Group B/C	IGHvar21	3.45E-34	Group B/C	IT4var29	4.46E-33	Group B/C	IT4var32a	7.47E-31	Group B/C
	17	MW014382	1487	5	5	210.6	Group B/C	PFCLINvar66	7.51E-56	Group B/C	IGHvar35	3.54E-44	Group B/C	RAJ116var30	2.13E-41	Group B/C	RAJ116var21	1.28E-38	Group B/C	IT4var39	3.60E-34	Group B/C
	18	MW014383	1483	5	3	240.6	Group A-Rosetting/Unknown	IT4var60	1.03E-88	Group A-Rosetting/Unknown	DD2var22	3.77E-78	Group A-Rosetting/Unknown	KX154959.1	1.35E-77	EPCR	IGHvar12	6.30E-76	Group A-Rosetting/Unknown	KX154890.1	1.36E-72	EPCR
	19	MW014384	1478	5	3	262.3	EPCR	IT4var02	4.41E-127	Group A-Rosetting/Unknown	KX154871.1	9.76E-109	EPCR	HB3var04	2.19E-80	Group A-Rosetting/Unknown	IT4var18	3.66E-78	EPCR	KX154893.1	1.32E-77	EPCR
	20	MW014385	1419	5	5	306.5	Group B/C	KX154862.1	2.99E-79	Group B/C	KX154851.1	1.09E-68	Group B/C	RAJ116var06	8.56E-60	Group B/C	IGHvar25	6.67E-56	Group B/C	MAL7P1.55	1.88E-46	Group B/C
	21	MW014386	1046	5	5	193.3	Group B/C	DD2var45	7.70E-61	Group B/C	HB3var30	1.71E-37	Group B/C	HB3var18	2.87E-35	Group B/C	MAL8P1.220	6.21E-32	Group B/C	PFD0005w	2.23E-31	Group B/C
	22	MW014387	962	5	5	303.9	Group B/C	AY028643	5.79E-87	Group B/C	KX154971.1	2.77E-65	Group B/C	IGHvar40	1.31E-53	Group B/C	IGHvar03	2.19E-51	Group B/C	PF13_0364	2.83E-50	Group B/C
	23	MW014388	930	5	3	196.8	Group A-Rosetting/Unknown	KX154844.1	1.30E-72	Group A-Rosetting/Unknown	KX154891.1	2.19E-65	Group A-Rosetting/Unknown	HB3var1csa	2.84E-64	var1csa	KX154842.1	6.14E-61	Group A-Rosetting/Unknown	KX154869.1	1.35E-47	EPCR
	24	MW014389	889	5	5	230.9	Group B/C	KX154841.1	1.35E-72	Group B/C	RAJ116var33	1.37E-62	Group B/C	PFL0005w	1.09E-43	Group B/C	KX154851.1	2.39E-30	Group B/C	KX154970.1	2.41E-25	Group B/C
	25	MW014390	878	5	5	320.6	Group B/C	IT4var34	4.50E-73	Group B/C	PFCLINvar39	1.26E-68	Group B/C	PFD0630c	1.27E-63	Group B/C	KX154826.1	5.91E-62	Group B/C	PFD0635c	5.95E-57	Group B/C
	26	MW014391	778	5	4	161.1	Group B/C	IT4var68	1.22E-53	Group B/C	IT4var62	2.05E-46	Group B/C	PFD1245c	4.50E-33	Group B/C	PFCLINvar07	7.53E-31	Group B/C	IT4var06	3.50E-29	DC8
	28	MW014392	726	5	5	227.6	Group B/C	PFL0005w	6.17E-91	Group B/C	RAJ116var06	1.40E-52	Group B/C	PF08_0103	5.19E-32	Group B/C	HB3var16	3.13E-29	Group B/C	PFC0005w	1.88E-26	Group B/C
	29	MW014393	718	5	5	302.9	Group B/C	IT4var34	3.39E-69	Group B/C	DD2var18	2.64E-65	Group B/C	KX154826.1	1.24E-58	Group B/C	PFD0630c	5.76E-57	Group B/C	PF13_0364	2.07E-56	Group B/C
	31	MW014394	654	5	3	131.0	Group B/C	HB3var13	1.55E-52	Group B/C	IT4var33	7.25E-46	Group B/C	KX154825.1	2.63E-40	DC8	PFCLINvar30	5.69E-37	DC8	KX154866.1	9.51E-35	Group B/C

	32	MW014395	644	5	4	280.3	EPCR	IGHvar39	4.64E-92	EPCR	RAJ116var19	7.98E-70	Group A-Rosetting/Unknown	KX154923.1	2.24E-65	EPCR	KX154937.1	2.24E-65	EPCR	KX154857.1	2.25E-60	EPCR
	33	MW014396	625	5	5	234.7	Group B/C	RAJ116var24	1.44E-52	Group B/C	PFC1120c	4.04E-48	Group B/C	PFL0020w	5.23E-47	Group B/C	KX154831.1	2.43E-45	Group B/C	DD2var24	2.43E-45	Group B/C
	34	MW014397	620	5	3	209.0	EPCR	DD2var40	1.03E-73	EPCR	DD2var09a	1.03E-73	EPCR	DD2var09b	1.03E-68	Group A-Rosetting/Unknown	KX154886.1	1.04E-63	EPCR	PFF0020c	4.84E-62	DC8
	35	MW014398	620	5	4	170.1	Group B/C	PFCLINvar15	8.53E-75	Group B/C	DD2var26	1.89E-56	Group B/C	KX154928.1	1.97E-26	DC8	IT4var29	5.51E-22	Group B/C	KX154884.1	9.23E-20	Group B/C
	36	MW014399	576	5	5	214.8	Group B/C	KX154819.1	6.32E-61	Group B/C	KX154880.1	4.99E-47	Group B/C	PFCLINvar66	3.02E-39	Group B/C	KX154860.1	1.41E-37	Group B/C	PFCLINvar64	1.10E-33	Group B/C
	37	MW014400	570	5	5	194.8	Group B/C	IGHvar34	1.35E-82	Group B/C	PFCLINvar47	2.42E-30	Group B/C	DD2var45	2.42E-30	Group B/C	PFCLINvar44	1.45E-27	Group B/C	DD2var46	1.45E-27	Group B/C
	38	MW014401	554	5	3	145.6	Group B/C	DD2var37	3.88E-58	Group B/C	IT4var33	1.09E-48	Group B/C	MAL6P1.316	5.12E-42	DC8	KX154942.1	1.84E-41	DC8	PFL1955w	6.62E-41	Group B/C
	39	MW014402	534	5	3	131.5	Group B/C	KX154866.1	9.31E-50	Group B/C	PFCLINvar39	7.25E-46	Group B/C	KX154928.1	1.57E-42	DC8	KX154829.1	4.40E-38	Group B/C	IT4var32b	7.35E-36	DC8
	41	MW014403	520	5	5	218.5	Group B/C	HB3var28	1.55E-57	Group B/C	IGHvar40	5.65E-47	Group B/C	RAJ116var30	9.53E-40	Group B/C	PFCLINvar11	3.43E-39	Group B/C	IT4var05	1.23E-38	Group B/C
	43	MW014404	508	5	4	359.2	EPCR	IT4var07	5.99E-106	EPCR	PF11_0521	4.69E-97	EPCR	IT4var08	2.20E-90	Group A-Rosetting/Unknown	KX154945.1	1.02E-88	EPCR	KX154903.1	2.26E-70	EPCR
	44	MW014405	490	5	5	195.7	Group B/C	DD2var18	3.32E-44	Group B/C	IGHvar17	1.54E-42	Group B/C	KX154922.1	9.29E-40	Group B/C	HB3var32	1.55E-37	Group B/C	RAJ116var26	2.60E-35	Group B/C
31	1	MW014406	14201	5	4	347.0	EPCR	HB3var02	1.20E-122	EPCR	DD2var32	4.50E-92	EPCR	IGHvar10	2.79E-69	Group A-Rosetting/Unknown	KX154818.1	3.61E-68	EPCR	IGHvar30	4.67E-67	EPCR
	2	MW014407	13827	5	3	225.1	EPCR	KX154941.1	1.24E-87	DC8	IGHvar27	5.83E-76	EPCR	HB3var03	5.83E-76	EPCR	KX154935.1	2.10E-75	EPCR	KX154873.1	9.76E-74	DC8
	3	MW014408	7203	5	3	286.3	Group A-Rosetting/Unknown	HB3var01	5.40E-131	Group A-Rosetting/Unknown	DD2var04	7.34E-95	Group A-Rosetting/Unknown	PF08_0141	1.28E-62	Group A-Rosetting/Unknown	AAQ73928	2.19E-45	EPCR	KX154875.1	4.74E-42	EPCR
	4	MW014409	7137	5	5	869.1	var1csa	DD2var25	2.50E-179	var1csa	AJ420411	2.50E-179	var1csa	KX154917.1	4.19E-177	var1csa	IGHvar11	2.54E-169	var1csa	AAM55194	1.18E-167	var1csa
	5	MW014410	7071	5	5	327.9	Group B/C	IT4var33	9.31E-85	Group B/C	PFL1955w	2.06E-66	Group B/C	KX154819.1	5.77E-62	Group B/C	IGHvar31	9.65E-60	Group B/C	KX154910.1	1.25E-58	Group B/C
	6	MW014411	5457	5	5	849.2	var1csa	RAJ116var02	5.15E-171	var1csa	PFCLINvar76	5.15E-171	var1csa	AF547159	5.15E-171	var1csa	KX154858.1	1.85E-170	var1csa	KX154820.1	2.40E-169	var1csa
	7	MW014412	3728	5	3	196.8	Group A-Rosetting/Unknown	KX154844.1	1.30E-72	Group A-Rosetting/Unknown	KX154891.1	2.19E-65	Group A-Rosetting/Unknown	HB3var1csa	2.84E-64	var1csa	KX154842.1	6.14E-61	Group A-Rosetting/Unknown	KX154869.1	1.35E-47	EPCR
	8	MW014413	3565	5	5	164.1	Group B/C	IT4var63	4.07E-43	Group B/C	HB3var47	1.48E-32	Group B/C	HB3var09	1.48E-32	Group B/C	DD2var44	6.91E-31	Group B/C	DD2var35	1.16E-28	Group B/C
	9	MW014414	3546	5	4	197.0	DC8	DD2var04	1.00E-63	Group A-Rosetting/Unknown	KX154846.1	6.06E-56	DC8	KX154941.1	3.65E-53	DC8	KX154885.1	1.71E-46	DC8	KX154897.1	2.86E-44	DC8
	10	MW014415	3532	5	4	542.2	EPCR	KX154966.1	4.02E-172	EPCR	KX154869.1	5.31E-156	EPCR	KX154967.1	1.21E-117	EPCR	KX154954.1	2.67E-99	EPCR	DD2var22	2.75E-79	Group A-Rosetting/Unknown
	11	MW014416	2244	5	5	474.9	DC8	KX154847.1	4.55E-132	DC8	KX154898.1	4.55E-132	DC8	KX154941.1	1.74E-86	DC8	KX154877.1	1.39E-67	DC8	KX154975.1	2.34E-60	DC8
	12	MW014417	2214	5	5	217.6	Group B/C	KX154819.1	6.32E-61	Group B/C	KX154880.1	1.07E-48	Group B/C	PFCLINvar66	6.50E-41	Group B/C	PFCLINvar64	2.35E-35	Group B/C	IT4var66	2.35E-35	Group B/C
	13	MW014418	1916	5	4	260.4	DC8	KX154856.1	4.46E-77	DC8	KX154941.1	4.46E-77	DC8	IT4var11	2.73E-64	Group B/C	KX154885.1	4.59E-57	DC8	KX154847.1	4.63E-52	DC8
	15	MW014419	1458	5	5	320.6	Group B/C	IT4var34	4.50E-73	Group B/C	PFCLINvar39	1.26E-68	Group B/C	PFD0630c	1.27E-63	Group B/C	KX154826.1	5.91E-62	Group B/C	PFD0635c	5.95E-57	Group B/C
	16	MW014420	1450	5	5	382.2	EPCR	KX154869.1	5.94E-91	EPCR	KX154966.1	2.14E-90	EPCR	KX154874.1	6.11E-71	EPCR	KX154967.1	1.32E-67	EPCR	KX154871.1	6.16E-66	EPCR
	18	MW014421	1047	5	5	274.5	Group B/C	IGHvar08	9.49E-60	Group B/C	PFL1960w	2.06E-56	Group B/C	DD2var34	2.66E-55	Group B/C	PFCLINvar54	3.44E-54	Group B/C	DD2var18	1.60E-52	Group B/C
	19	MW014422	1014	5	5	167.0	Group B/C	KX154970.1	2.35E-55	Group B/C	PFL0005w	3.11E-39	Group B/C	HB3var19	1.47E-27	Group B/C	RAJ116var06	8.83E-25	Group B/C	KX154828.1	1.14E-23	Group B/C
	21	MW014423	873	5	5	246.2	Group B/C	IGHvar33	5.58E-62	Group B/C	IGHvar40	4.34E-58	Group B/C	IT4var34	5.70E-47	Group B/C	IT4var23	3.43E-44	Group B/C	KX154921.1	1.24E-38	Group B/C
	22	MW014424	829	5	4	233.6	EPCR	PFCLINvar32	4.84E-62	EPCR	KX154872.1	1.74E-61	EPCR	KX154874.1	1.35E-57	EPCR	PFD0020c	1.35E-57	DC8	DD2var42	2.27E-55	EPCR
	23	MW014425	825	5	3	257.3	EPCR	IT4var02	2.05E-125	Group A-Rosetting/Unknown	KX154871.1	4.54E-107	EPCR	HB3var04	1.02E-78	Group A-Rosetting/Unknown	IT4var18	1.70E-76	EPCR	KX154893.1	6.13E-76	EPCR

	24	MW014426	822	5	3	131.5	Group B/C	KX154866.1	9.31E-50	Group B/C	PFCLINvar39	7.25E-46	Group B/C	KX154928.1	1.57E-42	DC8	KX154829.1	4.40E-38	Group B/C	IT4var32b	7.35E-36	DC8
	25	MW014427	819	5	5	185.7	Group B/C	KX154848.1	9.33E-55	Group B/C	HB3var21	2.06E-36	Group B/C	IGHvar21	3.45E-34	Group B/C	IT4var29	4.46E-33	Group B/C	IT4var32a	7.47E-31	Group B/C
	26	MW014428	698	5	5	167.4	Group B/C	KX154826.1	9.26E-44	Group B/C	IT4var33	2.61E-34	Group B/C	KX154865.1	5.65E-31	Group B/C	KX154968.1	5.65E-31	Group B/C	IT4var30	5.65E-31	Group B/C
	27	MW014429	684	5	4	188.9	Group B/C	IGHvar31	4.30E-53	Group B/C	IT4var34	1.56E-47	Group B/C	KX154831.1	7.25E-46	Group B/C	IGHvar40	2.61E-45	Group B/C	PFCLINvar30	1.59E-32	DC8
	28	MW014430	678	5	5	234.7	Group B/C	RAJ116var24	1.44E-52	Group B/C	PFC1120c	4.04E-48	Group B/C	PFL0020w	5.23E-47	Group B/C	KX154831.1	2.43E-45	Group B/C	DD2var24	2.43E-45	Group B/C
	29	MW014431	676	5	5	210.6	Group B/C	PFCLINvar66	7.51E-56	Group B/C	IGHvar35	3.54E-44	Group B/C	RAJ116var30	2.13E-41	Group B/C	RAJ116var21	1.28E-38	Group B/C	IT4var39	3.60E-34	Group B/C
	30	MW014432	673	5	5	282.3	Group B/C	IGHvar18	2.65E-70	Group B/C	PFL1955w	3.45E-64	Group B/C	IT4var34	5.81E-57	Group B/C	IT4var33	5.81E-57	Group B/C	KX154884.1	1.66E-37	Group B/C
	31	MW014433	636	5	5	235.6	Group B/C	AAC47438	9.64E-55	Group B/C	MAL7P1.56	2.09E-51	Group B/C	IT4var12	4.52E-48	Group B/C	RAJ116var36	3.52E-44	Group B/C	DD2var24	7.61E-41	Group B/C
	32	MW014434	582	5	3	274.0	EPCR	IGHvar32	2.69E-94	EPCR	IGHvar09	1.25E-92	EPCR	KX154845.1	2.71E-89	EPCR	HB3var1csa	2.73E-84	var1csa	KX154913.1	3.58E-73	Group A-Rosetting/Unknown
	33	MW014435	573	5	3	226.7	EPCR	DD2var22	2.69E-129	Group A-Rosetting/Unknown	IT4var60	2.20E-90	Group A-Rosetting/Unknown	KX154954.1	1.04E-78	EPCR	KX154936.1	6.25E-76	EPCR	KX154966.1	2.91E-74	EPCR
	35	MW014436	550	5	3	158.2	DC8	KX154825.1	6.88E-71	DC8	KX154946.1	1.95E-56	DC8	IT4var39	1.55E-37	Group B/C	DD2var49	2.60E-35	EPCR	KX154870.1	4.35E-33	DC8
	36	MW014437	541	5	4	197.0	Group B/C	PFCLINvar30	3.72E-59	DC8	IT4var30	8.06E-56	Group B/C	PFC1120c	6.27E-52	Group B/C	IT4var33	1.36E-48	Group B/C	IGHvar17	1.37E-43	Group B/C
	37	MW014438	534	5	2	128.8	EPCR	KX154876.1	1.62E-86	EPCR	PFCLINvar69	2.21E-45	Group A-Rosetting/Unknown	IGHvar23	1.03E-43	EPCR	IGHvar10	4.79E-42	Group A-Rosetting/Unknown	KX154943.1	2.24E-35	Group A-Rosetting/Unknown
	38	MW014439	528	5	4	410.4	EPCR	KX154966.1	9.53E-119	EPCR	KX154869.1	5.74E-116	EPCR	KX154967.1	1.28E-92	EPCR	KX154954.1	5.98E-86	EPCR	DD2var22	2.78E-84	Group A-Rosetting/Unknown
	39	MW014440	499	5	2	1047.4	EPCR	IGHvar39	0	EPCR	RAJ116var19	4.80E-87	Group A-Rosetting/Unknown	ACJ68328	3.85E-63	Group A-Rosetting/Unknown	KX154899.1	1.82E-51	Group A-Rosetting/Unknown	KX154874.1	3.93E-48	EPCR
	41	MW014441	428	5	5	285.8	Group B/C	HB3var33	8.84E-75	Group B/C	DD2var18	1.51E-57	Group B/C	RAJ116var36	3.27E-54	Group B/C	KX154922.1	1.18E-53	Group B/C	HB3var40	3.29E-49	Group B/C
	42	MW014442	410	5	5	198.1	Group B/C	KX154860.1	5.18E-47	Group B/C	PFCLINvar66	1.45E-42	Group B/C	DD2var16	4.06E-38	Group B/C	PFCLINvar64	5.25E-37	Group B/C	IT4var66	5.25E-37	Group B/C
	43	MW014443	391	5	4	359.2	EPCR	IT4var07	5.99E-106	EPCR	PF11_0521	4.69E-97	EPCR	IT4var08	2.20E-90	Group A-Rosetting/Unknown	KX154945.1	1.02E-88	EPCR	KX154903.1	2.26E-70	EPCR
	45	MW014444	378	5	5	217.0	Group B/C	PFCLINvar52	1.11E-53	Group B/C	KX154907.1	2.41E-45	Group B/C	HB3var08	2.41E-45	Group B/C	IT4var01	2.43E-40	Group B/C	PFL0020w	6.80E-36	Group B/C
	46	MW014445	361	5	5	147.8	Group B/C	DD2var38	4.54E-33	Group B/C	IGHvar34	5.87E-32	Group B/C	RAJ116var39	2.11E-31	Group B/C	DD2var39	2.11E-31	Group B/C	IT4var67	1.28E-23	Group B/C
	47	MW014446	347	5	5	288.9	Group B/C	PFCLINvar39	4.57E-73	Group B/C	KX154955.1	7.65E-71	Group B/C	PF13_0364	1.30E-58	Group B/C	IT4var34	1.70E-47	Group B/C	IT4var62	1.72E-42	Group B/C
	48	MW014447	347	5	5	269.0	Group B/C	IGHvar33	1.19E-68	Group B/C	IGHvar40	5.58E-62	Group B/C	KX154950.1	1.22E-53	Group B/C	IT4var33	9.54E-45	Group B/C	KX154906.1	1.23E-43	Group B/C
40	1	MW014448	14222	5	5	869.1	var1csa	DD2var25	2.50E-179	var1csa	AJ420411	2.50E-179	var1csa	KX154917.1	4.19E-177	var1csa	IGHvar11	2.54E-169	var1csa	AAM55194	1.18E-167	var1csa
	2	MW014449	13955	5	5	274.5	Group B/C	IGHvar08	9.49E-60	Group B/C	PFL1960w	2.06E-56	Group B/C	DD2var34	2.66E-55	Group B/C	PFCLINvar54	3.44E-54	Group B/C	DD2var18	1.60E-52	Group B/C
	3	MW014450	12986	5	3	105.5	Group B/C	IT4var32b	1.91E-46	DC8	IT4var19	1.91E-46	DC8	RAJ116var10	6.98E-36	Group B/C	IT4var41	6.98E-36	Group B/C	DD2var39	6.98E-36	Group B/C
	4	MW014451	9076	5	5	164.1	Group B/C	IT4var63	4.07E-43	Group B/C	HB3var47	1.48E-32	Group B/C	HB3var09	1.48E-32	Group B/C	DD2var44	6.91E-31	Group B/C	DD2var35	1.16E-28	Group B/C
	5	MW014452	5526	5	3	131.5	Group B/C	KX154866.1	9.31E-50	Group B/C	PFCLINvar39	7.25E-46	Group B/C	KX154928.1	1.57E-42	DC8	KX154829.1	4.40E-38	Group B/C	IT4var32b	7.35E-36	DC8
	6	MW014453	5022	5	5	217.6	Group B/C	KX154819.1	6.32E-61	Group B/C	KX154880.1	1.07E-48	Group B/C	PFCLINvar66	6.50E-41	Group B/C	PFCLINvar64	2.35E-35	Group B/C	IT4var66	2.35E-35	Group B/C
	7	MW014454	4892	5	3	158.2	DC8	KX154825.1	6.88E-71	DC8	KX154946.1	1.95E-56	DC8	IT4var39	1.55E-37	Group B/C	DD2var49	2.60E-35	EPCR	KX154870.1	4.35E-33	DC8
	8	MW014455	4598	5	5	319.8	Group B/C	RAJ116var35	2.36E-80	Group B/C	PFCLINvar61	2.36E-80	Group B/C	IT4var40	1.87E-61	Group B/C	RAJ116var33	1.90E-51	Group B/C	PF07_0050	8.84E-50	Group B/C
	9	MW014456	4170	5	5	320.6	Group B/C	IT4var34	4.50E-73	Group B/C	PFCLINvar39	1.26E-68	Group B/C	PFD0630c	1.27E-63	Group B/C	KX154826.1	5.91E-62	Group B/C	PFD0635c	5.95E-57	Group B/C
	10	MW014457	3791	5	5	185.7	Group B/C	KX154848.1	9.33E-55	Group B/C	HB3var21	2.06E-36	Group B/C	IGHvar21	3.45E-34	Group B/C	IT4var29	4.46E-33	Group B/C	IT4var32a	7.47E-31	Group B/C

	11	MW014458	3507	5	5	327.9	Group B/C	IT4var33	9.31E-85	Group B/C	PFL1955w	2.06E-66	Group B/C	KX154819.1	5.77E-62	Group B/C	IGHvar31	9.65E-60	Group B/C	KX154910.1	1.25E-58	Group B/C
	12	MW014459	3178	5	5	227.6	Group B/C	PFL0005w	6.17E-91	Group B/C	RAJ116var06	1.40E-52	Group B/C	PF08_0103	5.19E-32	Group B/C	HB3var16	3.13E-29	Group B/C	PFC0005w	1.88E-26	Group B/C
	13	MW014460	2689	5	5	210.6	Group B/C	PFCLINvar66	7.51E-56	Group B/C	IGHvar35	3.54E-44	Group B/C	RAJ116var30	2.13E-41	Group B/C	RAJ116var21	1.28E-38	Group B/C	IT4var39	3.60E-34	Group B/C
	14	MW014461	1622	5	5	557.9	EPCR	KX154954.1	4.15E-157	EPCR	KX154967.1	4.39E-117	EPCR	KX154869.1	4.45E-107	EPCR	KX154966.1	5.76E-106	EPCR	IGHvar09	2.80E-74	EPCR
	15	MW014462	1093	5	5	194.3	Group B/C	KX154884.1	2.98E-49	Group B/C	PFL1955w	6.50E-41	Group B/C	DD2var26	3.91E-38	Group B/C	IT4var32a	8.47E-35	Group B/C	IT4var14	8.47E-35	Group B/C
	16	MW014463	1075	5	5	1226.2	Group B/C	IGHvar29	0	Group B/C	IGHvar17	1.16E-68	Group B/C	RAJ116var09	5.44E-62	Group B/C	PFL2665c	1.99E-51	Group B/C	KX154896.1	5.56E-47	Group B/C
	17	MW014464	951	5	5	246.2	Group B/C	IGHvar33	5.58E-62	Group B/C	IGHvar40	4.34E-58	Group B/C	IT4var34	5.70E-47	Group B/C	IT4var23	3.43E-44	Group B/C	KX154921.1	1.24E-38	Group B/C
	18	MW014465	735	5	3	90.4	Group B/C	PFCLINvar30	3.05E-84	DC8	PFCLINvar69	1.97E-31	Group A-Rosetting/Unknown	DD2var38	1.97E-31	Group B/C	DD2var12	7.09E-31	Group B/C	IT4var67	2.55E-30	Group B/C
	19	MW014466	728	5	5	217.0	Group B/C	PFCLINvar52	1.11E-53	Group B/C	KX154907.1	2.41E-45	Group B/C	HB3var08	2.41E-45	Group B/C	IT4var01	2.43E-40	Group B/C	PFL0020w	6.80E-36	Group B/C
	20	MW014467	728	5	3	305.7	EPCR	KX154845.1	2.14E-110	EPCR	IGHvar09	2.15E-105	EPCR	IGHvar32	4.73E-92	EPCR	DD2var22	2.20E-90	Group A-Rosetting/Unknown	IT4var60	1.05E-73	Group A-Rosetting/Unknown
	21	MW014468	703	5	4	182.3	Group B/C	IGHvar20	8.36E-90	Group B/C	IGHvar01	3.18E-49	Group B/C	RAJ116var33	7.08E-26	Group B/C	PFCLINvar31	2.55E-25	DC8	KX154916.1	2.56E-20	Group B/C
	22	MW014469	678	5	3	262.3	EPCR	IT4var02	4.41E-127	Group A-Rosetting/Unknown	KX154871.1	9.76E-109	EPCR	HB3var04	2.19E-80	Group A-Rosetting/Unknown	IT4var18	3.66E-78	EPCR	KX154893.1	1.32E-77	EPCR
	23	MW014470	658	5	5	648.5	EPCR	IGHvar27	2.40E-169	EPCR	KX154935.1	8.62E-169	EPCR	HB3var03	5.34E-146	EPCR	KX154918.1	5.73E-96	EPCR	KX154901.1	4.59E-72	EPCR
	25	MW014471	593	5	5	218.5	Group B/C	HB3var28	1.55E-57	Group B/C	IGHvar40	5.65E-47	Group B/C	RAJ116var30	9.53E-40	Group B/C	PFCLINvar11	3.43E-39	Group B/C	IT4var05	1.23E-38	Group B/C
	26	MW014472	536	5	5	193.3	Group B/C	DD2var45	7.70E-61	Group B/C	HB3var30	1.71E-37	Group B/C	HB3var18	2.87E-35	Group B/C	MAL8P1.220	6.21E-32	Group B/C	PFD0005w	2.23E-31	Group B/C
	27	MW014473	522	5	5	218.8	Group B/C	DD2var18	1.34E-52	Group B/C	RAJ116var36	1.74E-46	Group B/C	AY028643	4.88E-42	Group B/C	IT4var17	6.32E-41	Group B/C	PFD1000c	2.27E-40	Group B/C
	28	MW014474	485	5	3	196.8	Group A-Rosetting/Unknown	KX154844.1	1.30E-72	Group A-Rosetting/Unknown	KX154891.1	2.19E-65	Group A-Rosetting/Unknown	HB3var1csa	2.84E-64	var1csa	KX154842.1	6.14E-61	Group A-Rosetting/Unknown	KX154869.1	1.35E-47	EPCR
	29	MW014475	437	5	5	317.4	Group B/C	PFL1950w	4.08E-103	Group B/C	HB3var23	4.08E-103	Group B/C	IGHvar17	3.43E-44	Group B/C	PFCLINvar43	7.48E-36	Group B/C	DD2var44	9.67E-35	Group B/C
	30	MW014476	433	5	5	204.5	Group B/C	KX154969.1	4.44E-48	Group B/C	PFL2665c	7.44E-46	Group B/C	KX154884.1	1.61E-42	Group B/C	DD2var51	7.54E-36	Group B/C	DD2var24	7.54E-36	Group B/C
	32	MW014477	430	5	5	257.5	Group B/C	IGHvar28	2.32E-90	Group B/C	PFCLINvar04	2.42E-60	Group B/C	IT4var15	2.49E-40	Group B/C	DD2var13	2.49E-40	Group B/C	PFA0005w	9.10E-30	Group B/C
46	1	MW014478	12876	5	2	163.3	Group A-Rosetting/Unknown	KX154943.1	9.49E-94	Group A-Rosetting/Unknown	KX154978.1	5.88E-71	Group A-Rosetting/Unknown	PFCLINvar73	9.83E-69	EPCR	KX154863.1	1.01E-48	Group B/C	PFCLINvar71	4.71E-47	Group B/C
	2	MW014479	9208	5	3	109.3	Group B/C	KX154936.1	1.51E-47	EPCR	KX154928.1	2.52E-45	DC8	KX154821.1	1.96E-41	Group B/C	HB3var21	7.11E-36	Group B/C	IGHvar37	3.31E-34	Group B/C
	3	MW014480	8808	5	5	553.4	EPCR	KX154890.1	4.44E-122	EPCR	KX154923.1	9.62E-119	EPCR	KX154937.1	9.62E-119	EPCR	KX154857.1	4.54E-107	EPCR	KX154909.1	2.16E-90	EPCR
	4	MW014481	7089	5	5	472.3	DC8	KX154975.1	5.51E-116	DC8	KX154941.1	5.59E-106	DC8	KX154856.1	2.64E-94	DC8	KX154925.1	4.42E-92	DC8	KX154846.1	1.27E-67	DC8
	5	MW014482	6956	5	5	270.4	Group B/C	HB3var34	1.37E-77	Group B/C	RAJ116var37	4.99E-67	Group B/C	IGHvar02	6.50E-61	Group B/C	KX154841.1	1.12E-38	Group B/C	IT4var40	8.77E-30	Group B/C
	6	MW014483	5180	5	3	142.2	Group B/C	KX154825.1	1.11E-68	DC8	KX154946.1	5.16E-67	DC8	DD2var18	1.88E-56	Group B/C	PFCLINvar66	2.47E-45	Group B/C	PFL0935c	1.49E-42	Group B/C
	7	MW014484	4234	5	5	197.9	Group B/C	HB3var07	5.51E-47	Group B/C	KX154821.1	2.56E-45	Group B/C	KX154819.1	9.29E-40	Group B/C	KX154815.1	9.35E-35	Group B/C	HB3var16	9.35E-35	Group B/C
	8	MW014485	3998	5	5	338.7	Group B/C	HB3var08	1.38E-97	Group B/C	KX154907.1	6.60E-76	Group B/C	IT4var32a	1.13E-58	Group B/C	IT4var14	1.13E-58	Group B/C	RAJ116var39	1.90E-51	Group B/C
	9	MW014486	3992	5	4	142.8	Group B/C	AAB60251	7.30E-41	Group B/C	PFCLINvar30	1.58E-37	DC8	KX154969.1	2.04E-36	Group B/C	KX154853.1	3.42E-34	Group B/C	RAJ116var24	3.42E-34	Group B/C
	10	MW014487	3807	5	5	451.5	Group B/C	DD2var36	1.01E-123	Group B/C	MAL6P1.252	6.08E-121	Group B/C	PFCLINvar60	6.21E-106	Group B/C	HB3var18	3.08E-59	Group B/C	DD2var38	2.43E-45	Group B/C
	11	MW014488	3699	5	4	231.3	Group B/C	PFL0005w	7.79E-120	Group B/C	IT4var67	2.43E-40	Group B/C	RAJ116var06	8.73E-40	Group B/C	HB3var16	3.16E-34	Group B/C	KX154964.1	1.47E-32	DC8
	12	MW014489	3246	5	4	211.2	Group B/C	RAJ116var24	2.32E-90	Group B/C	RAJ116var29	1.46E-57	Group B/C	HB3var11	4.17E-38	Group B/C	PFCLINvar30	1.17E-33	DC8	KX154868.1	4.23E-28	Group B/C

	13	MW014490	3053	5	3	105.5	Group B/C	IT4var32b	1.91E-46	DC8	IT4var19	1.91E-46	DC8	RAJ116var10	6.98E-36	Group B/C	IT4var41	6.98E-36	Group B/C	DD2var39	6.98E-36	Group B/C
	14	MW014491	2608	5	5	227.0	Group B/C	PF07_0139	2.35E-50	Group B/C	PF08_0107	3.93E-48	Group B/C	PF07_0048	6.58E-46	Group B/C	MAL7P1.212	1.10E-43	Group B/C	PF08_0106	1.42E-42	Group B/C
	15	MW014492	2392	5	5	540.1	EPCR	KX154949.1	1.25E-112	EPCR	AAQ73928	1.25E-112	EPCR	KX154875.1	4.51E-112	EPCR	KX154879.1	4.51E-112	EPCR	KX154918.1	2.77E-94	EPCR
	16	MW014493	1812	5	4	299.1	EPCR	IT4var60	6.03E-101	Group A-Rosetting/Unknown	KX154874.1	1.69E-96	EPCR	PFCLINvar62	1.31E-92	EPCR	DD2var40	1.79E-56	EPCR	DD2var09a	1.79E-56	EPCR
	17	MW014494	1696	5	5	167.8	Group B/C	IGHvar01	1.27E-48	Group B/C	PFCLINvar66	4.62E-38	Group B/C	IGHvar35	1.01E-29	Group B/C	PFCLINvar54	1.68E-27	Group B/C	DD2var11	1.68E-27	Group B/C
	18	MW014495	1547	5	4	535.5	EPCR	KX154966.1	8.77E-164	EPCR	KX154869.1	5.28E-161	EPCR	KX154967.1	5.62E-116	EPCR	KX154954.1	1.24E-97	EPCR	DD2var22	5.95E-76	Group A-Rosetting/Unknown
	19	MW014496	1507	5	5	211.9	Group B/C	KX154912.1	3.21E-49	Group B/C	IGHvar25	1.93E-46	Group B/C	PFCLINvar29	1.16E-43	Group B/C	KX154921.1	1.50E-42	Group B/C	PFC0005w	1.18E-33	Group B/C
	20	MW014497	1324	5	5	869.1	var1csa	DD2var25	2.50E-179	var1csa	AJ420411	2.50E-179	var1csa	KX154917.1	4.19E-177	var1csa	IGHvar11	2.54E-169	var1csa	AAM55194	1.18E-167	var1csa
59	1	MW014498	21625	5	5	302.8	Group B/C	KX154940.1	2.74E-74	Group B/C	IT4var26	1.28E-67	Group B/C	HB3var14	7.79E-60	Group B/C	HB3var40	2.82E-54	Group B/C	DD2var44	2.20E-50	Group B/C
	2	MW014499	17266	5	5	306.8	Group B/C	PFCLINvar56	4.39E-73	Group B/C	PFD0625c	7.41E-66	Group B/C	IGHvar13	2.66E-65	Group B/C	PFD1015c	9.72E-55	Group B/C	HB3var50	2.10E-51	Group B/C
	3	MW014500	10221	5	4	211.4	Group B/C	KX154972.1	2.15E-65	Group B/C	IT4var11	2.16E-60	Group B/C	IT4var13	2.86E-44	Group B/C	DD2var21	2.86E-44	Group B/C	KX154947.1	4.85E-32	DC8
	4	MW014501	8349	5	5	215.7	Group B/C	KX154852.1	9.25E-55	Group B/C	IT4var54	7.25E-46	Group B/C	KX154912.1	7.30E-41	Group B/C	KX154828.1	9.45E-40	Group B/C	TM284S2var1	4.40E-38	Group B/C
	5	MW014502	8056	5	3	158.4	EPCR	KX154872.1	2.88E-59	EPCR	IGHvar24	1.04E-58	Group A-Rosetting/Unknown	RAJ116var17	1.34E-57	Group A-Rosetting/Unknown	KX154818.1	1.35E-52	EPCR	HB3var02	1.05E-48	EPCR
	6	MW014503	7892	5	5	506.7	EPCR	KX154949.1	5.87E-106	EPCR	KX154875.1	2.11E-105	EPCR	KX154879.1	2.11E-105	EPCR	AAQ73928	1.27E-102	EPCR	KX154818.1	6.00E-91	EPCR
	7	MW014504	6682	5	3	258.4	EPCR	KX154926.1	2.74E-109	EPCR	IGHvar24	2.21E-80	Group A-Rosetting/Unknown	KX154890.1	1.33E-77	EPCR	KX154874.1	1.04E-73	EPCR	PF13_0003	4.82E-72	Group A-Rosetting/Unknown
	8	MW014505	6374	5	3	104.9	EPCR	PFD1235w	3.20E-44	EPCR	MAL7P1.1	3.20E-44	Group B/C	KX154888.1	1.95E-31	EPCR	KX154903.1	1.95E-31	EPCR	ACJ68328	4.26E-23	Group A-Rosetting/Unknown
	9	MW014506	6121	5	3	202.3	var1csa	DD2var32	4.47E-97	EPCR	HB3var02	9.89E-79	EPCR	KX154820.1	3.61E-68	var1csa	KX154858.1	3.61E-68	var1csa	KX154932.1	3.61E-68	var1csa
	10	MW014507	5083	5	5	249.6	Group B/C	KX154821.1	1.20E-53	Group B/C	KX154912.1	1.55E-52	Group B/C	AY028643	7.20E-51	Group B/C	IT4var61	1.20E-48	Group B/C	DD2var31	1.56E-47	Group B/C
	11	MW014508	4522	5	3	179.5	Group A-Rosetting/Unknown	PF13_0003	1.34E-77	Group A-Rosetting/Unknown	KX154926.1	4.93E-62	EPCR	DD2var42	2.31E-55	EPCR	KX154899.1	1.39E-52	Group A-Rosetting/Unknown	IT4var02	1.80E-51	Group A-Rosetting/Unknown
	12	MW014509	3551	5	4	217.3	Group B/C	KX154882.1	2.39E-70	Group B/C	PFA0005w	6.79E-56	Group B/C	HB3var24	2.46E-50	Group B/C	KX154825.1	1.15E-43	DC8	DD2var16	1.15E-43	Group B/C
	13	MW014510	3205	5	5	327.7	Group B/C	RAJ116var14	6.23E-71	Group B/C	PF10_0406	4.85E-67	Group B/C	KX154862.1	1.74E-66	Group B/C	IGHvar38	8.12E-65	Group B/C	IGHvar25	4.89E-62	Group B/C
	14	MW014511	3177	5	5	242.4	Group B/C	KX154971.1	9.17E-55	Group B/C	RAJ116var36	1.53E-52	Group B/C	DD2var24	1.19E-48	Group B/C	MAL7P1.56	2.59E-45	Group B/C	IT4var05	9.30E-45	Group B/C
	15	MW014512	2378	5	3	183.9	EPCR	PFCLINvar62	2.95E-69	EPCR	KX154959.1	2.30E-65	EPCR	IGHvar12	2.33E-55	Group A-Rosetting/Unknown	HB3var05	2.33E-55	Group A-Rosetting/Unknown	KX154936.1	1.82E-51	EPCR
	16	MW014513	2178	5	5	250.5	Group B/C	IT4var23	1.28E-63	Group B/C	PFCLINvar66	2.17E-51	Group B/C	KX154971.1	1.01E-49	Group B/C	PFCLINvar65	1.02E-44	Group B/C	IT4var59	1.02E-44	Group B/C
	17	MW014514	1915	5	3	119.9	Group B/C	KX154958.1	4.05E-53	EPCR	IGHvar17	3.17E-44	Group B/C	DD2var35	8.89E-40	Group B/C	HB3var29	4.14E-38	Group B/C	KX154825.1	5.35E-37	DC8
	18	MW014515	1827	5	5	286.9	Group B/C	DD2var23	8.75E-70	Group B/C	KX154912.1	1.47E-62	Group B/C	PF10_0406	1.91E-61	Group B/C	RAJ116var06	5.38E-52	Group B/C	KX154815.1	9.06E-45	Group B/C
	19	MW014516	1744	5	4	239.8	Group B/C	KX154972.1	3.54E-68	Group B/C	PFCLINvar09	3.54E-68	Group B/C	PFCLINvar71	5.92E-66	Group B/C	KX154856.1	6.13E-41	DC8	PFD1015c	2.20E-40	Group B/C
	20	MW014517	1673	5	5	279.6	Group B/C	KX154963.1	5.50E-72	Group B/C	AAB60251	4.28E-68	Group B/C	DD2var50	7.27E-56	Group B/C	IGHvar29	1.23E-43	Group B/C	IGHvar17	1.23E-43	Group B/C
	21	MW014518	1646	5	5	177.1	Group B/C	PF07_0048	4.94E-47	Group B/C	PF08_0106	5.01E-37	Group B/C	PF08_0107	1.09E-33	Group B/C	PFCLINvar43	5.05E-32	Group B/C	PFCLINvar23	6.53E-31	Group B/C
	22	MW014519	1578	5	4	171.6	Group B/C	PFCLINvar66	1.20E-63	Group B/C	HB3var30	7.37E-46	Group B/C	KX154826.1	1.62E-32	Group B/C	PFD1245c	1.62E-32	Group B/C	PFCLINvar30	1.62E-32	DC8
	23	MW014520	1433	5	5	805.2	var1csa	KX154974.1	1.94E-180	var1csa	AAM55194	1.20E-157	var1csa	TM284var3	1.20E-157	var1csa	KX154917.1	4.31E-157	var1csa	IGHvar11	5.58E-156	var1csa

	24	MW014521	1432	5	5	207.0	Group B/C	KX154868.1	3.95E-58	Group B/C	KX154956.1	1.11E-48	Group B/C	PFI1830c	1.13E-38	Group B/C	DD2var18	4.09E-33	Group B/C	KX154832.1	5.29E-32	Group B/C
	25	MW014522	922	5	5	246.3	Group B/C	AY028643	1.49E-62	Group B/C	IT4var39	4.19E-53	Group B/C	IGHvar40	1.97E-46	Group B/C	PFL2665c	2.54E-45	Group B/C	PFA0005w	1.53E-42	Group B/C
	26	MW014523	888	5	3	261.2	EPCR	KX154927.1	9.99E-99	EPCR	DD2var49	2.18E-90	EPCR	HB3var1csa	6.19E-76	var1csa	DD2var43	2.88E-74	EPCR	AAM55194	1.38E-52	var1csa
	27	MW014524	833	5	3	249.0	EPCR	KX154926.1	2.12E-115	EPCR	PF13_0003	2.91E-74	Group A-Rosetting/Unknown	KX154890.1	1.05E-68	EPCR	KX154959.1	4.90E-67	EPCR	KX154899.1	2.95E-64	Group A-Rosetting/Unknown
74	1	MW014525	48746	5	3	182.2	Group B/C	PFCLINvar06	2.31E-95	Group B/C	DD2var07	8.90E-45	Group B/C	DD2var41	3.20E-44	Group B/C	IT4var03	8.97E-40	Type 3 var	KX154825.1	1.52E-27	DC8
	2	MW014526	10493	5	5	204.7	Group B/C	PFCLINvar45	2.42E-55	Group B/C	HB3var11	4.11E-43	Group B/C	HB3var19	1.91E-41	Group B/C	KX154848.1	5.35E-37	Group B/C	PFB1055c	1.94E-31	Group B/C
	3	MW014527	8059	5	5	363.9	EPCR	KX154918.1	2.70E-99	EPCR	KX154875.1	4.71E-67	EPCR	KX154879.1	4.71E-67	EPCR	KX154949.1	4.71E-67	EPCR	AAQ73928	4.71E-67	EPCR
	4	MW014528	7514	5	5	245.5	Group B/C	PFCLINvar71	2.16E-55	Group B/C	KX154894.1	1.30E-52	Group B/C	PFD1015c	2.17E-50	Group B/C	RAJ116var22	7.82E-50	Group B/C	KX154864.1	6.13E-41	Group B/C
	5	MW014529	5418	5	2	122.1	Group A-Rosetting/Unknown	IGHvar22	1.05E-73	Group A-Rosetting/Unknown	IGHvar24	8.45E-50	Group A-Rosetting/Unknown	KX154926.1	5.12E-42	EPCR	IT4var22	5.12E-42	EPCR	DQ408104	5.12E-42	Type 3 var
	6	MW014530	4645	5	4	335.8	EPCR	PFCLINvar62	9.95E-109	EPCR	IT4var60	1.70E-91	Group A-Rosetting/Unknown	KX154924.1	1.33E-82	EPCR	KX154874.1	1.74E-76	EPCR	KX154830.1	6.29E-71	EPCR
	7	MW014531	3129	5	5	236.7	Group B/C	DD2var39	7.29E-66	Group B/C	RAJ116var39	9.43E-65	Group B/C	IGHvar16	2.10E-41	Group B/C	PFD1000c	3.54E-34	Group B/C	PFD0995c	3.54E-34	Group B/C
	8	MW014532	3093	5	5	423.9	EPCR	KX154936.1	2.77E-94	EPCR	KX154871.1	7.76E-90	EPCR	KX154966.1	1.01E-83	EPCR	KX154869.1	1.69E-81	EPCR	KX154967.1	3.66E-78	EPCR
	9	MW014533	2843	5	3	164.4	Group B/C	HB3var22	1.82E-81	Group B/C	IGHvar29	4.11E-48	Group B/C	KX154930.1	1.93E-41	EPCR	KX154892.1	1.16E-38	DC8	KX154971.1	5.40E-37	Group B/C
	10	MW014534	2818	5	5	203.1	Group B/C	KX154894.1	8.19E-65	Group B/C	IT4var41	1.39E-52	Group B/C	RAJ116var39	6.66E-31	Group B/C	KX154905.1	2.39E-30	Group B/C	HB3var16	4.01E-28	Group B/C
	11	MW014535	1769	5	5	295.2	Group B/C	KX154910.1	2.52E-75	Group B/C	KX154955.1	1.18E-68	Group B/C	IGHvar18	3.33E-59	Group B/C	PFL1955w	7.26E-51	Group B/C	HB3var24	9.46E-45	Group B/C
	12	MW014536	1691	5	5	277.0	Group B/C	IT4var15	8.48E-60	Group B/C	DD2var13	8.48E-60	Group B/C	PFD1005c	3.05E-59	Group B/C	MAL6P1.1	6.65E-51	Group B/C	HB3var26	6.65E-51	Group B/C
	13	MW014537	1249	5	5	849.2	var1csa	RAJ116var02	5.15E-171	var1csa	PFCLINvar76	5.15E-171	var1csa	AF547159	5.15E-171	var1csa	KX154858.1	1.85E-170	var1csa	KX154820.1	2.40E-169	var1csa
	14	MW014538	1091	5	2	1088.0	Group A-Rosetting/Unknown	DD2var22	0	Group A-Rosetting/Unknown	IT4var60	1.02E-88	Group A-Rosetting/Unknown	KX154871.1	4.76E-87	EPCR	KX154936.1	6.20E-81	EPCR	KX154966.1	2.89E-79	EPCR
	15	MW014539	911	5	5	163.0	Group B/C	KX154921.1	4.18E-43	Group B/C	HB3var07	2.53E-35	Group B/C	PFL0935c	9.18E-30	Group B/C	PFC0005w	3.30E-29	Group B/C	DD2var33	3.30E-29	Group B/C
	16	MW014540	882	5	5	265.5	Group B/C	KX154894.1	1.25E-82	Group B/C	PFCLINvar71	1.30E-52	Group B/C	PFD1015c	2.17E-50	Group B/C	IT4var41	3.66E-43	Group B/C	KX154972.1	2.20E-40	Group B/C
	17	MW014541	735	5	5	254.8	Group B/C	PFCLINvar44	3.96E-63	Group B/C	DD2var46	3.96E-63	Group B/C	KX154854.1	6.71E-51	Group B/C	KX154839.1	6.81E-41	Group B/C	PFCLINvar23	2.45E-40	Group B/C
78	1	MW014542	35435	5	4	380.9	EPCR	KX154967.1	2.04E-115	EPCR	KX154954.1	4.42E-112	EPCR	RAJ116var05	1.27E-87	Group A-Rosetting/Unknown	KX154966.1	5.97E-81	EPCR	KX154869.1	2.16E-75	EPCR
	2	MW014543	11548	5	5	239.0	Group B/C	PFCLINvar08	3.29E-79	Group B/C	IT4var36	2.66E-50	Group B/C	KX154905.1	9.69E-40	Group B/C	HB3var26	1.62E-37	Group B/C	KX154866.1	7.54E-36	Group B/C
	3	MW014544	8735	5	3	258.4	EPCR	KX154926.1	2.74E-109	EPCR	IGHvar24	2.21E-80	Group A-Rosetting/Unknown	KX154890.1	1.33E-77	EPCR	KX154874.1	1.04E-73	EPCR	PF13_0003	4.82E-72	Group A-Rosetting/Unknown
	4	MW014545	8680	5	3	188.4	Group A-Rosetting/Unknown	KX154843.1	2.87E-69	Group A-Rosetting/Unknown	IT4var09	1.35E-62	Group A-Rosetting/Unknown	IGHvar39	1.35E-62	EPCR	KX154845.1	4.84E-62	EPCR	RAJ116var19	1.05E-58	Group A-Rosetting/Unknown
	5	MW014546	5340	5	5	424.5	DC8	KX154878.1	4.38E-97	DC8	KX154914.1	4.38E-97	DC8	KX154961.1	4.38E-97	DC8	KX154856.1	2.70E-79	DC8	KX154975.1	1.29E-57	DC8
	6	MW014547	3596	5	5	306.5	Group B/C	KX154862.1	2.99E-79	Group B/C	KX154851.1	1.09E-68	Group B/C	RAJ116var06	8.56E-60	Group B/C	IGHvar25	6.67E-56	Group B/C	MAL7P1.55	1.88E-46	Group B/C
	7	MW014548	3418	5	4	350.3	EPCR	KX154926.1	5.74E-116	EPCR	PF13_0003	4.66E-82	Group A-Rosetting/Unknown	KX154954.1	7.80E-80	EPCR	KX154890.1	2.80E-79	EPCR	KX154967.1	3.63E-78	EPCR
	8	MW014549	3233	5	5	249.7	Group B/C	KX154852.1	8.53E-75	Group B/C	TM284S2var1	6.79E-56	Group B/C	IT4var61	1.15E-43	Group B/C	RAJ116var24	2.49E-40	Group B/C	IGHvar21	1.16E-38	Group B/C
	9	MW014550	2624	5	5	304.0	Group B/C	IT4var54	1.55E-82	Group B/C	RAJ116var21	5.64E-72	Group B/C	IGHvar36	2.06E-61	Group B/C	IGHvar08	7.50E-51	Group B/C	MAL7P1.50	7.61E-41	Group B/C
	10	MW014551	2601	5	5	274.7	Group B/C	MAL7P1.56	1.88E-66	Group B/C	PFCLINvar28	3.16E-59	Group B/C	PFC1120c	1.15E-53	Group B/C	KX154900.1	8.92E-50	Group B/C	IT4var61	3.21E-49	Group B/C

	11	MW014552	2537	5	3	202.3	var1csa	DD2var32	4.47E-97	EPCR	HB3var02	9.89E-79	EPCR	KX154820.1	3.61E-68	var1csa	KX154858.1	3.61E-68	var1csa	KX154932.1	3.61E-68	var1csa
	12	MW014553	2171	5	4	285.8	EPCR	KX154843.1	2.81E-84	Group A-Rosetting/Unknown	KX154966.1	2.83E-79	EPCR	KX154869.1	4.77E-72	EPCR	KX154954.1	1.03E-68	EPCR	KX154967.1	1.03E-68	EPCR
	13	MW014554	2142	5	5	281.2	Group B/C	PFI1830c	5.59E-72	Group B/C	PFCLINvar66	7.24E-71	Group B/C	KX154819.1	7.44E-51	Group B/C	KX154950.1	1.25E-48	Group B/C	IT4var61	1.62E-42	Group B/C
	14	MW014555	1762	5	3	209.5	EPCR	RAJ116var05	2.17E-85	Group A-Rosetting/Unknown	KX154966.1	2.85E-74	EPCR	RAJ116var17	1.03E-73	Group A-Rosetting/Unknown	KX154869.1	1.03E-68	EPCR	KX154967.1	1.03E-68	EPCR
	15	MW014556	1757	5	5	1499.1	var1csa	AAM55194	0	var1csa	TM284var3	0	var1csa	IGHvar11	2.54E-169	var1csa	DD2var25	5.50E-166	var1csa	AJ420411	5.50E-166	var1csa
	16	MW014557	1673	5	3	205.6	Group A-Rosetting/Unknown	KX154915.1	2.21E-80	Group A-Rosetting/Unknown	IGHvar22	2.23E-75	Group A-Rosetting/Unknown	DD2var40	2.96E-54	EPCR	DD2var09a	2.96E-54	EPCR	KX154842.1	4.95E-52	Group A-Rosetting/Unknown
	17	MW014558	1647	5	2	173.2	EPCR	KX154876.1	5.63E-136	EPCR	PFCLINvar69	2.28E-50	Group A-Rosetting/Unknown	IGHvar23	1.08E-38	EPCR	IGHvar06	1.41E-27	Group B/C	RAJ116var19	5.08E-27	Group A-Rosetting/Unknown
	18	MW014559	1448	5	5	197.6	Group B/C	HB3var12	6.24E-56	Group B/C	HB3var40	1.76E-46	Group B/C	HB3var14	6.33E-46	Group B/C	PF08_0103	3.87E-33	Group B/C	PFCLINvar06	8.49E-20	Group B/C
	19	MW014560	1228	5	5	388.3	EPCR	KX154954.1	9.78E-94	EPCR	KX154967.1	4.55E-92	EPCR	IGHvar09	6.01E-76	EPCR	IGHvar32	1.30E-72	EPCR	KX154966.1	1.33E-57	EPCR
	20	MW014561	1188	5	5	245.7	Group B/C	PFCLINvar66	1.50E-67	Group B/C	KX154831.1	1.53E-52	Group B/C	PFCLINvar64	5.56E-47	Group B/C	IT4var66	5.56E-47	Group B/C	IT4var45	2.62E-35	Group B/C
	21	MW014562	1116	5	3	129.5	Group B/C	KX154864.1	1.01E-48	Group B/C	MAL6P1.316	1.01E-48	DC8	KX154942.1	3.64E-48	DC8	IGHvar05	2.83E-44	Group B/C	PFCLINvar09	1.03E-38	Group B/C
	22	MW014563	955	5	3	363.6	DC8	KX154873.1	1.85E-170	DC8	KX154947.1	7.25E-110	DC8	KX154941.1	2.09E-85	DC8	HB3var03	2.74E-74	EPCR	IGHvar27	5.93E-71	EPCR
	23	MW014564	860	5	5	185.3	Group B/C	KX154907.1	1.44E-42	Group B/C	PFL0020w	6.69E-41	Group B/C	HB3var08	5.21E-37	Group B/C	KX154831.1	2.42E-35	Group B/C	PFCLINvar52	4.05E-33	Group B/C
	24	MW014565	737	5	3	267.3	EPCR	KX154926.1	5.89E-111	EPCR	KX154843.1	4.68E-92	Group A-Rosetting/Unknown	KX154890.1	6.14E-81	EPCR	RAJ116var19	6.14E-81	Group A-Rosetting/Unknown	KX154923.1	1.33E-77	EPCR
	25	MW014566	698	5	5	287.8	Group B/C	IT4var62	1.58E-77	Group B/C	IGHvar13	3.50E-59	Group B/C	RAJ116var28	5.85E-57	Group B/C	KX154849.1	7.57E-56	Group B/C	HB3var21	5.98E-42	Group B/C
	26	MW014567	665	5	5	234.7	Group B/C	IT4var26	1.47E-62	Group B/C	KX154929.1	6.96E-51	Group B/C	PFI0005w	2.50E-50	Group B/C	MAL8P1.220	9.13E-40	Group B/C	PFCLINvar26	9.19E-35	Group B/C
80	1	MW014568	23971	5	4	310.3	DC8	KX154941.1	1.23E-107	DC8	KX154975.1	2.75E-79	DC8	KX154953.1	2.16E-70	DC8	RAJ116var32	6.08E-61	Group B/C	KX154939.1	6.12E-56	DC8
	2	MW014569	15915	5	3	105.5	Group B/C	IT4var32b	1.91E-46	DC8	IT4var19	1.91E-46	DC8	RAJ116var10	6.98E-36	Group B/C	IT4var41	6.98E-36	Group B/C	DD2var39	6.98E-36	Group B/C
	3	MW014570	13159	5	5	241.4	Group B/C	DD2var18	8.70E-55	Group B/C	PFCLINvar64	6.77E-51	Group B/C	IT4var66	6.77E-51	Group B/C	PFCLINvar66	1.13E-48	Group B/C	IT4var44	8.89E-40	Group B/C
	4	MW014571	12689	5	5	416.2	DC8	KX154878.1	2.04E-95	DC8	KX154914.1	2.04E-95	DC8	KX154961.1	2.04E-95	DC8	KX154856.1	1.25E-77	DC8	KX154975.1	6.00E-56	DC8
	6	MW014572	7534	5	5	869.1	var1csa	DD2var25	2.50E-179	var1csa	AJ420411	2.50E-179	var1csa	KX154917.1	4.19E-177	var1csa	IGHvar11	2.54E-169	var1csa	AAM55194	1.18E-167	var1csa
	7	MW014573	6682	5	5	388.3	EPCR	KX154954.1	9.78E-94	EPCR	KX154967.1	4.55E-92	EPCR	IGHvar09	6.01E-76	EPCR	IGHvar32	1.30E-72	EPCR	KX154966.1	1.33E-57	EPCR
	8	MW014574	5263	5	5	229.8	Group B/C	RAJ116var29	4.86E-92	Group B/C	KX154821.1	2.44E-35	Group B/C	PFCLINvar65	2.44E-35	Group B/C	IT4var59	2.44E-35	Group B/C	HB3var27	2.44E-35	Group B/C
	9	MW014575	4914	5	5	239.2	Group B/C	KX154908.1	5.10E-52	Group B/C	KX154969.1	1.10E-48	Group B/C	DD2var18	1.10E-48	Group B/C	RAJ116var09	5.13E-47	Group B/C	DD2var24	1.85E-46	Group B/C
	10	MW014576	4227	5	5	330.5	Group B/C	RAJ116var14	1.73E-71	Group B/C	PF10_0406	1.35E-67	Group B/C	KX154862.1	4.85E-67	Group B/C	IGHvar38	2.26E-65	Group B/C	IGHvar25	1.36E-62	Group B/C
	11	MW014577	3371	5	4	484.3	EPCR	KX154954.1	1.93E-155	EPCR	KX154967.1	2.04E-115	EPCR	KX154869.1	9.57E-109	EPCR	KX154966.1	1.24E-107	EPCR	DD2var22	2.80E-74	Group A-Rosetting/Unknown
	12	MW014578	3285	5	3	262.3	EPCR	IT4var02	4.41E-127	Group A-Rosetting/Unknown	KX154871.1	9.76E-109	EPCR	HB3var04	2.19E-80	Group A-Rosetting/Unknown	IT4var18	3.66E-78	EPCR	KX154893.1	1.32E-77	EPCR
93	1	MW014579	18647	5	5	220.1	Group B/C	IT4var10	2.02E-51	Group B/C	AAC05220	2.02E-51	Group B/C	HB3var24	2.61E-50	Group B/C	IT4var51	3.43E-39	Group B/C	DD2var18	2.08E-31	Group B/C
	2	MW014580	15461	5	5	282.3	Group B/C	IGHvar18	2.65E-70	Group B/C	PFL1955w	3.45E-64	Group B/C	IT4var34	5.81E-57	Group B/C	IT4var33	5.81E-57	Group B/C	KX154884.1	1.66E-37	Group B/C
	3	MW014581	14726	5	3	227.4	DC8	KX154939.1	1.22E-97	DC8	KX154885.1	1.26E-72	DC8	IT4var11	1.26E-72	Group B/C	KX154947.1	2.77E-59	DC8	PFCLINvar74	1.00E-53	Group B/C
	4	MW014582	10245	5	5	274.5	Group B/C	IGHvar08	9.49E-60	Group B/C	PFL1960w	2.06E-56	Group B/C	DD2var34	2.66E-55	Group B/C	PFCLINvar54	3.44E-54	Group B/C	DD2var18	1.60E-52	Group B/C

	5	MW014583	10076	5	5	241.4	Group B/C	IT4var05	4.09E-58	Group B/C	DD2var24	1.47E-57	Group B/C	IGHvar40	1.93E-46	Group B/C	IGHvar17	1.50E-42	Group B/C	IT4var51	2.52E-40	Group B/C
	6	MW014584	9658	5	5	226.4	Group B/C	PFCLINvar15	3.94E-78	Group B/C	DD2var26	4.08E-53	Group B/C	DD2var19	8.97E-40	Group B/C	HB3var28	9.10E-30	Group B/C	MAL6P1.4	3.27E-29	Group B/C
	7	MW014585	7197	5	3	158.2	DC8	KX154825.1	6.88E-71	DC8	KX154946.1	1.95E-56	DC8	IT4var39	1.55E-37	Group B/C	DD2var49	2.60E-35	EPCR	KX154870.1	4.35E-33	DC8
	8	MW014586	5034	5	4	329.2	EPCR	PFCLINvar49	2.69E-114	EPCR	IGHvar32	1.02E-78	EPCR	IGHvar09	4.74E-77	EPCR	KX154845.1	4.84E-62	EPCR	AAM55194	4.87E-57	var1csa
	10	MW014587	3766	5	5	261.8	Group B/C	RAJ116var34	5.67E-57	Group B/C	RAJ116var14	1.23E-53	Group B/C	IT4var62	4.41E-53	Group B/C	IGHvar38	1.59E-52	Group B/C	RAJ116var30	3.43E-49	Group B/C
	11	MW014588	3759	5	5	553.4	EPCR	KX154890.1	4.44E-122	EPCR	KX154923.1	9.62E-119	EPCR	KX154937.1	9.62E-119	EPCR	KX154857.1	4.54E-107	EPCR	KX154909.1	2.16E-90	EPCR
	12	MW014589	3409	5	2	1070.7	DC8	PFCLINvar31	0	DC8	RAJ116var11	1.85E-71	DC8	PF08_0140	5.17E-67	EPCR	KX154906.1	1.50E-37	Group B/C	AAB60251	1.50E-37	Group B/C
95	1	MW014590	13022	5	3	194.0	EPCR	HB3var02	1.27E-77	EPCR	DD2var04	3.57E-68	Group A-Rosetting/Unknown	KX154818.1	1.28E-67	EPCR	KX154888.1	6.10E-51	EPCR	PF08_0141	4.75E-47	Group A-Rosetting/Unknown
	2	MW014591	8402	5	4	156.2	Group B/C	PFD0615c	6.94E-46	Group B/C	PFD0625c	1.94E-41	Group B/C	KX154960.1	2.52E-40	EPCR	KX154920.1	1.96E-36	Group B/C	HB3var30	2.53E-35	Group B/C
	3	MW014592	7148	5	3	143.3	Group B/C	KX154825.1	1.11E-68	DC8	KX154946.1	4.00E-68	DC8	DD2var18	5.25E-57	Group B/C	PFCLINvar66	6.88E-46	Group B/C	PFL0935c	1.49E-42	Group B/C
	4	MW014593	6324	5	5	327.9	Group B/C	IT4var33	9.31E-85	Group B/C	PFL1955w	2.06E-66	Group B/C	KX154819.1	5.77E-62	Group B/C	IGHvar31	9.65E-60	Group B/C	KX154910.1	1.25E-58	Group B/C
	5	MW014594	5981	5	3	157.1	Group B/C	HB3var21	1.97E-76	Group B/C	RAJ116var22	4.44E-43	Group B/C	RAJ116var13	9.61E-40	Group B/C	IT4var20	2.08E-36	DC8	DD2var47	2.08E-36	DC8
	6	MW014595	5541	5	3	205.6	Group A-Rosetting/Unknown	KX154915.1	2.21E-80	Group A-Rosetting/Unknown	IGHvar22	2.23E-75	Group A-Rosetting/Unknown	DD2var40	2.96E-54	EPCR	DD2var09a	2.96E-54	EPCR	KX154842.1	4.95E-52	Group A-Rosetting/Unknown
	7	MW014596	5283	5	5	535.1	EPCR	KX154949.1	1.25E-112	EPCR	KX154875.1	4.51E-112	EPCR	KX154879.1	4.51E-112	EPCR	AAQ73928	2.71E-109	EPCR	KX154818.1	1.29E-92	EPCR
	8	MW014597	5246	5	5	440.5	EPCR	DD2var40	1.29E-97	EPCR	DD2var09a	1.29E-97	EPCR	PFCLINvar68	6.01E-96	EPCR	KX154886.1	1.03E-78	EPCR	KX154909.1	2.88E-74	EPCR
	9	MW014598	4584	5	5	145.7	Group B/C	IT4var32a	9.11E-35	Group B/C	IT4var14	9.11E-35	Group B/C	RAJ116var29	1.54E-27	Group B/C	HB3var48	5.52E-27	Group B/C	RAJ116var31	2.57E-25	Group B/C
	10	MW014599	3964	5	5	249.1	Group B/C	KX154905.1	4.05E-78	Group B/C	DD2var26	2.47E-65	Group B/C	DD2var30	4.28E-38	Group B/C	KX154860.1	1.99E-36	Group B/C	IT4var36	9.27E-35	Group B/C
	11	MW014600	3709	5	5	177.9	Group B/C	RAJ116var21	4.40E-43	Group B/C	RAJ116var26	1.58E-42	Group B/C	PFL0020w	7.42E-36	Group B/C	PF13_0364	5.77E-32	Group B/C	AAC47438	4.50E-28	Group B/C
	12	MW014601	3662	5	5	358.8	EPCR	PF11_0521	1.01E-98	EPCR	IT4var07	2.84E-89	EPCR	KX154875.1	3.84E-58	EPCR	KX154879.1	3.84E-58	EPCR	KX154949.1	3.84E-58	EPCR
	13	MW014602	2778	5	5	1349.6	Group B/C	RAJ116var26	0	Group B/C	PFCLINvar54	1.80E-101	Group B/C	DD2var11	1.80E-101	Group B/C	DD2var20	3.10E-79	Group B/C	IGHvar04	2.43E-70	Group B/C
	14	MW014603	2729	5	5	228.4	Group B/C	AY028643	2.07E-51	Group B/C	IT4var39	3.46E-49	Group B/C	PFI1830c	1.25E-48	Group B/C	IGHvar35	9.70E-45	Group B/C	KX154912.1	4.54E-38	Group B/C
	15	MW014604	2626	5	5	195.1	Group B/C	KX154868.1	3.32E-49	Group B/C	IGHvar33	3.35E-44	Group B/C	RAJ116var21	5.60E-42	Group B/C	HB3var29	7.29E-36	Group B/C	RAJ116var26	1.59E-27	Group B/C
98	1	MW014605	20667	5	4	176.6	Group B/C	AAB60251	1.29E-48	Group B/C	KX154934.1	1.00E-44	Group B/C	DD2var07	1.00E-44	Group B/C	PFCLINvar31	3.60E-44	DC8	PFCLINvar54	2.17E-41	Group B/C
	2	MW014606	15310	5	5	210.8	Group B/C	PFCLINvar27	1.48E-47	Group B/C	KX154840.1	4.14E-43	Group B/C	KX154976.1	4.14E-43	Group B/C	IT4var31	4.14E-43	Group B/C	KX154956.1	1.50E-37	Group B/C
	3	MW014607	14540	5	4	229.2	Group B/C	KX154841.1	2.11E-65	Group B/C	HB3var34	5.90E-61	Group B/C	KX154863.1	1.28E-57	Group B/C	PFCLINvar71	3.60E-48	Group B/C	MAL6P1.316	3.63E-43	DC8
	4	MW014608	13949	5	4	171.7	Group B/C	IT4var30	8.86E-55	Group B/C	KX154831.1	1.50E-42	Group B/C	KX154870.1	1.50E-42	DC8	KX154821.1	2.52E-40	Group B/C	MAL6P1.1	5.45E-37	Group B/C
	5	MW014609	4918	5	2	203.8	EPCR	PFCLINvar73	5.15E-171	EPCR	KX154943.1	1.00E-63	Group A-Rosetting/Unknown	KX154978.1	1.01E-58	Group A-Rosetting/Unknown	PFCLINvar75	3.67E-48	Group A-Rosetting/Unknown	KX154818.1	2.90E-34	EPCR
	6	MW014610	4600	5	4	200.0	Group B/C	IT4var61	9.03E-60	Group B/C	TM284S2var1	2.55E-50	Group B/C	PFC0005w	4.26E-48	Group B/C	KX154912.1	9.22E-45	Group B/C	IT4var20	7.18E-41	DC8
	7	MW014611	4568	5	5	236.9	Group B/C	IGHvar36	2.49E-50	Group B/C	KX154910.1	4.16E-48	Group B/C	PFE0005w	4.16E-48	Group B/C	PF13_0001	4.16E-48	Group B/C	PFL1955w	6.97E-46	Group B/C
	8	MW014612	3434	5	5	279.3	Group B/C	IT4var15	2.24E-70	Group B/C	DD2var13	2.24E-70	Group B/C	HB3var26	6.27E-66	Group B/C	KX154828.1	8.41E-40	Group B/C	KX154855.1	1.82E-36	Group B/C
	10	MW014613	2039	5	4	125.6	Group B/C	IT4var33	3.32E-44	Group B/C	IT4var32a	2.62E-30	Group B/C	IT4var14	2.62E-30	Group B/C	PF11_0008	3.41E-24	Group A-Rosetting/Unknown	HB3var12	1.23E-23	Group B/C
	11	MW014614	1658	5	5	709.0	EPCR	KX154875.1	1.98E-150	EPCR	KX154879.1	1.98E-150	EPCR	KX154949.1	1.98E-150	EPCR	AAQ73928	4.38E-132	EPCR	KX154904.1	2.64E-129	EPCR

	12	MW014615	1364	5	5	293.0	Group B/C	IT4var46	1.43E-82	Group B/C	TM284S2var1	1.47E-62	Group B/C	KX154916.1	6.86E-61	Group B/C	KX154823.1	4.16E-53	Group B/C	DD2var45	1.53E-37	Group B/C
	13	MW014616	1157	5	5	203.5	Group B/C	IT4var51	5.61E-47	Group B/C	RAJ116var04	7.30E-41	Group B/C	PFCLINvar54	2.63E-40	Group B/C	DD2var11	2.63E-40	Group B/C	KX154906.1	1.22E-38	Group B/C
	14	MW014617	1126	5	5	194.5	Group B/C	IGHvar04	7.37E-46	Group B/C	DD2var24	1.60E-42	Group B/C	IGHvar36	9.61E-40	Group B/C	PFCLINvar39	7.48E-36	Group B/C	DD2var12	3.48E-34	Group B/C
101	1	MW014618	36261	5	4	204.1	EPCR	KX154895.1	2.17E-85	EPCR	IT4var07	4.98E-42	EPCR	KX154844.1	2.32E-40	Group A-Rosetting/Unknown	KX154830.1	8.33E-40	EPCR	KX154867.1	8.33E-40	EPCR
	2	MW014619	24235	5	5	274.8	Group B/C	IT4var61	1.77E-86	Group B/C	KX154821.1	5.09E-62	Group B/C	PFC1120c	8.69E-45	Group B/C	PFC0005w	8.69E-45	Group B/C	TM284S2var1	2.43E-40	Group B/C
	3	MW014620	16899	5	3	207.4	DC8	IT4var13	7.49E-80	Group B/C	DD2var21	7.49E-80	Group B/C	KX154878.1	7.60E-70	DC8	KX154914.1	7.60E-70	DC8	KX154939.1	7.60E-70	DC8
	4	MW014621	11949	5	5	205.3	Group B/C	PFL0005w	1.41E-57	Group B/C	RAJ116var06	3.05E-54	Group B/C	AY028643	1.87E-36	Group B/C	DD2var33	5.24E-32	Group B/C	IGHvar25	1.13E-28	Group B/C
	5	MW014622	9777	5	5	235.3	Group B/C	IGHvar25	2.40E-55	Group B/C	PFCLINvar40	8.68E-50	Group B/C	IT4var32a	8.75E-45	Group B/C	IT4var14	8.75E-45	Group B/C	IGHvar21	3.15E-44	Group B/C
	6	MW014623	7444	5	4	466.0	EPCR	KX154869.1	8.99E-139	EPCR	KX154966.1	1.18E-127	EPCR	KX154954.1	2.00E-115	EPCR	KX154967.1	4.49E-87	EPCR	DD2var22	5.93E-71	Group A-Rosetting/Unknown
	7	MW014624	6799	5	5	254.1	Group B/C	PFE0005w	2.45E-60	Group B/C	PF13_0001	2.45E-60	Group B/C	AY028643	8.86E-55	Group B/C	KX154881.1	1.50E-42	Group B/C	KX154934.1	9.05E-40	Group B/C
109	1	MW014625	43465	5	4	309.2	EPCR	PFCLINvar49	1.28E-112	EPCR	KX154944.1	1.04E-78	Group A-Rosetting/Unknown	KX154845.1	1.05E-68	EPCR	IGHvar32	4.90E-67	EPCR	IGHvar09	1.06E-63	EPCR
	2	MW014626	14934	5	5	207.1	Group B/C	IGHvar37	8.08E-55	Group B/C	HB3var40	1.37E-42	Group B/C	DD2var44	2.29E-40	Group B/C	HB3var14	2.97E-39	Group B/C	MAL7P1.212	1.07E-33	Group B/C
	3	MW014627	8235	5	5	254.6	Group B/C	RAJ116var29	1.48E-72	Group B/C	KX154912.1	7.08E-51	Group B/C	KX154821.1	7.13E-46	Group B/C	DD2var50	7.13E-46	Group B/C	RAJ116var06	4.29E-43	Group B/C
	4	MW014628	7789	5	3	212.9	DC8	KX154953.1	2.65E-99	DC8	RAJ116var32	2.15E-65	Group B/C	KX154885.1	2.16E-60	DC8	KX154939.1	2.18E-55	DC8	KX154894.1	6.10E-51	Group B/C
	5	MW014629	6530	5	5	269.1	Group B/C	AY028643	2.51E-60	Group B/C	KX154821.1	7.03E-56	Group B/C	PFCLINvar40	3.27E-54	Group B/C	DD2var18	1.97E-51	Group B/C	DD2var31	7.08E-51	Group B/C
	6	MW014630	3220	5	5	159.5	Group B/C	KX154934.1	2.08E-36	Group B/C	IGHvar16	3.48E-34	Group B/C	HB3var33	3.48E-34	Group B/C	RAJ116var33	2.71E-30	Group B/C	IGHvar17	4.53E-28	Group B/C
	7	MW014631	2947	5	5	295.3	Group B/C	PFL1950w	3.12E-69	Group B/C	HB3var23	3.12E-69	Group B/C	IGHvar17	5.29E-57	Group B/C	DD2var31	4.12E-53	Group B/C	KX154907.1	2.48E-50	Group B/C
	8	MW014632	2902	5	5	848.7	var1csa	AAN86340	5.15E-171	var1csa	PFE1640w	5.15E-171	var1csa	KX154932.1	1.85E-170	var1csa	KX154948.1	1.85E-170	var1csa	KX154820.1	2.40E-169	var1csa
	9	MW014633	2609	5	5	277.6	Group B/C	IT4var46	1.70E-96	Group B/C	TM284S2var1	2.95E-69	Group B/C	DD2var33	1.84E-41	Group B/C	DD2var39	1.11E-38	Group B/C	RAJ116var39	2.40E-35	Group B/C
	10	MW014634	2499	5	5	192.4	Group B/C	RAJ116var39	6.94E-46	Group B/C	IT4var67	8.98E-45	Group B/C	DD2var39	3.25E-39	Group B/C	KX154905.1	3.28E-34	Group B/C	RAJ116var10	5.48E-32	Group B/C
	11	MW014635	2312	5	5	258.3	Group B/C	IGHvar29	2.75E-60	Group B/C	PFCLINvar41	2.14E-56	Group B/C	PFCLINvar43	7.69E-56	Group B/C	IT4var30	1.68E-47	Group B/C	PFCLINvar08	6.07E-42	Group B/C
	12	MW014636	1800	5	5	208.2	Group B/C	KX154906.1	7.99E-51	Group B/C	IGHvar35	1.73E-47	Group B/C	PFCLINvar43	6.27E-42	Group B/C	DD2var45	2.25E-41	Group B/C	PFCLINvar07	2.96E-30	Group B/C
	13	MW014637	1513	5	4	364.3	DC8	KX154939.1	9.45E-104	DC8	KX154947.1	4.40E-102	DC8	KX154873.1	9.65E-89	DC8	KX154953.1	1.28E-72	DC8	IT4var13	1.28E-67	Group B/C
	14	MW014638	1144	5	4	247.5	EPCR	KX154936.1	4.82E-72	EPCR	PFCLINvar62	3.75E-68	EPCR	RAJ116var03	2.94E-59	EPCR	KX154871.1	6.41E-51	EPCR	KX154877.1	6.41E-51	DC8
	15	MW014639	1117	5	5	205.8	Group B/C	RAJ116var29	1.43E-67	Group B/C	DD2var19	4.11E-43	Group B/C	KX154971.1	4.16E-33	Group B/C	RAJ116var26	4.16E-33	Group B/C	KX154824.1	1.50E-32	Group B/C
	16	MW014640	1031	5	5	262.7	Group B/C	HB3var10	1.64E-91	Group B/C	KX154855.1	1.35E-47	Group B/C	PF08_0142	1.35E-47	Group B/C	KX154916.1	1.76E-41	Group B/C	RAJ116var39	3.80E-38	Group B/C
	17	MW014641	1004	5	3	305.7	EPCR	KX154845.1	2.14E-110	EPCR	IGHvar09	2.15E-105	EPCR	IGHvar32	4.73E-92	EPCR	DD2var22	2.20E-90	Group A-Rosetting/Unknown	IT4var60	1.05E-73	Group A-Rosetting/Unknown
	18	MW014642	786	5	5	243.9	Group B/C	IGHvar08	2.69E-70	Group B/C	RAJ116var21	1.65E-52	Group B/C	PFCLINvar25	1.29E-48	Group B/C	KX154828.1	4.69E-38	Group B/C	IT4var44	4.69E-38	Group B/C
	19	MW014643	771	5	4	372.5	EPCR	KX154845.1	1.00E-103	EPCR	IGHvar09	1.01E-98	EPCR	IGHvar32	2.18E-95	EPCR	KX154944.1	1.04E-78	Group A-Rosetting/Unknown	PFCLINvar49	1.34E-77	EPCR
	20	MW014644	738	5	4	256.9	Group A-Rosetting/Unknown	IT4var60	1.35E-77	Group A-Rosetting/Unknown	IGHvar12	4.87E-77	Group A-Rosetting/Unknown	KX154887.1	6.44E-61	EPCR	HB3var05	8.39E-55	Group A-Rosetting/Unknown	KX154915.1	2.35E-50	Group A-Rosetting/Unknown
	21	MW014645	643	5	5	263.5	Group B/C	IT4var54	5.33E-82	Group B/C	IT4var45	7.10E-61	Group B/C	DD2var34	3.37E-44	Group B/C	PFD0625c	2.63E-40	Group B/C	DD2var24	9.45E-40	Group B/C

110	1	MW014646	29930	5	5	224.7	Group B/C	PF08_0142	1.41E-67	Group B/C	IGHvar38	5.23E-47	Group B/C	KX154955.1	5.26E-42	Group B/C	IGHvar31	1.91E-36	Group B/C	KX154905.1	2.47E-35	Group B/C
	2	MW014647	29093	5	5	305.5	Group B/C	RAJ116var14	4.65E-77	Group B/C	IGHvar38	1.30E-72	Group B/C	PF10_0406	6.14E-61	Group B/C	KX154815.1	2.24E-50	Group B/C	HB3var08	3.75E-48	Group B/C
	3	MW014648	11722	5	3	142.9	DC8	PFCLINvar09	2.79E-54	Group B/C	KX154933.1	1.00E-53	DC8	HB3var21	1.01E-48	Group B/C	KX154846.1	1.31E-47	DC8	KX154939.1	1.02E-43	DC8
	4	MW014649	9801	5	4	309.7	EPCR	KX154926.1	2.16E-90	EPCR	KX154899.1	2.83E-79	Group A-Rosetting/Unknown	DD2var42	1.02E-78	EPCR	KX154874.1	1.70E-76	EPCR	KX154959.1	4.80E-67	EPCR
	5	MW014650	9535	5	5	249.5	Group B/C	DD2var34	1.63E-57	Group B/C	IT4var62	4.56E-53	Group B/C	HB3var21	2.12E-51	Group B/C	PFD1015c	5.94E-47	Group B/C	HB3var19	3.57E-44	Group B/C
	6	MW014651	7700	5	4	185.6	Group B/C	RAJ116var08	1.47E-62	DC8	RAJ116var22	5.38E-52	Group B/C	KX154831.1	1.16E-48	Group B/C	IT4var61	1.16E-48	Group B/C	RAJ116var36	3.28E-39	Group B/C
	7	MW014652	3626	5	5	228.4	Group B/C	IT4var54	1.56E-62	Group B/C	KX154828.1	4.44E-48	Group B/C	PFI1830c	1.24E-43	Group B/C	IGHvar33	5.79E-42	Group B/C	RAJ116var28	7.54E-36	Group B/C
	8	MW014653	3626	5	5	240.2	Group B/C	IT4var30	1.91E-61	Group B/C	AY028643	3.24E-49	Group B/C	PFB1055c	1.95E-46	Group B/C	RAJ116var27	3.26E-44	Group B/C	PFA0005w	1.52E-42	Group B/C
	9	MW014654	3493	5	5	284.7	Group B/C	IT4var61	8.60E-70	Group B/C	IT4var16	4.03E-63	Group B/C	DD2var23	1.46E-57	Group B/C	KX154821.1	3.16E-54	Group B/C	TM284S2var1	1.15E-43	Group B/C
	10	MW014655	3337	5	5	277.7	Group B/C	PFCLINvar07	3.59E-69	Group B/C	RAJ116var09	2.17E-61	Group B/C	PFCLINvar46	7.87E-56	Group B/C	KX154908.1	3.69E-49	Group B/C	MAL7P1.56	7.99E-46	Group B/C
	11	MW014656	2473	5	5	172.0	Group B/C	KX154979.1	3.07E-49	Group B/C	PFD0625c	1.46E-32	Group B/C	HB3var20	1.46E-32	Group B/C	IT4var47	5.24E-32	Group B/C	IT4var44	3.15E-29	Group B/C
	12	MW014657	1538	5	5	281.5	Group B/C	PFCLINvar40	2.99E-74	Group B/C	KX154822.1	2.34E-65	Group B/C	RAJ116var10	3.10E-49	Group B/C	IT4var32a	4.00E-48	Group B/C	IT4var14	4.00E-48	Group B/C
	13	MW014658	1239	5	5	359.9	Group B/C	IT4var15	1.01E-83	Group B/C	DD2var13	1.01E-83	Group B/C	HB3var26	4.70E-82	Group B/C	PFD1005c	2.89E-64	Group B/C	DD2var29	8.21E-50	Group B/C
	14	MW014659	1223	5	5	214.8	Group B/C	KX154970.1	6.39E-66	Group B/C	KX154929.1	1.41E-47	Group B/C	KX154976.1	2.40E-35	Group B/C	IT4var31	2.40E-35	Group B/C	IT4var61	3.10E-34	Group B/C
	15	MW014660	1034	5	5	285.0	Group B/C	KX154832.1	2.69E-74	Group B/C	HB3var12	4.50E-72	Group B/C	HB3var40	2.15E-50	Group B/C	KX154866.1	6.03E-46	Group B/C	HB3var14	6.03E-46	Group B/C
	17	MW014661	904	5	4	255.8	EPCR	KX154874.1	1.70E-76	EPCR	DD2var42	2.22E-70	EPCR	KX154891.1	6.22E-66	Group A-Rosetting/Unknown	KX154830.1	6.30E-56	EPCR	KX154867.1	6.30E-56	EPCR
	18	MW014662	806	5	3	133.7	DC8	KX154933.1	2.84E-53	DC8	PFCLINvar09	1.03E-47	Group B/C	KX154846.1	4.78E-46	DC8	KX154939.1	1.35E-36	DC8	PFCLINvar36	2.94E-28	Group B/C
	19	MW014663	735	5	5	292.0	Group B/C	RAJ116var14	1.81E-71	Group B/C	IGHvar38	1.10E-63	Group B/C	PF08_0142	3.08E-59	Group B/C	DD2var30	1.43E-57	Group B/C	KX154955.1	1.13E-43	Group B/C
	20	MW014664	661	5	5	157.6	Group B/C	TM284S2var1	5.06E-36	Group B/C	RAJ116var24	2.35E-34	Group B/C	IGHvar25	8.46E-34	Group B/C	PFC0005w	8.52E-29	Group B/C	IT4var61	3.06E-28	Group B/C
	21	MW014665	648	5	5	188.7	Group B/C	HB3var07	1.10E-42	Group B/C	KX154955.1	2.37E-39	Group B/C	PFCLINvar40	3.07E-38	Group B/C	IGHvar31	1.43E-36	Group B/C	PFA0765c	1.85E-35	Group B/C
	23	MW014666	587	5	5	635.1	EPCR	KX154888.1	4.11E-177	EPCR	KX154903.1	4.35E-137	EPCR	KX154875.1	3.51E-108	EPCR	KX154879.1	3.51E-108	EPCR	KX154949.1	3.51E-108	EPCR
	24	MW014667	528	5	5	184.7	Group B/C	PF08_0142	2.37E-59	Group B/C	IT4var36	6.83E-35	Group B/C	KX154849.1	1.14E-32	Group B/C	KX154823.1	1.48E-31	Group B/C	KX154828.1	6.88E-30	Group B/C
	25	MW014668	465	5	5	242.1	Group B/C	IGHvar38	6.18E-56	Group B/C	RAJ116var14	1.03E-53	Group B/C	KX154849.1	6.23E-51	Group B/C	KX154815.1	1.05E-43	Group B/C	PFCLINvar24	1.76E-41	Group B/C
112	1	MW014669	28401	5	2	233.3	Group A-Rosetting/Unknown	KX154943.1	3.19E-148	Group A-Rosetting/Unknown	KX154978.1	1.62E-86	Group A-Rosetting/Unknown	PFCLINvar73	3.57E-68	EPCR	KX154875.1	1.71E-46	EPCR	KX154879.1	1.71E-46	EPCR
	2	MW014670	20064	5	4	332.5	EPCR	IGHvar26	6.00E-91	EPCR	KX154911.1	1.00E-88	EPCR	IT4var18	1.69E-81	EPCR	PFD0020c	2.20E-75	DC8	KX154893.1	2.85E-74	EPCR
	3	MW014671	15048	5	3	231.2	EPCR	IT4var08	7.97E-85	Group A-Rosetting/Unknown	KX154945.1	1.33E-82	EPCR	IT4var07	1.74E-76	EPCR	PF11_0521	2.91E-74	EPCR	DD2var22	1.79E-56	Group A-Rosetting/Unknown
	4	MW014672	14109	5	4	238.6	EPCR	KX154895.1	1.01E-83	EPCR	HB3var1csa	2.93E-54	var1csa	KX154945.1	3.79E-53	EPCR	IT4var07	3.79E-53	EPCR	KX154887.1	1.77E-51	EPCR
	5	MW014673	11244	5	3	255.6	Group A-Rosetting/Unknown	IGHvar12	7.82E-105	Group A-Rosetting/Unknown	KX154891.1	7.98E-90	Group A-Rosetting/Unknown	KX154926.1	4.84E-82	EPCR	PFCLINvar62	4.94E-67	EPCR	RAJ116var19	3.85E-63	Group A-Rosetting/Unknown
	6	MW014674	7434	5	2	122.6	EPCR	KX154890.1	4.75E-62	EPCR	KX154959.1	4.75E-62	EPCR	KX154842.1	2.86E-59	Group A-Rosetting/Unknown	HB3var1csa	8.00E-55	var1csa	IGHvar24	1.73E-51	Group A-Rosetting/Unknown
	7	MW014675	5570	5	4	201.4	Group B/C	KX154846.1	9.83E-69	DC8	IGHvar05	2.13E-65	Group B/C	PFD1015c	1.01E-48	Group B/C	PFCLINvar71	1.69E-46	Group B/C	PFCLINvar36	1.02E-43	Group B/C
	8	MW014676	4187	5	5	186.4	Group B/C	KX154910.1	2.43E-45	Group B/C	KX154853.1	6.81E-41	Group B/C	IT4var23	1.14E-38	Group B/C	KX154955.1	1.48E-32	Group B/C	PFD0630c	1.48E-32	Group B/C
	9	MW014677	3738	5	3	285.7	EPCR	HB3var02	2.67E-99	EPCR	DD2var32	9.61E-99	EPCR	IGHvar30	7.54E-90	EPCR	KX154820.1	5.95E-76	var1csa	KX154858.1	2.77E-74	var1csa

	10	MW014678	3590	5	5	327.9	Group B/C	IT4var33	9.31E-85	Group B/C	PFL1955w	2.06E-66	Group B/C	KX154819.1	5.77E-62	Group B/C	IGHvar31	9.65E-60	Group B/C	KX154910.1	1.25E-58	Group B/C
	11	MW014679	1910	5	4	229.1	Group B/C	KX154894.1	3.86E-68	Group B/C	PFL0005w	3.91E-58	Group B/C	RAJ116var06	5.06E-57	Group B/C	RAJ116var08	1.82E-56	DC8	RAJ116var22	1.10E-48	Group B/C
	12	MW014680	1312	5	3	326.8	EPCR	IGHvar14	2.50E-179	EPCR	IGHvar26	1.02E-88	EPCR	PFD0020c	6.25E-76	DC8	KX154936.1	6.38E-61	EPCR	KX154938.1	1.38E-57	DC8
	13	MW014681	1163	5	5	232.6	Group B/C	IT4var36	3.72E-73	Group B/C	PFL0020w	3.83E-53	Group B/C	HB3var10	4.99E-47	Group B/C	PFC1120c	3.94E-33	Group B/C	KX154831.1	8.53E-30	Group B/C
	14	MW014682	977	5	3	182.2	Group B/C	PFCLINvar06	2.31E-95	Group B/C	DD2var07	8.90E-45	Group B/C	DD2var41	3.20E-44	Group B/C	IT4var03	8.97E-40	Type 3 var	KX154825.1	1.52E-27	DC8
	16	MW014683	694	5	5	267.4	DC8	IT4var20	9.18E-60	DC8	DD2var47	9.18E-60	DC8	KX154942.1	5.57E-52	DC8	MAL6P1.316	5.57E-52	DC8	KX154889.1	1.56E-47	DC8
	17	MW014684	687	5	4	214.6	Group B/C	PFCLINvar52	1.77E-76	Group B/C	KX154907.1	5.10E-52	Group B/C	PFL0020w	3.07E-49	Group B/C	KX154928.1	6.64E-46	DC8	KX154831.1	8.65E-40	Group B/C
	18	MW014685	667	5	5	469.4	EPCR	KX154911.1	6.08E-101	EPCR	KX154887.1	4.74E-97	EPCR	IT4var18	4.74E-97	EPCR	KX154893.1	2.22E-90	EPCR	IGHvar26	1.34E-87	EPCR
114	1	MW014686	47371	5	5	128.2	Group B/C	KX154940.1	1.06E-33	Group B/C	KX154905.1	4.95E-32	Group B/C	DD2var44	2.98E-29	Group B/C	PFB0010w	8.40E-20	Group B/C	KX154950.1	5.06E-17	Group B/C
	2	MW014687	32826	5	3	288.0	EPCR	PFCLINvar73	2.57E-119	EPCR	DD2var32	7.46E-90	EPCR	HB3var02	5.85E-81	EPCR	KX154943.1	4.56E-77	Group A-Rosetting/Unknown	KX154978.1	4.65E-62	Group A-Rosetting/Unknown
	3	MW014688	12628	5	5	185.0	Group B/C	KX154896.1	7.67E-41	Group B/C	RAJ116var30	9.93E-40	Group B/C	IT4var62	4.62E-38	Group B/C	IT4var68	1.66E-37	Group B/C	RAJ116var31	1.67E-32	Group B/C
	4	MW014689	9950	5	3	247.3	Group A-Rosetting/Unknown	KX154844.1	2.22E-90	Group A-Rosetting/Unknown	KX154891.1	4.80E-87	Group A-Rosetting/Unknown	KX154874.1	2.24E-85	EPCR	KX154845.1	4.91E-72	EPCR	IGHvar12	4.91E-72	Group A-Rosetting/Unknown
	5	MW014690	9272	5	4	289.2	DC8	KX154953.1	4.54E-87	DC8	KX154939.1	2.14E-75	DC8	KX154941.1	2.79E-69	DC8	RAJ116var32	1.30E-67	Group B/C	KX154925.1	2.19E-60	DC8
	6	MW014691	7853	5	3	272.3	EPCR	KX154926.1	1.28E-117	EPCR	IGHvar39	7.98E-90	EPCR	ACJ68328	2.28E-70	Group A-Rosetting/Unknown	RAJ116var07	2.95E-69	Group A-Rosetting/Unknown	KX154890.1	4.94E-67	EPCR
	7	MW014692	6999	5	5	1306.3	EPCR	KX154901.1	0	EPCR	KX154872.1	3.89E-88	EPCR	KX154935.1	2.37E-75	EPCR	IGHvar27	2.37E-75	EPCR	KX154918.1	2.39E-70	EPCR
	9	MW014693	6018	5	3	269.0	EPCR	PF11_0521	7.65E-120	EPCR	IT4var22	2.85E-94	EPCR	DQ408104	2.85E-94	Type 3 var	IT4var08	3.00E-59	Group A-Rosetting/Unknown	KX154945.1	5.01E-57	EPCR
	10	MW014694	5738	5	5	187.9	Group B/C	IT4var51	2.63E-50	Group B/C	PFD0630c	7.43E-41	Group B/C	PFD0635c	4.47E-38	Group B/C	IT4var34	5.82E-32	Group B/C	HB3var24	2.71E-30	Group B/C
	11	MW014695	5416	5	5	219.7	Group B/C	KX154831.1	8.84E-50	Group B/C	PFL0020w	3.18E-49	Group B/C	HB3var21	1.49E-42	Group B/C	IT4var32a	6.93E-41	Group B/C	IT4var14	6.93E-41	Group B/C
	12	MW014696	3534	5	4	110.1	Group B/C	RAJ116var08	6.35E-71	DC8	RAJ116var22	1.09E-48	Group B/C	KX154866.1	8.75E-25	Group B/C	KX154883.1	8.81E-20	Group B/C	IGHvar04	8.81E-20	Group B/C
	13	MW014697	3333	5	5	489.4	EPCR	IGHvar14	1.67E-111	EPCR	KX154911.1	2.83E-99	EPCR	IGHvar26	2.83E-99	EPCR	KX154902.1	1.03E-93	EPCR	KX154887.1	2.87E-89	EPCR
	14	MW014698	2725	5	3	170.0	EPCR	PFCLINvar75	1.03E-73	Group A-Rosetting/Unknown	KX154960.1	8.04E-65	EPCR	KX154918.1	2.25E-60	EPCR	PFCLINvar05	8.21E-50	Group A-Rosetting/Unknown	KX154958.1	4.94E-47	EPCR
115	1	MW014699	21516	5	4	273.0	EPCR	KX154936.1	2.19E-85	EPCR	PFCLINvar62	2.26E-65	EPCR	KX154945.1	1.36E-62	EPCR	IT4var07	1.36E-62	EPCR	IT4var08	6.32E-61	Group A-Rosetting/Unknown
	2	MW014700	10656	5	1	1000.0	EPCR	IGHvar23	0	EPCR	PFCLINvar69	1.42E-52	Group A-Rosetting/Unknown	IT4var09	1.43E-47	Group A-Rosetting/Unknown	KX154978.1	5.13E-47	Group A-Rosetting/Unknown	KX154915.1	1.85E-46	Group A-Rosetting/Unknown
	3	MW014701	7432	5	5	251.2	Group B/C	PFCLINvar55	9.36E-70	Group B/C	DD2var35	4.45E-53	Group B/C	KX154828.1	5.75E-52	Group B/C	IT4var39	5.84E-42	Group B/C	AAB60251	4.54E-38	Group B/C
	4	MW014702	6620	5	4	305.3	EPCR	PFCLINvar68	6.07E-96	EPCR	IGHvar14	7.97E-85	EPCR	ACJ68328	4.83E-77	Group A-Rosetting/Unknown	KX154936.1	1.36E-67	EPCR	KX154909.1	8.25E-60	EPCR
	5	MW014703	6310	5	5	315.7	Group B/C	PFCLINvar64	1.53E-72	Group B/C	IT4var66	1.53E-72	Group B/C	IT4var45	2.01E-61	Group B/C	RAJ116var36	9.34E-60	Group B/C	IGHvar33	4.38E-53	Group B/C
	6	MW014704	6097	5	3	256.2	Group A-Rosetting/Unknown	ACJ68328	2.83E-99	Group A-Rosetting/Unknown	IGHvar12	1.03E-88	Group A-Rosetting/Unknown	KX154830.1	4.87E-77	EPCR	KX154867.1	4.87E-77	EPCR	KX154915.1	2.28E-70	Group A-Rosetting/Unknown
	7	MW014705	6034	5	5	237.3	Group B/C	IT4var45	4.38E-53	Group B/C	IGHvar04	4.38E-53	Group B/C	MAL7P1.50	1.58E-47	Group B/C	IGHvar06	7.37E-46	Group B/C	KX154840.1	2.06E-41	Group B/C
	8	MW014706	4908	5	2	44.8	Group A-Rosetting/Unknown	DD2var04	1.35E-27	Group A-Rosetting/Unknown	PFCLINvar71	1.36E-22	Group B/C	KX154856.1	2.27E-20	DC8	PF08_0141	1.06E-18	Group A-Rosetting/Unknown	DD2var31	3.80E-18	Group B/C
	9	MW014707	3785	5	5	212.0	Group B/C	PF07_0048	8.51E-45	Group B/C	RAJ116var13	3.96E-43	Group B/C	IT4var26	3.96E-43	Group B/C	PF08_0106	1.42E-42	Group B/C	PFCLINvar23	5.12E-42	Group B/C
	10	MW014708	3740	5	4	150.6	EPCR	PFCLINvar33	8.36E-90	Group A-Rosetting/Unknown	KX154901.1	3.11E-64	EPCR	KX154918.1	9.10E-30	EPCR	KX154949.1	9.10E-30	EPCR	AAQ73928	9.10E-30	EPCR

	11	MW014709	3258	5	3	264.5	DC8	KX154877.1	6.01E-116	DC8	KX154847.1	2.29E-75	DC8	KX154898.1	2.29E-75	DC8	PF08_0140	8.65E-40	EPCR	RAJ116var06	1.45E-37	Group B/C
	12	MW014710	3158	5	4	107.3	Group B/C	PFCLINvar31	1.16E-33	DC8	KX154864.1	6.97E-31	Group B/C	MAL7P1.50	1.17E-28	Group B/C	KX154900.1	5.43E-27	Group B/C	DD2var29	1.17E-23	Group B/C
	13	MW014711	2812	5	5	869.1	var1csa	KX154917.1	1.94E-180	var1csa	DD2var25	2.50E-179	var1csa	AJ420411	2.50E-179	var1csa	IGHvar11	2.54E-169	var1csa	AAM55194	2.56E-164	var1csa
	14	MW014712	2775	5	5	369.0	Group B/C	KX154955.1	2.60E-100	Group B/C	HB3var24	4.47E-78	Group B/C	KX154956.1	1.63E-67	Group B/C	IGHvar29	2.73E-65	Group B/C	RAJ116var09	2.12E-61	Group B/C
	15	MW014713	2402	5	5	142.1	Group B/C	KX154863.1	2.22E-35	Group B/C	IGHvar06	2.22E-35	Group B/C	TM284S2var1	1.73E-31	Group B/C	PF08_0103	1.35E-27	Group B/C	PFC1120c	6.35E-16	Group B/C
	16	MW014714	2175	5	5	546.7	EPCR	AAQ73928	2.68E-119	EPCR	KX154903.1	1.61E-116	EPCR	KX154888.1	3.49E-113	EPCR	KX154949.1	5.91E-101	EPCR	KX154875.1	2.13E-100	EPCR
	17	MW014715	2136	5	5	228.0	Group B/C	HB3var40	4.06E-63	Group B/C	DD2var18	8.92E-50	Group B/C	HB3var26	4.18E-43	Group B/C	DD2var51	3.25E-39	Group B/C	HB3var33	1.96E-36	Group B/C
	18	MW014716	1617	5	5	199.3	Group B/C	PFCLINvar41	6.36E-42	Group B/C	RAJ116var30	2.29E-41	Group B/C	HB3var13	2.29E-41	Group B/C	RAJ116var34	1.06E-39	Group B/C	PFE0005w	1.38E-38	Group B/C
	19	MW014717	1271	5	4	256.1	DC8	KX154942.1	5.54E-67	DC8	MAL6P1.316	5.54E-67	DC8	IT4var20	1.55E-62	DC8	DD2var47	1.55E-62	DC8	IGHvar36	9.47E-50	Group B/C
	20	MW014718	1130	5	5	195.2	Group B/C	IGHvar35	7.08E-51	Group B/C	PFL2665c	1.19E-43	Group B/C	KX154850.1	2.01E-36	Group B/C	PFCLINvar22	9.35E-35	Group B/C	IGHvar29	4.35E-33	Group B/C
	21	MW014719	1120	5	3	314.6	EPCR	KX154902.1	9.49E-129	EPCR	PFD0020c	5.75E-121	DC8	KX154938.1	5.87E-106	DC8	KX154911.1	2.14E-95	EPCR	KX154893.1	1.29E-92	EPCR
	22	MW014720	1118	5	4	179.4	Group B/C	PFC1120c	1.59E-62	Group B/C	KX154928.1	2.66E-60	DC8	IT4var61	2.72E-45	Group B/C	HB3var16	2.12E-41	Group B/C	RAJ116var22	4.61E-33	Group B/C
	23	MW014721	958	5	5	224.8	Group B/C	KX154916.1	8.55E-55	Group B/C	PFCLINvar45	2.39E-50	Group B/C	PF08_0142	6.70E-46	Group B/C	PFCLINvar52	1.45E-42	Group B/C	IT4var36	8.79E-35	Group B/C
	24	MW014722	854	5	5	496.1	EPCR	AAQ73928	1.27E-102	EPCR	KX154903.1	2.13E-100	EPCR	KX154949.1	2.75E-99	EPCR	KX154875.1	9.90E-99	EPCR	KX154879.1	9.90E-99	EPCR
	25	MW014723	793	5	5	256.9	Group B/C	KX154819.1	2.42E-85	Group B/C	IT4var27	9.22E-45	Group B/C	DD2var01b	9.22E-45	Group B/C	DD2var01a	9.22E-45	Group B/C	PFCLINvar08	7.18E-41	Group B/C
	26	MW014724	774	5	4	281.4	EPCR	PFCLINvar49	1.68E-91	EPCR	KX154911.1	6.27E-66	EPCR	IGHvar09	6.27E-66	EPCR	PFI1820w	4.89E-62	Type 3 var	KX154845.1	6.32E-61	EPCR
	27	MW014725	688	5	5	216.0	Group B/C	DD2var35	1.72E-52	Group B/C	HB3var29	1.73E-47	Group B/C	HB3var30	6.22E-47	Group B/C	PFCLINvar54	2.27E-36	Group B/C	DD2var11	2.27E-36	Group B/C
	28	MW014726	601	5	5	566.7	var1csa	KX154917.1	9.81E-119	var1csa	DD2var25	9.81E-119	var1csa	AJ420411	9.81E-119	var1csa	IGHvar11	9.95E-109	var1csa	AAM55194	2.15E-105	var1csa
	29	MW014727	564	5	3	111.4	Group B/C	IT4var64	3.60E-44	Group A-Rosetting/Unknown	DD2var52	3.60E-44	Group A-Rosetting/Unknown	KX154955.1	4.66E-43	Group B/C	KX154896.1	6.07E-37	Group B/C	PFE0005w	1.31E-33	Group B/C
	30	MW014728	534	5	5	212.4	Group B/C	IT4var62	5.52E-52	Group B/C	RAJ116var31	2.59E-45	Group B/C	IGHvar13	2.01E-41	Group B/C	PFL1950w	1.21E-38	Group B/C	HB3var23	1.21E-38	Group B/C
	31	MW014729	522	5	5	268.0	Group B/C	KX154979.1	8.81E-65	Group B/C	IGHvar25	4.13E-58	Group B/C	IT4var61	3.21E-54	Group B/C	PFCLINvar41	1.51E-47	Group B/C	TM284S2var1	5.42E-47	Group B/C
	32	MW014730	522	5	5	255.1	Group B/C	KX154922.1	1.20E-58	Group B/C	PFCLINvar64	5.57E-57	Group B/C	IT4var66	5.57E-57	Group B/C	HB3var13	3.40E-44	Group B/C	PFCLINvar41	5.69E-42	Group B/C
	33	MW014731	501	5	5	174.7	Group B/C	DD2var35	1.46E-41	Group B/C	AAB60251	1.14E-37	Group B/C	PFD0005w	1.14E-37	Group B/C	KX154828.1	2.46E-34	Group B/C	PFCLINvar54	4.14E-27	Group B/C
	34	MW014732	488	5	4	268.5	Group B/C	IGHvar20	2.12E-150	Group B/C	IGHvar01	5.20E-57	Group B/C	PFCLINvar30	4.05E-53	DC8	HB3var07	1.50E-32	Group B/C	RAJ116var39	1.94E-31	Group B/C
	35	MW014733	445	5	3	136.1	EPCR	KX154944.1	1.08E-58	Group A-Rosetting/Unknown	KX154959.1	3.02E-54	EPCR	PF13_0003	5.05E-52	Group A-Rosetting/Unknown	DD2var40	5.12E-42	EPCR	DD2var09a	5.12E-42	EPCR
	36	MW014734	435	5	5	601.2	var1csa	IT4var35	2.68E-124	var1csa	AAM55194	1.61E-121	var1csa	TM284var3	1.61E-121	var1csa	IGHvar11	1.61E-121	var1csa	KX154917.1	5.85E-116	var1csa
	37	MW014735	434	5	4	159.4	Group B/C	KX154906.1	1.64E-42	Group B/C	KX154971.1	2.74E-40	Group B/C	IGHvar40	2.74E-40	Group B/C	PFD1235w	3.54E-39	EPCR	MAL7P1.1	3.54E-39	Group B/C
	38	MW014736	393	5	4	221.5	Group B/C	IT4var23	1.04E-64	Group B/C	IT4var45	4.85E-58	Group B/C	IGHvar33	4.88E-53	Group B/C	PFCLINvar08	1.37E-48	Group B/C	KX154825.1	2.96E-45	DC8
116	1	MW014737	22773	5	4	179.4	Group B/C	PFC1120c	1.59E-62	Group B/C	KX154928.1	2.66E-60	DC8	IT4var61	2.72E-45	Group B/C	HB3var16	2.12E-41	Group B/C	RAJ116var22	4.61E-33	Group B/C
	2	MW014738	21411	5	5	315.7	Group B/C	PFCLINvar64	1.53E-72	Group B/C	IT4var66	1.53E-72	Group B/C	IT4var45	2.01E-61	Group B/C	RAJ116var36	9.34E-60	Group B/C	IGHvar33	4.38E-53	Group B/C
	3	MW014739	11039	5	4	150.6	EPCR	PFCLINvar33	8.36E-90	Group A-Rosetting/Unknown	KX154901.1	3.11E-64	EPCR	KX154918.1	9.10E-30	EPCR	KX154949.1	9.10E-30	EPCR	AAQ73928	9.10E-30	EPCR
	4	MW014740	9850	5	5	224.8	Group B/C	KX154916.1	8.55E-55	Group B/C	PFCLINvar45	2.39E-50	Group B/C	PF08_0142	6.70E-46	Group B/C	PFCLINvar52	1.45E-42	Group B/C	IT4var36	8.79E-35	Group B/C

	5	MW014741	9621	5	5	251.2	Group B/C	PFCLINvar55	9.36E-70	Group B/C	DD2var35	4.45E-53	Group B/C	KX154828.1	5.75E-52	Group B/C	IT4var39	5.84E-42	Group B/C	AAB60251	4.54E-38	Group B/C
	6	MW014742	6003	5	3	264.5	DC8	KX154877.1	6.01E-116	DC8	KX154847.1	2.29E-75	DC8	KX154898.1	2.29E-75	DC8	PF08_0140	8.65E-40	EPCR	RAJ116var06	1.45E-37	Group B/C
	7	MW014743	2934	5	5	191.0	Group B/C	PFCLINvar41	2.96E-40	Group B/C	RAJ116var30	1.06E-39	Group B/C	HB3var13	1.06E-39	Group B/C	RAJ116var34	4.95E-38	Group B/C	PFE0005w	6.41E-37	Group B/C
	9	MW014744	2264	5	3	111.4	Group B/C	IT4var64	3.60E-44	Group A-Rosetting/Unknown	DD2var52	3.60E-44	Group A-Rosetting/Unknown	KX154955.1	4.66E-43	Group B/C	KX154896.1	6.07E-37	Group B/C	PFE0005w	1.31E-33	Group B/C
	10	MW014745	2255	5	4	215.6	Group B/C	RAJ116var23	9.27E-65	Group B/C	RAJ116var01	9.27E-65	Group B/C	KX154960.1	9.47E-50	EPCR	IT4var23	9.54E-45	Group B/C	IT4var62	3.43E-44	Group B/C
	11	MW014746	2086	5	4	335.8	EPCR	PFCLINvar62	9.95E-109	EPCR	IT4var60	1.70E-91	Group A-Rosetting/Unknown	KX154924.1	1.33E-82	EPCR	KX154874.1	1.74E-76	EPCR	KX154830.1	6.29E-71	EPCR
	12	MW014747	1355	5	4	281.4	EPCR	PFCLINvar49	1.68E-91	EPCR	KX154911.1	6.27E-66	EPCR	IGHvar09	6.27E-66	EPCR	PFI1820w	4.89E-62	Type 3 var	KX154845.1	6.32E-61	EPCR
	14	MW014748	1013	5	4	107.3	Group B/C	PFCLINvar31	1.16E-33	DC8	KX154864.1	6.97E-31	Group B/C	MAL7P1.50	1.17E-28	Group B/C	KX154900.1	5.43E-27	Group B/C	DD2var29	1.17E-23	Group B/C
117	1	MW014749	68783	5	5	195.1	Group B/C	KX154971.1	1.60E-47	Group B/C	TM284S2var1	5.79E-42	Group B/C	KX154828.1	5.83E-37	Group B/C	IGHvar17	5.83E-37	Group B/C	IGHvar40	2.71E-35	Group B/C
	2	MW014750	5749	5	5	303.0	Group B/C	IGHvar40	4.11E-73	Group B/C	IT4var45	6.93E-66	Group B/C	IT4var62	5.40E-62	Group B/C	KX154971.1	1.18E-53	Group B/C	AY028643	5.47E-52	Group B/C
	3	MW014751	5265	5	5	226.8	Group B/C	IT4var23	1.21E-58	Group B/C	PFL1960w	5.70E-47	Group B/C	IT4var54	5.74E-42	Group B/C	PFCLINvar65	2.06E-41	Group B/C	IT4var59	2.06E-41	Group B/C
	4	MW014752	4048	5	5	173.7	Group B/C	KX154906.1	1.06E-63	Group B/C	HB3var48	2.39E-30	Group B/C	PFL0005w	3.10E-29	Group B/C	DD2var33	3.10E-29	Group B/C	RAJ116var22	8.67E-25	Group B/C
	5	MW014753	3718	5	5	285.0	Group B/C	KX154832.1	2.69E-74	Group B/C	HB3var12	4.50E-72	Group B/C	HB3var40	2.15E-50	Group B/C	KX154866.1	6.03E-46	Group B/C	HB3var14	6.03E-46	Group B/C
	6	MW014754	3158	5	5	214.6	Group B/C	PFL0935c	1.51E-57	Group B/C	IGHvar03	1.19E-43	Group B/C	RAJ116var09	5.55E-42	Group B/C	IGHvar17	1.20E-38	Group B/C	KX154940.1	2.01E-36	Group B/C
	7	MW014755	2197	5	5	1513.6	var1csa	DD2var25	0	var1csa	AJ420411	0	var1csa	KX154917.1	9.01E-179	var1csa	IGHvar11	5.46E-171	var1csa	AAM55194	5.50E-166	var1csa
	8	MW014756	1712	5	5	232.4	Group B/C	IGHvar04	3.33E-59	Group B/C	PF07_0049	2.61E-50	Group B/C	PFD0625c	4.37E-48	Group B/C	DD2var24	9.53E-40	Group B/C	IT4var62	1.23E-38	Group B/C
	9	MW014757	1635	5	5	179.6	Group B/C	TM284S2var1	5.45E-42	Group B/C	KX154828.1	5.49E-37	Group B/C	IGHvar17	5.49E-37	Group B/C	IT4var67	9.19E-35	Group B/C	RAJ116var22	1.54E-32	Group B/C
	10	MW014758	1323	5	5	177.4	Group B/C	PFA0005w	8.97E-40	Group B/C	IT4var47	1.16E-38	Group B/C	RAJ116var13	9.03E-35	Group B/C	DD2var16	3.25E-34	Group B/C	PFCLINvar29	1.17E-33	Group B/C
	11	MW014759	1131	5	5	285.8	Group B/C	IGHvar40	1.14E-73	Group B/C	IT4var62	1.94E-61	Group B/C	KX154819.1	5.43E-57	Group B/C	AY028643	3.29E-49	Group B/C	IT4var45	4.26E-48	Group B/C
	12	MW014760	927	5	5	236.3	Group B/C	IT4var45	8.96E-65	Group B/C	IT4var39	4.23E-53	Group B/C	IGHvar40	4.29E-43	Group B/C	IT4var23	5.55E-42	Group B/C	HB3var28	5.59E-37	Group B/C
	13	MW014761	923	5	5	196.2	Group B/C	IT4var17	2.57E-55	Group B/C	PFL1950w	2.04E-36	Group B/C	HB3var23	2.04E-36	Group B/C	PFE0005w	7.35E-36	Group B/C	PF13_0001	7.35E-36	Group B/C
	14	MW014762	711	5	5	208.0	Group B/C	HB3var30	2.45E-65	Group B/C	DD2var45	1.52E-42	Group B/C	PFD1000c	9.19E-35	Group B/C	PFD0995c	9.19E-35	Group B/C	IGHvar18	3.31E-34	Group B/C
118	1	MW014763	41084	5	5	304.9	EPCR	KX154936.1	1.04E-73	EPCR	PFCLINvar62	8.06E-70	EPCR	RAJ116var03	6.32E-61	EPCR	KX154871.1	1.38E-52	EPCR	IGHvar14	1.78E-51	EPCR
	2	MW014764	17016	5	3	154.0	DC8	KX154933.1	9.77E-59	DC8	DD2var29	2.12E-55	Group B/C	KX154864.1	7.66E-50	Group B/C	KX154942.1	9.91E-49	DC8	MAL6P1.316	9.91E-49	DC8
	3	MW014765	16853	5	3	130.5	Group B/C	PFCLINvar05	8.82E-70	Group A-Rosetting/Unknown	DD2var48	3.24E-54	Group B/C	KX154829.1	2.54E-45	Group B/C	IGHvar09	1.19E-38	EPCR	PFCLINvar39	4.31E-33	Group B/C
	4	MW014766	15648	5	5	280.6	Group B/C	KX154956.1	3.37E-74	Group B/C	IGHvar02	9.57E-60	Group B/C	KX154832.1	3.47E-54	Group B/C	PF13_0364	2.70E-50	Group B/C	PFE0005w	7.56E-46	Group B/C
	5	MW014767	8388	5	3	226.2	DC8	KX154953.1	2.63E-104	DC8	KX154885.1	4.65E-62	DC8	KX154939.1	4.65E-62	DC8	RAJ116var32	2.82E-54	Group B/C	KX154894.1	1.32E-47	Group B/C
	6	MW014768	5932	5	5	150.9	Group B/C	HB3var25	2.28E-65	Group B/C	PFCLINvar25	3.98E-33	Group B/C	IT4var26	1.12E-23	Group B/C	PF11_0007	5.26E-17	Group B/C	DD2var26	2.45E-15	Group B/C
	7	MW014769	5363	5	5	247.5	Group B/C	RAJ116var39	3.07E-54	Group B/C	PFCLINvar47	6.65E-51	Group B/C	DD2var39	8.61E-50	Group B/C	PFL1960w	3.10E-49	Group B/C	PFCLINvar27	5.18E-47	Group B/C
	8	MW014770	1773	5	5	218.6	Group B/C	RAJ116var06	1.88E-51	Group B/C	PF10_0406	2.45E-45	Group B/C	KX154934.1	8.83E-45	Group B/C	RAJ116var14	2.47E-40	Group B/C	DD2var23	2.47E-40	Group B/C
	9	MW014771	1659	5	3	221.2	EPCR	KX154966.1	2.17E-80	EPCR	KX154869.1	1.30E-77	EPCR	RAJ116var05	2.18E-75	Group A-Rosetting/Unknown	DD2var22	1.32E-67	Group A-Rosetting/Unknown	RAJ116var03	2.21E-65	EPCR
	10	MW014772	1314	5	5	231.6	Group B/C	DD2var37	6.10E-51	Group B/C	IGHvar05	1.02E-48	Group B/C	IT4var11	1.71E-46	Group B/C	PFCLINvar74	7.95E-45	Group B/C	KX154972.1	2.86E-44	Group B/C

	11	MW014773	1064	5	5	243.5	Group B/C	RAJ116var09	3.33E-54	Group B/C	KX154853.1	3.35E-49	Group B/C	KX154971.1	1.20E-48	Group B/C	IT4var30	1.20E-48	Group B/C	KX154819.1	2.02E-46	Group B/C
	12	MW014774	980	5	3	141.7	Group B/C	PFL1950w	1.09E-48	Group B/C	HB3var23	1.09E-48	Group B/C	PFD0615c	1.83E-46	Group B/C	KX154827.1	8.51E-45	DC8	KX154919.1	3.96E-43	DC8
	13	MW014775	962	5	4	295.3	EPCR	KX154890.1	1.03E-83	EPCR	IGHvar24	1.05E-73	Group A-Rosetting/Unknown	KX154923.1	4.86E-72	EPCR	KX154937.1	4.86E-72	EPCR	KX154857.1	2.26E-70	EPCR
	14	MW014776	881	5	5	451.6	Group B/C	PFCLINvar44	5.68E-136	Group B/C	DD2var46	5.68E-136	Group B/C	HB3var40	1.74E-66	Group B/C	MAL7P1.212	8.17E-60	Group B/C	HB3var20	4.92E-57	Group B/C
123	1	MW014777	36995	5	5	158.0	Group B/C	PF08_0103	7.11E-36	Group B/C	PFCLINvar11	2.56E-35	Group B/C	IGHvar33	2.56E-35	Group B/C	PFCLINvar56	1.55E-27	Group B/C	HB3var07	1.55E-27	Group B/C
	2	MW014778	25414	5	5	226.6	Group B/C	KX154881.1	1.04E-54	Group B/C	IT4var16	1.04E-54	Group B/C	HB3var19	1.37E-43	Group B/C	DD2var23	8.23E-41	Group B/C	PFCLINvar22	2.30E-36	Group B/C
	4	MW014779	21424	5	3	158.2	DC8	KX154825.1	6.88E-71	DC8	KX154946.1	1.95E-56	DC8	IT4var39	1.55E-37	Group B/C	DD2var49	2.60E-35	EPCR	KX154870.1	4.35E-33	DC8
124	1	MW014780	25464	5	5	219.9	Group B/C	IT4var45	8.05E-51	Group B/C	IGHvar40	1.74E-47	Group B/C	DD2var34	1.76E-42	Group B/C	PFD0625c	2.27E-41	Group B/C	HB3var28	2.27E-41	Group B/C
	2	MW014781	8211	5	5	151.1	Group B/C	IGHvar16	1.66E-37	Group B/C	IT4var63	4.65E-33	Group B/C	IGHvar21	1.01E-29	Group B/C	PFL1960w	4.68E-28	Group B/C	DD2var10	2.18E-26	Group B/C
	3	MW014782	7991	5	4	226.3	Group B/C	PFCLINvar28	2.30E-85	Group B/C	KX154848.1	1.85E-56	Group B/C	AAQ73929	5.23E-47	Type 3 var	HB3var16	3.15E-44	Group B/C	RAJ116var22	4.07E-43	Group B/C
	4	MW014783	6137	5	5	1151.6	Group B/C	DD2var41	0	Group B/C	DD2var07	0	Group B/C	HB3var16	8.81E-65	Group B/C	KX154881.1	1.50E-52	Group B/C	RAJ116var21	1.98E-36	Group B/C
	5	MW014784	5837	5	4	207.8	Group B/C	KX154934.1	2.52E-75	Group B/C	DD2var45	5.65E-47	Group B/C	PFCLINvar66	2.63E-45	Group B/C	DD2var29	4.40E-43	Group B/C	KX154861.1	1.23E-38	DC8
	6	MW014785	4325	5	5	225.1	Group B/C	KX154955.1	5.59E-72	Group B/C	PFD1245c	2.72E-40	Group B/C	HB3var28	2.72E-40	Group B/C	KX154910.1	1.26E-38	Group B/C	IT4var33	1.63E-37	Group B/C
	7	MW014786	3666	5	5	311.9	Group B/C	KX154849.1	5.03E-112	Group B/C	DD2var23	1.93E-66	Group B/C	PFCLINvar08	1.97E-51	Group B/C	RAJ116var06	4.29E-43	Group B/C	DD2var31	1.54E-42	Group B/C
	8	MW014787	3308	5	5	231.5	Group B/C	PFCLINvar27	3.78E-63	Group B/C	DD2var30	1.07E-48	Group B/C	HB3var07	1.40E-42	Group B/C	RAJ116var10	5.03E-42	Group B/C	KX154916.1	1.09E-38	Group B/C
	9	MW014788	2178	5	4	158.5	Group B/C	PFCLINvar45	8.53E-50	Group B/C	IT4var01	6.69E-41	Group B/C	KX154824.1	2.42E-35	Group B/C	IT4var40	2.42E-35	Group B/C	PFCLINvar31	1.13E-33	DC8
	10	MW014789	1977	5	5	267.9	Group B/C	IGHvar08	5.45E-67	Group B/C	RAJ116var21	5.49E-62	Group B/C	IT4var10	1.20E-48	Group B/C	AAC05220	1.20E-48	Group B/C	DD2var34	2.61E-45	Group B/C
	11	MW014790	1838	5	4	359.2	EPCR	IT4var07	5.99E-106	EPCR	PF11_0521	4.69E-97	EPCR	IT4var08	2.20E-90	Group A-Rosetting/Unknown	KX154945.1	1.02E-88	EPCR	KX154903.1	2.26E-70	EPCR
	12	MW014791	1630	5	5	218.4	Group B/C	PF07_0048	2.68E-60	Group B/C	RAJ116var36	4.59E-43	Group B/C	IT4var47	1.65E-42	Group B/C	IT4var34	2.76E-40	Group B/C	IT4var68	7.73E-36	Group B/C
	13	MW014792	1251	5	3	158.3	EPCR	PF11_0521	2.99E-74	EPCR	PFCLINvar33	1.10E-58	Group A-Rosetting/Unknown	DD2var09b	1.12E-43	Group A-Rosetting/Unknown	IT4var07	4.03E-43	EPCR	HB3var03	4.03E-43	EPCR
	14	MW014793	1188	5	5	310.0	Group B/C	KX154955.1	2.63E-90	Group B/C	HB3var47	2.75E-60	Group B/C	HB3var09	2.75E-60	Group B/C	PFD0630c	4.60E-58	Group B/C	PFD0625c	1.01E-44	Group B/C
	15	MW014794	1144	5	5	193.9	Group B/C	HB3var22	7.80E-46	Group B/C	IT4var45	7.86E-41	Group B/C	PFCLINvar41	4.73E-38	Group B/C	RAJ116var34	2.20E-36	Group B/C	HB3var28	2.20E-36	Group B/C
	16	MW014795	920	5	5	251.1	Group B/C	KX154884.1	5.85E-57	Group B/C	KX154850.1	2.11E-56	Group B/C	PFD0630c	2.12E-51	Group B/C	IGHvar31	2.76E-45	Group B/C	PFD0635c	9.94E-45	Group B/C
	17	MW014796	896	5	5	222.4	Group B/C	HB3var16	2.00E-51	Group B/C	DD2var38	2.00E-51	Group B/C	PF08_0142	1.56E-47	Group B/C	HB3var33	9.45E-40	Group B/C	KX154831.1	7.35E-36	Group B/C
	18	MW014797	877	5	5	245.0	Group B/C	IGHvar25	2.68E-60	Group B/C	KX154826.1	1.25E-58	Group B/C	PFI1830c	7.62E-46	Group B/C	HB3var24	7.62E-46	Group B/C	PFD0635c	4.62E-38	Group B/C
	19	MW014798	815	5	3	210.6	Group A-Rosetting/Unknown	KX154872.1	1.02E-78	EPCR	KX154843.1	6.13E-76	Group A-Rosetting/Unknown	RAJ116var17	6.17E-71	Group A-Rosetting/Unknown	IGHvar39	6.17E-71	EPCR	RAJ116var19	6.22E-66	Group A-Rosetting/Unknown
	20	MW014799	769	5	5	312.0	Group B/C	PFL0005w	2.25E-95	Group B/C	MAL6P1.1	6.61E-56	Group B/C	HB3var47	6.61E-56	Group B/C	HB3var09	6.61E-56	Group B/C	RAJ116var06	1.43E-52	Group B/C
	21	MW014800	749	5	5	140.7	Group B/C	RAJ116var14	9.36E-34	Group B/C	IGHvar40	4.35E-32	Group B/C	PFD0615c	1.57E-31	Group B/C	IT4var61	2.64E-24	Group B/C	PFCLINvar41	1.23E-22	Group B/C
	22	MW014801	705	5	3	213.4	EPCR	PFCLINvar68	3.64E-83	EPCR	RAJ116var16	7.87E-80	Group A-Rosetting/Unknown	RAJ116var19	7.98E-70	Group A-Rosetting/Unknown	KX154909.1	1.03E-68	EPCR	KX154911.1	1.04E-63	EPCR
	23	MW014802	602	5	4	134.6	Group B/C	HB3var16	8.57E-40	Group B/C	IGHvar20	5.16E-37	Group B/C	PFCLINvar30	1.12E-33	DC8	DD2var20	4.01E-33	Group B/C	DD2var48	1.45E-27	Group B/C
	24	MW014803	595	5	5	237.9	Group B/C	PFCLINvar66	2.06E-56	Group B/C	PFD0615c	4.45E-53	Group B/C	DD2var45	2.08E-46	Group B/C	PFCLINvar41	2.70E-45	Group B/C	PFI1830c	2.72E-40	Group B/C
	25	MW014804	548	5	5	332.8	Group B/C	IT4var30	7.27E-96	Group B/C	PFI1830c	1.26E-68	Group B/C	IGHvar40	2.75E-60	Group B/C	IT4var05	3.55E-59	Group B/C	KX154971.1	1.67E-52	Group B/C

	26	MW014805	472	5	5	849.2	var1csa	RAJ116var02	5.15E-171	var1csa	PFCLINvar76	5.15E-171	var1csa	AF547159	5.15E-171	var1csa	KX154858.1	1.85E-170	var1csa	KX154820.1	2.40E-169	var1csa
	28	MW014806	373	5	4	168.2	Group B/C	KX154934.1	6.08E-52	Group B/C	KX154861.1	4.77E-43	DC8	DD2var29	4.77E-43	Group B/C	IT4var45	7.98E-41	Group B/C	IGHvar13	2.89E-35	Group B/C
	29	MW014807	352	5	5	253.2	Group B/C	KX154849.1	2.21E-66	Group B/C	IT4var45	3.75E-54	Group B/C	RAJ116var34	2.27E-46	Group B/C	HB3var28	8.17E-46	Group B/C	DD2var10	3.80E-44	Group B/C

[a] The number of reads within a sequence cluster

[b] Only the top 5 hits from BLAST searches were considered when assigning a var type to DBLα tags

[c] The number of times that the var type with the highest cumulative score was observed among the top 5 hits

[d] The highest cumulative score, derived from BLAST E-values (sum of -log10(E-value)), of the var types represented in the top 5 BLAST hits

Supplemental Table 5. *var* domain expression levels in disease groups. Related to Figure 3.

Primer	Primer subset sum	Domain cassette	Binding	UM (N = 9)	CM (N = 18)	p-value	BS (N= 16)	pvalue	Adult CM/BS (N = 12)	Pediatric CM/BS (N = 9)	pvalue
DBLβ		DC5	CD31	1.4 (1, 2.3)	3.1 (1.1, 20.6)	0.223	2.4 (1, 25.4)	0.271	1.4 (1, 2.3)	22.2 (4.1, 45.5)	***0.019 (*)***
DBLγ		DC5	CD31	1 (1, 1)	1 (1, 4.9)	0.113	1.4 (1, 5.9)	0.075	1 (1, 2.3)	2.4 (1, 20.9)	0.176
DBLα1.5/6/8		DC16	Roset/ Unk.	12.2 (4, 29.8)	19.2 (3.8, 81.0)	0.616	39.5 (5, 93.5)	0.178	4.8 (2.14, 72.6)	39.1 (11.8, 94.3)	0.188
CIDRδ		DC16	Roset/ Unk.	2.2 (1, 7.9)	1.5 (1, 16.2)	1.000	2.3 (1, 13.7)	0.755	1.1 (1, 1.8)	5.3 (1.8, 62.7)	***0.044 (*)***
DBLα-CIDRα		DC8	EPCR	15.6 (8.1, 27.2)	20.1 (3, 66.9)	0.596	24.2 (4.7, 103.2)	0.461	14.5 (4.7, 52.3)	28.9 (18.7, 69.7)	0.498
CIDRα1.1		DC8	EPCR	5.4 (1, 12.1)	5.0 (1.2, 26.3)	0.612	7.6 (1, 34.7)	0.713	1.5 (1, 5.0)	29.2 (5.4, 36.8)	***0.027 (*)***
CIDRα1.8 (a+b)		DC8	EPCR	49.2 (12.4, 87.1)	17.6 (1.5, 53.9)	0.302	6.9 (1.1, 43.4)	0.244	3.9 (1.1, 18.1)	35.0 (2.7, 75.1)	0.154
	DC8 (CIDRα1.1/8)	DC8	EPCR	60.0 (13.4, 99.8)	36.2 (7.3, 71.7)	0.617	36.2 (4.8, 88.8)	0.759	13.8 (3.1, 38.3)	65.8 (34.1, 141.0)	0.058
DBLα2/1.1/2/4/7			EPCR	133.4 (72.9, 250.8)	218.3 (130.9, 485.8)	0.160	352.5 (81.4, 522.2)	0.192	169.4 (97.2, 204.7)	408.6 (385.1, 554.0)	**0.028 (*)**
DBLα1.7		DC13	EPCR	4.7 (1.7, 9.5)	10.2 (5.5, 31.7)	0.154	15.5 (8.7, 74.2)	**0.029 (*)**	11.6 (7.3, 36.2)	18.5 (6.3, 95.2)	0.544
CIDRα1.4/6		DC13	EPCR	1 (1, 1)	1 (1, 1)	1.000	1 (1, 1)	0.536	1 (1, 1)	1 (1, 1)	0.441
CIDRα1.5 (a+b)			EPCR	1.74 (1, 12.5)	1.7 (1, 15.2)	0.932	1.4 (1, 24.3)	0.729	1 .4 (1, 5.5)	1.7 (1, 41.6)	0.511
CIDRα1.6			EPCR	1 (1, 1.8)	1 (1, 4.6)	0.781	1 (1, 2.9)	0.891	1 (1, 3.5)	1 (1, 2.9)	0.937
CIDRα1.7			EPCR	3.2 (1.1, 12.9)	14.6 (2.3, 45.6)	0.302	20.7 (3.6, 46.6)	0.208	4.3 (1.0, 14.1)	44.5 (27.8, 87.6)	0.054
	Group A (CIDRα1)		EPCR	13.1 (2.3, 64.1)	29.9 (8.5, 102.0)	0.541	40.2 (14.5, 106.0)	0.391	14.1 (1.6, 48.5)	102.5 (27.8, 130.2)	***0.021 (*)***
	CIDRα1 all (DC8-Group A)		EPCR	79.2 (29.0, 150.6)	60.8 (31.7, 172.9)	1.000	69.4 (34.2, 197.0)	0.834	44.1 (12.4, 72.5)	194.9 (49.6, 240.7)	***0.015 (*)***
DBLβ (EPCR-ICAM-1)			ICAM-1	10.1 (1.9, 37.8)	48.7 (9.4, 180.2)	0.134	49.4 (19.2, 201.6)	0.086	42.3 (12.7, 107.8)	57.6 (20.8, 207.8)	0.602
DBLβ1/3-1			ICAM-1	1.8 (1, 10.2)	6.8 (1.8, 16.5)	0.245	6.8 (4.0, 21.5)	0.201	4.8 (1, 16.4)	6.9 (5.3, 16.9)	0.388
DBLβ1/3-2			ICAM-1	2.9 (2.1, 9.1)	25.9 (3.1, 68.8)	0.126	9.1 (2.5, 54.6)	0.326	9.5 (2.9, 48.2)	23.4 (3.4, 75.1)	0.803
	DBLβ1/3 all		ICAM-1	20.2 (4.5, 84.8)	111.7 (25.8, 267.1)	0.075	128.7 (23.1, 290.7)	0.066	75.5 (23.1, 159.5)	237.6 (85.7, 345.9)	0.310
DBLβ5 (Group B/ C)			ICAM-1	4.5 (1.9, 13.5)	1 (1, 3.2)	0.061	1 (1, 2.4)	***0.046 (*)***	1 (1, 4.4)	1 (1, 2)	1.000
DBLγ		DC6	Unk.	25.0 (1.2, 68.6)	13.8 (1, 29.4)	0.554	7.6 (1, 22.7)	0.556	2.7 (1, 16.4)	19.5 (2.3, 36.7)	0.158
DBLζ		DC6	Unk.	1.8 (1, 28.0)	1.8 (1, 17.8)	0.792	1.9 (1, 7.4)	0.824	1.4 (1, 3.2)	2.4 (1, 19.5)	0.395
DBLα0.1			CD36	1.1 (1, 2.4)	1.7 (1, 7.1)	0.402	2.0 (1, 4.7)	0.299	1 (1, 8.4)	2 (1.3, 3.5)	0.584
DBLα0.6/9			CD36	4.86 (1, 287.1)	6.5 (2.3, 73.7)	1.000	5.0 (1, 67.5)	0.708	3.3 (1.2, 25.5)	54.1 (5.9, 96.6)	0.108
CIDRα2/3/5/6/7/9/10			CD36	49.4 (10.6, 90.5)	16.0 (4.0, 33.7)	0.261	16.0 (8.8, 52.0)	0.383	10.7 (3.7, 16.5)	21.5 (12.8, 63.3)	0.169
CIDRα2.2			CD36	1.9 (1, 4.1)	2.2 (1, 21.3)	0.567	6.3 (1, 24.1)	0.330	2.2 (1, 7.7)	13.2 (1, 24.8)	0.492
DBLα0.16		DC19	CD36	9.0 (4.8, 11.4)	7.8 (1.1, 27.3)	0.846	4.4 (1.2, 29.5)	0.444	12.1 (3.8, 25.5)	1.3 (1, 28.7)	0.318
	Group B/C		CD36	74.2 (25.5, 450.7)	69.2 (25.3, 170.7)	0.765	91.7 (24.1, 174.3)	0.697	64.4 (23.7, 128.7)	148.6 (60.7, 181.7)	0.095

Values are listed as median (25th, 75th); p-values correspond to Mann-Whitney test between groups including CM *vs.* UM, BS *vs*. UM and Adult CM *vs*. Pediatric CM. Shaded rows correspond to values shown in Figure 3.
